# Gender differences in like-sex middle-aged twins: an extended network analysis of depressive symptoms, cognitive functions and leisure activities

**DOI:** 10.1192/j.eurpsy.2025.31

**Published:** 2025-03-12

**Authors:** Daiyan Zhang, Maria Semkovska

**Affiliations:** 1 DeFREE Research Cluster, Department of Psychology, University of Southern Denmark, Odense, Denmark

**Keywords:** cognitive functions, depressive symptoms, gender, leisure activities, monozygotic twins, network analysis

## Abstract

**Background:**

Depression affects twice as many women as men. Risk factors for depression certainly impact this difference, but their strong interconnectedness challenges the assessment of standalone contributions. Network models allow the identification of systematic independent relationships between individual symptoms and risk factors. This study aimed to evaluate whether the extended networks of depressive symptoms, cognitive functions, and leisure activities in like-sex twins differ depending on gender or zygosity.

**Methods:**

Twins, including 2,040 women (918 monozygotic and 1,122 dizygotic) and 1,712 men (730 monozygotic and 982 dizygotic), were selected from the Danish Twin Registry for having, along with their like-sex co-twin, completed measures of depressive symptoms, cognition, and leisure activities (physical, intellectual, and social). Network models were estimated and compared at three levels: co-twins to each other within groups defined by gender and zygosity; monozygotic to dizygotic twins within the same gender, and between genders.

**Results:**

No significant differences were observed when co-twins were compared to each other, regardless of the pair’s zygosity or gender, nor when monozygotic twins were compared to dizygotic twins within gender. However, the gendered networks differed significantly in global strength, structure, and partial correlations between specific depressive symptoms and risk factors, all indicating stronger connectedness in women relative to men.

**Conclusions:**

Environmental factors appear to best explain between-gender network differences. Women’s networks showed significantly stronger associations both among depressive symptoms and between depressive symptoms and risk factors (i.e., decreased cognition and leisure activities). Longitudinal research is needed to determine the causality and directionality of these relationships.

## Introduction

Depression, a leading global burden of disease contributor [1] with 20% lifetime prevalence, affects twice as many women as men [2]. An initial epidemiological meta-analysis [3] suggested an absence of gender differences in the heritability for depression, though a recently updated review showed that the specific behavioural genetics methods used determine whether or not such differences are found [4]. Given the disorder’s high heterogeneity, little evidence exists of single genetic or environmental causes explaining all depressive symptoms [5]. Genes certainly contribute to these causes, as monozygotic twins show higher concordance rates for depression diagnosis [3], while environmental factors are critical for triggering its polygenic liability [6, 7]; namely the inherited predisposition to develop depression that is conferred by the joined action of several “vulnerability” genes. Besides gene–environment interactions, multiple intertwined aetiologies can explain the gender differences, including hormones, genetically determined physiological stress responsiveness, and gender-associated environmental stress exposure [8]. Assessing concurrently these factors to determine their individual contributions is complex; however, it is generally accepted that they are directly expressed in people’s cognitive abilities and daily functioning, which in turn are significant predictors of depression [9, 10].

Cognitive dysfunction is a recognised feature of depression [11], both during an acute episode and following remission [12]. Individuals presenting with depressive symptoms engage less in physical, social, and intellectual leisure activities [13], while participation in leisure activities is consistently associated with better cognitive function and a lower risk of cognitive decline [14–18]. Moreover, gender differences exist not only in depression prevalence but also in leisure activity participation and in some cognitive functions. Socially, women tend to establish quicker stronger cooperation with others, while mens cooperation levels increase progressively as the activity develops [19]. Women engage less than men in physical activities [20] and face more barriers to exercise [21]. Traditional female-gender role responsibilities, such as childcare and domestic chores, impact negatively participation in physical exercise, and make prioritising one’s health more challenging [22–24]. Gender differences in cognitive function are generally non-significant, with the notable exceptions of women outperforming men in verbal fluency, and men outperforming women in 3D mental rotation [25].

Previous studies have rarely explored concomitantly the complex interactions among symptoms, cognitive functions, and leisure activities, or evaluated the unique contribution of each depression risk factor independently from its shared associations with other predictors. One study [13] employed structural equation modelling to assess these interrelationships concurrently but aggregated multiple variables into single measures for each of the studied categories. Similarly, another study [26] totalled the performance of five different cognitive domains into a single factor, despite their distinct associations with depressive symptoms. For example, executive function correlates strongly with fatigue, but not with other depressive symptoms (e.g., indecisiveness, appetite changes) [27], while loneliness is associated with memory but not with orientation [28]. Reducing distinct phenomena to merged single factors prevents the identification of independent interrelations between specific symptoms and specific risk factors. Moreover, neither study examined the effect of gender on those complex associations, despite known gender differences in most of the studied variables.

Conceptualising the interactions between depressive symptoms and risk factors as a network allows us to consider each element’s individual contribution to the entire system without resorting to the oversimplification of summing up different factors into one. Specifically, when constructing a network where each depressive symptom and risk factor represents a network node, the links (network edges) between each pair of nodes can be studied while accounting for the remaining associations. Traditional models assuming that depressive symptoms are an interchangeable representation of a common cause [5], or that cognitive functions stem from a latent single factor are currently challenged [29]. Conversely, network theory allows to analyse of the complexity of specific interactions among individual symptoms and various risk factors, revealing patterns and connections that cannot be distinguished with traditional methods [30].

Recently, twin data have been modeled using network analyses to explore the heritability of cognitive abilities [29], anxiety [31], and depression [32], but again, known differences between women and men were not considered. We aimed to evaluate the gender effects on extended networks of depressive symptoms, cognitive functions, and three types of leisure activities (intellectual, physical, and social) using like-sex monozygotic (MZ) and dizygotic (DZ) twin pairs. This method allows, with astrong control for shared genetic liability for depression, to determine the most influential (central) nodes in a network and how symptoms and risk factors may interact to possibly explain the gender differences in depression expression. Two variables known to significantly influence both depressive symptoms and cognitive function – namely, age [33, 34] and alcohol consumption [35] – were used as covariates. Specifically, the study first examined the differences between the two members of a twin pair within sub-groups defined by gender and zygosity (i.e., MZ women, DZ women, MZ men, DZ men). Secondly, the differences between MZ and DZ networks were assessed within each gender group. Finally, the extended networks of depressive symptoms of women and men were directly compared.

## Methods

### Study population

Participants were collected from two cohort studies of the Danish Twin Registry, the Middle Age Danish Twins (MADT) 2008 survey, and the MIddle Age Danish Twins (MIDT) 2008–2011 survey years [36]. They assessed, respectively, 2,400 and 10,276 Danish twins born between 1931 and 1969. Participants self-reported their gender as part of the demographic data collection. Both studies utilised identical measures for depressive symptoms, cognitive functions, and leisure activities [36]. The following inclusion criteria were applied: (a) both twins of a pair participated, (b) the pair was like-sex (same gender), and (c) the pair’s zygosity was clearly determined.

### Measures

The standardised battery of the MADT and MIDT studies [36] included nine depressive symptoms from the Cambridge Mental Disorders of the Elderly Examination [37], six neuropsychological tests to estimate cognitive functions, and a scale measuring the frequency of engagement in three types of leisure activities, eight intellectual, six physical and eight social. Age and alcohol consumption were included as covariates due to their established associations with other variables. See Supplementary Table S1 for all measures’ scoring methods and corresponding node names. Descriptive statistics were computed separately for women and men. Categorical variables (e.g., zygosity, education, marital status, and work status) were analysed using Chi-square tests (χ^2^). Independent samples t-tests were conducted to compare between genders for continuous variables.

### Network analysis

*Network estimation.* Gaussian Graphical Models (GGMs) with graphical Least Absolute Shrinkage and Selection Operator (Glasso) regularisation were used to estimate the undirected networks of the six nodes’ categories – 9 depressive symptoms, 6 cognitive functions, 8 intellectual activities, 6 physical activities, 8 social activities, and 2 covariates. Glasso was applied with Extended Bayesian Information Criterion (EBIC) for model selection [38]. This method estimates 100 models with varying levels of sparsity. The final model is selected based on the lowest EBIC value, determined by hyperparameter gamma (γ), we set at 0.5 to minimise the risk of including spurious edges. To meet the GGM normality requirement, while optimising the original data preservation, nonparanormal transformations were used to normalise variables with absolute skew values >1. Networks were drawn by Cytoscape 3.10.2.

*Network centrality.* For each node, centrality measures for strength and expected influence (EI) were calculated with standardised *z*-scores for each independent network. Strength represents the sum of absolute values of the node’s connections to neighboring nodes, while EI refers to the net sum, accounting for positive and negative values [39].

*The network stability* was quantified with the correlation stability coefficient (CS-coefficient). It represents the maximum number of cases that can be dropped to retain a centrality correlation of at least 0.7. CS-coefficient values above 0.25 indicate stable networks, although traditionally, values above 0.5 are preferable. Edge accuracy was estimated with bootstrapped 95% confidence intervals (CIs), with narrower CIs suggesting more reliable networks. Bootstrapped difference tests were also performed to evaluate if centrality and edge-weights were stable [38].

*The network comparison test (NCT)* with 1000 permutations [40] was used for all between-networks comparisons, using indices of global strength invariance (*S*) and maximum difference (*M*) of network invariance. Firstly, differences between co-twin pairs were examined within each of the following sub-groups obtained by crossing gender with zygosity: MZ women, DZ women, MZ men, and DZ men. To mitigate randomness due to arbitrary assignment, we reassigned each of these datasets 1,000 times and obtained two averaged networks representing the independent networks of each co-twin [32]. For every reassignment iteration, the NCT evaluated differences in global strength and structure with *S* and *M* indices. The 1,000 *p*-values distribution of each index was used to identify significant differences among the reassignments. Secondly, MZ and DZ networks were compared within each gender group. Finally, overall differences between the two genders were assessed.

## Results

### Participants

**Figure 1.**
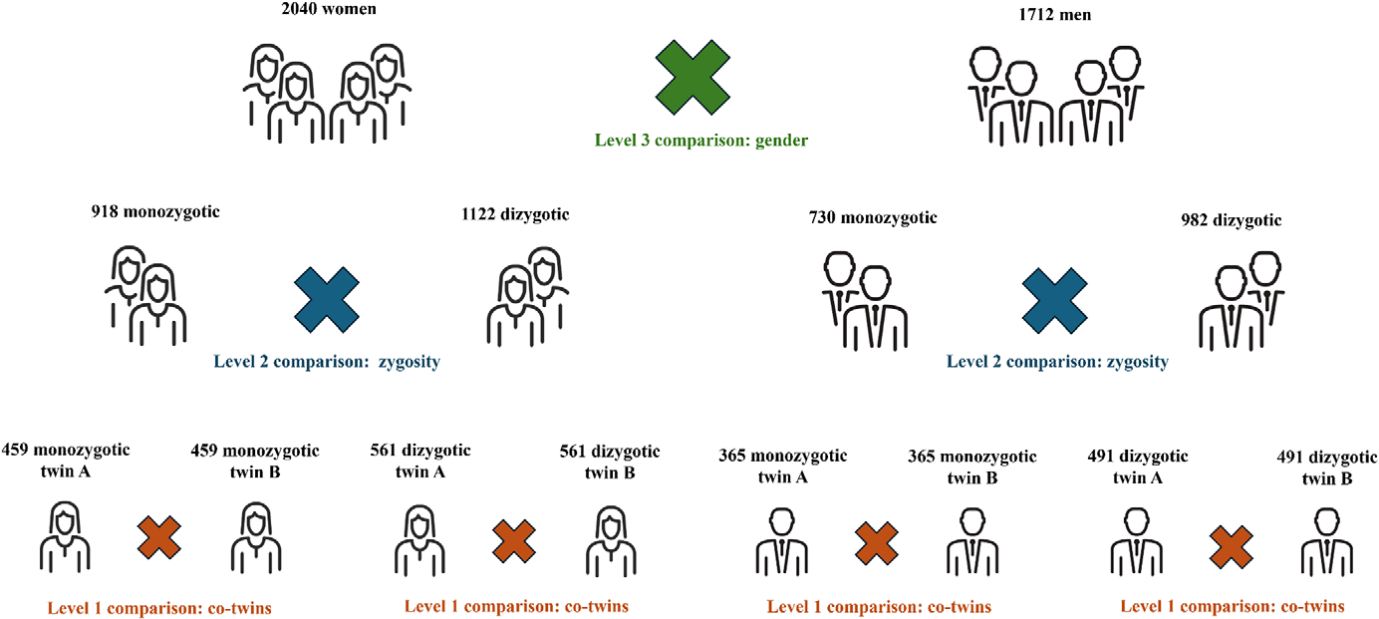
Diagram of the network comparisons conducted at three levels of analysis: (1) between co-twins of the same zygosity, (2) between zygosity types, and (3) between genders.

Applying the inclusion criteria to the merged MADT and MIDT databases led to the identification of 1,876 twin pairs (*N* = 3,752), consisting of 2,040 women (918 MZ and 1,122 DZ) and 1,712 men (730 MZ and 982 DZ). Table 1 presents the socio-demographic characteristics of the sample. Figure 1 illustrates the three-level sample’s subdivisions as required by the network analysis design.

**Table 1.** Descriptive statistics of the sample and between-gender differences

Variable	Women (*n* = 2040)		Men (*n* = 1712)		Statistic^a^	*p*-Value
**Zygosity**					*χ* ^2^ (1) = 2.01	0.294
MZ	918		730			
DZ	1122		982			
**Education**					*χ* ^2^ (6) = 117.00	0.000***
Master	139		209			
Bachelor	600		340			
Tertiary	88		77			
Post-secondary non-tertiary	119		35			
Upper secondary	566		609			
Lower secondary	283		212			
Missing values	245		230			
**Marital status**					*χ^2^* (5) = 55.42	0.000***
Married/cohabitating	1533		1430			
Divorce	220		118			
Separated	33		15			
Widow	125		43			
Never been married/cohabitating	116		93			
Missing values	13		13			
**Work status**					*χ^2^* (7) = 218.04	0.000***
Full-time	849		1012			
Part-time	379		90			
Early retired	167		108			
Retired because of special reasons	112		48			
Retired	376		349			
Sick for >14 days	31		13			
Other	101		78			
Missing values	25		14			
	**Mean**	**SD**	**Mean**	**SD**	***t* (3750)**	***p*-Value**
**Age** ^b^	56.76	9.84	59.24	9.55	−7.81	0.000***
						
	**Mean**	**SD**	**Mean**	**SD**	***t* (3750)**	***p*-Value**
**Units of weekly alcohol** **Consumption**	5.45	5.76	10.73	9.98	−20.07	0.000***
**Depressive symptoms**	**Mean**	**SD**	**Mean**	**SD**	***t^a^* (3750)**	***p*-Value**
HappyNow^c^	1.12	0.33	1.10	0.31	1.73	0.000***
HappyFre^c^	1.23	0.45	1.16	0.40	4.69	0.084
Lonely	1.39	0.55	1.24	0.45	8.50	0.000***
Tense	1.23	0.45	1.14	0.37	6.28	0.000***
Nervous	1.09	0.29	1.05	0.22	4.89	0.000***
Sad	1.16	0.39	1.07	0.25	7.99	0.000***
WorthNothing	1.28	0.49	1.25	0.46	1.89	0.000***
Outlook	3.15	1.22	2.90	1.35	4.80	0.0582
WorthLiving	1.06	0.23	1.03	0.18	3.22	0.001**
**Cognitive functions**	**Mean**	**SD**	**Mean**	**SD**	***t^a^* (3750)**	***p*-Value**
Learning	6.37	1.68	5.64	1.55	12.19	0.000***
AuditAtt	5.64	1.20	5.72	1.30	−1.89	0.059
WorkMemo	4.39	1.32	4.49	1.47	−1.73	0.083
CategoryFluency	24.84	6.34	23.76	6.27	3.50	0.000***
DelayedRecall	6.36	2.43	5.01	2.15	16.10	0.000***
PsychMotorSpeed	30.55	10.25	30.11	11.54	2.66	0.008**
**Intellectual activities**	**Mean**	**SD**	**Mean**	**SD**	***t^a^* (3750)**	***p*-Value**
Museum	1.98	0.53	1.96	0.54	2.75	0.006**
Library	3.65	1.16	3.77	1.24	−4.66	0.000***
Sudoku	2.94	1.39	2.40	1.41	13.10	0.000***
Books	3.72	1.15	3.48	1.15	6.82	0.000***
Courses	2.13	0.70	2.02	0.67	4.70	0.000***
WriteStory	1.93	1.16	1.86	1.17	1.49	0.136
Cinema	2.15	0.51	2.06	0.50	4.69	0.000***
Newspaper	4.40	0.88	4.59	0.74	−4.85	0.000***
**Physical activities**	**Mean**	**SD**	**Mean**	**SD**	***t^a^* (3750)**	***p*-Value**
Exercised	3.15	1.22	2.90	1.35	4.80	0.000***
BriskWalk	3.42	1.13	3.05	1.24	11.13	0.000***
Bicycle	2.98	1.31	2.94	1.28	0.88	0.379
Yoga	2.68	1.36	2.17	1.37	11.61	0.000***
HardWork	2.91	1.23	3.11	1.18	−5.28	0.000***
Sport	1.96	1.21	2.13	1.27	−5.45	0.000***
**Social activities**	**Mean**	**SD**	**Mean**	**SD**	***t^a^* (3750)**	***p*-Value**
GoParty	2.70	0.66	2.65	0.64	2.01	0.044*
Restaurant	2.44	0.60	2.36	0.56	3.58	0.000***
Phone	4.18	0.68	3.86	0.77	12.98	0.000***
Diner	2.62	0.60	2.50	0.60	6.40	0.000***
FriendsDiner	2.89	0.65	2.77	0.65	5.52	0.000***
Association	2.32	0.92	2.49	0.97	−5.48	0.000***
MeetTwin	4.75	0.92	4.55	0.96	5.46	0.000***
PhoneTwin	5.47	1.04	4.78	1.11	18.72	0.000***

Note: *p < 0.05, **p < 0.01, ***p < 0.001.

a Controlled for age and alcohol consumption.

b Age range: women, 40.28–79.53; men, 40.29–79.93.

c Reverse coded.

### Network comparisons of co-twins within gender by zygosity sub-groups

Networks were constructed and compared between co-twins within each zygosity-gender pair. All networks were stable. After the 1,000 reassignments, the *p*-values for the between-co-twins comparisons did not show any significant differences across all four groups in terms of global strength and network invariance, with all *p*-values >0.94, as shown in Supplementary Table S2. For MZ/DZ women, and MZ/DZ men, see Supplementary Figures S1, S7, S13, S19 for networks, Figures S2, S8, S14, S20 for centrality, Figures S3–S6, S9–S12, S15–S18, S20–S24, and Table S3 for stability.

### Network comparisons of MZ and DZ within each gender

After confirming the absence of significant differences between co-twins, sub-groups were merged to compare MZ to DZ networks within each gender, i.e., MZ women vs DZ women and MZ men vs DZ men. All networks were stable. In women, MZ and DZ networks were comparable in both global strength (*S* = 1.82, *p* = 0.210) and structure (*M* = 0.11, *p* = 0.483). Similarly, in men, MZ and DZ networks did not differ significantly neither in global strength (*S* = 1.11, *p* = 0.441), nor in structure (*M* = 0.16, *p* = 0.227), as shown in Supplementary Figure S27, S34 and Table S4. For MZ and DZ in each gender, see Supplementary Figures S25, S32 for networks, S26 and S33 for centrality, S28-S31, S35-S38 and Table S5 for stability.

### Network comparisons between genders

**Figure 2.**
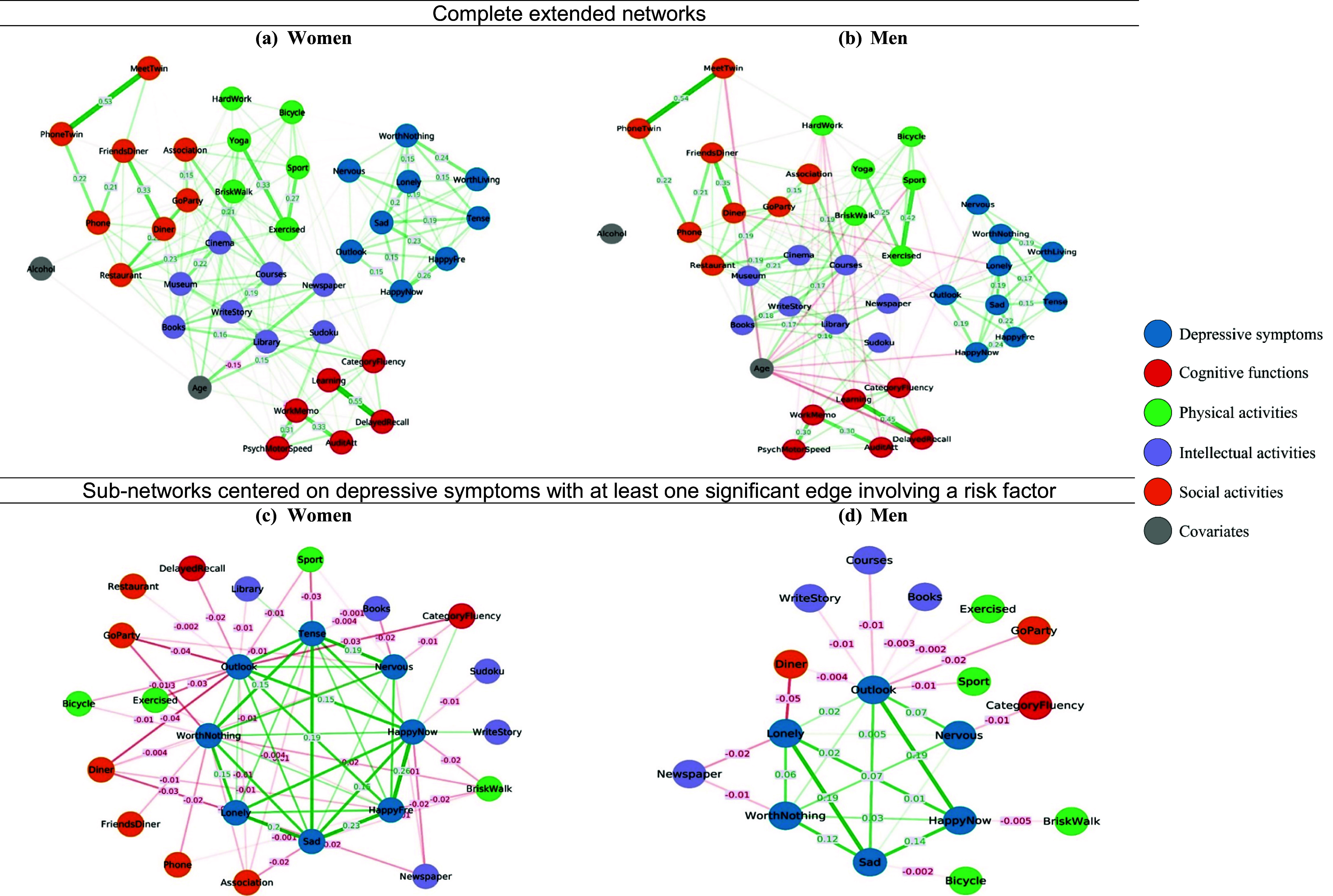
Women’s and men’s GGM networks of depressive symptoms, cognitive functions, frequency of leisure activities, and covariates. Green lines represent positive partial correlations, and red lines represent negative partial correlations.

*Networks’ stability.* Given the lack of significant zygosity effects, the MZ and DZ samples were further merged to construct two networks for women and men (Figure 2). The networks were highly stable, with CS-coefficient values of 0.75 (see Supplementary Figures S39–S42 and Table S6).

*Nodes’ centrality and edges.* See full results in Supplementary Figure S43 and Tables S7–S10. The gendered networks showed similar patterns in nodes’ centrality which reflects the importance of nodes (Supplementary Figure S43). For both, the same depressive symptoms, *Sad* (strength_women_ = 1.08, EI_women_ = 1.03; strength_men_ = 1.08, EI_men_ = 1.07) and *HappyNow* (strength_women_ = 1.03, EI_women_ = 0.86; strength_men_ = 0.92, EI_men_ = 0.79), and the same physical activity *Exercised* (strength_women_ = 1.05, EI_women_ = 0.86; strength_men_ = 1.02, EI_men_ = 0.97), were among the top five most influential nodes. The latter also included intellectual leisure activities, that was going to the *Museum* (strength = 1.01; EI = 1.01) for women, or to the *Library* (strength = 0.99; EI = 0.81), and to *Courses* (strength = 0.96; EI = 0.94) for men. The most central cognitive function was *DelayedRecall* (strength = 1.04; EI = 0.64) for women and *WorkingMemory* (strength = 0.86; EI = 0.83) for men.

The two genders showed similar edges with the top five absolute partial correlation values. These concerned cognitive functions – *Learning-DelayedRecall* (*pr*_women_ = 0.55; *pr*_men_ = 0.45), *WorkMemo-AuditAtt* (*pr*_women_ = 0.33; *pr*_men_ = 0.30), social activities – *MeetTwin-PhoneTwin* (*pr*_women_ = 0.53; *pr*_men_ = 0.54), *Dinner-FriendsDinner* (*pr*_women_ = 0.33; *pr*_men_ = 0.35), and physical leisure activities, where the edge with the highest value was *Yoga-Exercised* for women (*pr* = 0.33), and *Sport-Exercised* for men (*pr* = 0.42). All these correlations were positive, indicating strong connectivity within the corresponding risk factor’s individual nodes.

**Figure 3.**
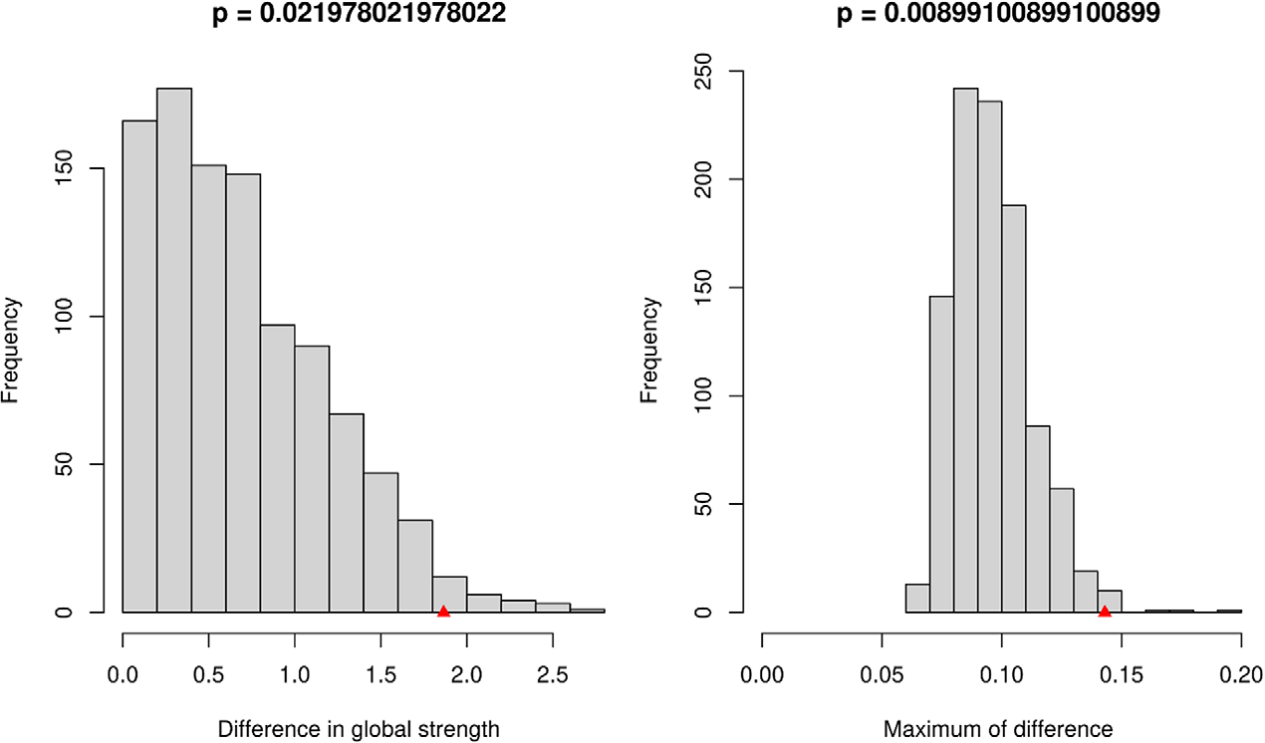
The network comparison test’s distribution of the 1,000 permutations of global strength and maximum difference indices in women and men. The red marker indicates the observed test statistic within the permutation test, highlighting its position to assess statistical significance.

*NCT.* Figure 3 showed that the gendered networks were significantly different in both global strength (*S* = 1.87, *p* = 0.022) and structure (*M* = 0.14, *p* = 0.009). Table 2 lists all nodes with significantly different between-gender centrality values. All indicated stronger connectivity of the corresponding nodes in women.

**Table 2.** Nodes with significant differences in strength centrality between women and men

Strength	Women	Men	*p*-value	Node category
WorthNothing	0.9465153	0.6203046	0.009**	Depressive symptom
Tense	0.8696782	0.6016601	0.009**	Depressive symptom
Yoga	0.7413204	0.4734702	0.003**	Physical activity
Museum	1.0094376	0.7608950	0.012*	Intellectual activity
Newspaper	0.6299685	0.3673111	0.007**	Intellectual activity
Restaurant	0.9653388	0.7509232	0.004**	Social activity
DelayedRecall	1.0370145	0.7964581	0.002**	Cognitive function

*Note.* *p < 0.05, **p < 0.01, ***p < 0.001.

When examining the edges connecting depressive symptoms with the remaining network nodes, 10 were found to be significant in women and only one in men. Table 3 details all edges involving depressive symptoms with significant between-gender differences. Non-significant edges within each gendered network are listed in Supplementary Tables S11–S12. Figure 1c and d visualize the gendered sub-networks centred on depressive symptoms and related risk factors to better illustrate where the core differences between women and men were concentrated. Two of these edges with significant differences concerned positive correlations between depressive symptoms. Specifically, women showed significantly stronger associations for *Lonely*-*WorthNothing* (*pr*_women_ = 0.151, *pr*_men_ = 0.065; *p* = 0.030) and for *Tense-Outlook* (*pr*_women_ = 0.118, *pr*_men_ = 0.019; *p* = 0.009). The remaining results concerned significantly stronger negative edges linking depressive symptoms with either cognitive functions or leisure activities. The only exception was the positive association *WriteStory*-*HappyNow.* Except Outlook-Courses, all these edges linking depressive symptoms with risk factors were null in men.

**Table 3.** Between-gender comparisons on edges with significant differences involving at least one depressive symptom

Edge	*pr* women	*pr* men	*p*-value	Category of the second node
Lonely-WorthNothing	0.1506603	0.0648693	0.030*	Depressive symptom
Tense-Outlook	0.1180386	0.0194005	0.009**	Depressive symptom
Outlook-Courses	0.0000000	−0.0124933	0.034*	Intellectual activities
HappyNow-WriteStory	0.0251031	0.0000000	0.009**	Intellectual activities
Outlook-Bicycle	−0.0139321	0.0000000	0.046*	Physical activities
Tense-Sport	−0.0285701	0.0000000	0.002**	Physical activities
HappyNow-Sudoku	−0.0074014	0.0000000	0.002**	Intellectual activities
HappyNow-Newspaper	−0.0221540	0.0000000	0.013*	Intellectual activities
HappyFre-Phone	−0.0009168	0.0000000	0.017*	Social activities
WorthNothing-Phone	−0.0170763	0.0000000	0.000***	Social activities
WorthNothing-FriendsDiner	−0.0111974	0.0000000	0.034*	Social activities
Sad-Association	−0.0185778	0.0000000	0.024*	Social activities
Outlook-CategoryFluency	−0.0336071	0.0000000	0.034*	Cognitive functions
Outlook-DelayedRecall	−0.0183782	0.0000000	0.016*	Cognitive functions

*Note.* ***pr***, partial correlation.

*p < 0.05, **p < 0.01, ***p < 0.001.

## Discussion

The study aimed to evaluate gender differences within and between like-sex co-twins. The observed networks were stable and without significant differences when co-twins were compared to each other, regardless of the pair’s zygosity or gender. Given these similarities, samples were pooled so to compare MZ to DZ women, and MZ to DZ men, leading to non-significantly different networks. However, when comparing the gendered networks (all women vs all men), significant differences in global strength, global structure, and local structure emerged. Specifically, women’s networks were denser (more interconnected) and showed significantly stronger associations both within depressive symptoms and between depressive symptoms and risk factors (i.e., cognition and leisure activities). Traditional models compare MZ and DZ twins to determine the relative contribution of genetic and environmental factors to the observed differences in variances/covariance within twin pairs [41]. Similarly, the network comparison test evaluates the overall differences in interconnectivity between co-twins first, and between MZ and DZ twins second, in order to examine these contributions at the network level [32]. Previous research using network analyses in MZ and DZ twins has not considered gender effects [29, 31, 32] and focused on within-symptoms [31] or within-cognitive functions [29] analyses. Overall, our results suggest that between-gender differences in the extended networks of depressive symptoms and the studied risk factors may be predominantly environmentally determined.

The gendered networks’ comparison revealed several significant differences. Among depressive symptoms, worthlessness and subjective tension were more central in women. Edges *worthlessness-loneliness* and *pessimistic outlook-subjective tension* were stronger in women. These results are consistent with both the established higher prevalence of depressive symptoms in women [2, 8] and the network theory of psychopathology, postulating that more densely connected symptom networks are associated with a stronger predisposition to depression [30].

Moreover, the women’s networks showed significantly stronger negative associations between depressive symptoms and physical or social leisure activities, including *worthlessness* - *frequency of calling family/friends*, *worthlessness* – *frequency of visiting family/friends for dinner*, and *subjective tension – strenuous sports engagement.* Subjective tension often indicates anxiety, which is closely related to a pessimistic view of the future [42]. This relationship is further evidenced by women being more likely to engage in rumination, a cognitive process that can perpetuate negative thinking and hinder the ability to maintain a positive outlook [42]. Socialisation and cultural expectations play a significant role in shaping how genders perceive their futures. Women are often socialised to prioritise the quality of relationships and emotional connectedness, which, can lead to feelings of worthlessness and pessimism regarding their future outlook [43]. This is further supported by the higher links’ strength of *worthlessness-loneliness*, *frequency of feeling happy* - *frequency of calling family/friends*, and *sadness*-*associations* in women relative to men. Research indicates that women exhibit greater emotional awareness and regulation skills, which can lead to a more nuanced view of their social world and own future [44]. However, this emotional intelligence may also result in heightened sensitivity to relationships’ quality and life’s uncertainties, potentially contributing to a more pessimistic outlook [44]. Social and cultural factors, including stereotyped expectations, could also underlie the stronger association between *subjective tension-strenuous sports engagement* in women. Body image concerns and traditional female domestic responsibilities can increase tension and impede physical activity prioritization [21], while the latter can prevent tension release.

Other connections linking depressive symptoms and leisure activities that showed significantly stronger, negative correlations in women relative to the absence of such correlations (zero values) in men, involved *pessimistic outlook* - *frequency of biking*, and *current unhappiness*, with intellectual activities. Regardless of gender, depression is linked to lower participation in physical and other leisure activities [13]. Moreover, women and men differ in how much they engage in physical activities, with women reporting more obstacles to exercising and less control over their exercise choices [21]. Interestingly, the only association that was significantly stronger in men concerned higher pessimistic outlook with lower engagement with courses. These results suggest that engaging in physical, intellectual, or social activities may be associated with fewer depressive symptoms, especially in women. However, our cross-sectional design precludes determining if this engagement directly intervenes in reducing depression. Nevertheless, the value of participation in leisure activities relative to depressive symptoms appears particularly relevant for women. Studies suggest that women may derive greater emotional and psychological benefits from social interactions and recreational activities, which could be due to the relational nature of women’s socialisation [45]. Gender differences in coping strategies also shape how individuals respond to stressors. Research indicates that women are more likely to engage in emotion-focused coping strategies, including seeking social support and participating in activities that promote emotional well-being [45]. This difference in coping styles might explain the reasons behind the stronger associations between leisure activities’ engagement and depressive symptoms in women relative to men.

Within both women’s and men’s networks, cognitive functions ranked among the most central elements and were strongly interconnected. However, few between-gender differences involved cognition. Specifically, *delayed verbal memory* was more central in women, whereas *pessimistic outlook* was more strongly connected negatively to both *delayed verbal memory* and *verbal fluency* in women. The associations between depressive symptoms and cognitive functions are known to be bidirectional [46], but in midlife, cognition does not predict future depressive symptoms, while depressive symptoms do predict lower future cognitive function, especially memory [46, 47]. Moreover, longitudinal research indicates that baseline memory is not associated with depression at follow-up in women aged over 65 [47]. Thus, our results may suggest that, in women, a pessimistic outlook could be the key bridge node through which depressive symptoms exercise their deleterious effect over long-term memory. However, this hypothesis needs to be verified by longitudinal research. Importantly, leisure activities appear overall more central to the extended networks of depressive symptoms than cognitive functions. Withdrawal from leisure activities is well-documented in depressed individuals [48]. Despite being cross-sectional, our findings allow to speculate that this association may emerge prior to a depression diagnosis, and be more prominent in women, supporting the hypothesis that intellectual, physical, and social leisure activities may serve as protective factors against depression [48, 49]. Nevertheless, future longitudinal studies are necessary to determine if such causal relationships exist.

The study has the following limitations. As research participants were middle-aged Danish twins, results are not necessarily generalisable to other ethnicities, age-groups, or broader populations. Moreover, contemporaneous networks based on GGMs do not possess causal inference capabilities. Therefore, significant associations should be interpreted with caution. The *HardWork* item primarily referred to family/work hard physical duties rather than leisure; nonetheless, we have retained it within the physical activities’ category to determine overall physical engagement. This inclusion did not significantly affect the results, as *HardWork* remained in the periphery of all networks. In future research, methodology such as longitudinal network analysis coupled with Bayesian network reasoning could be used to evaluate the directionality in observed significant edges, and thus further clarify gender differences. Our study did not evaluate the specific genetic, shared, and nonshared environmental portions of the studied variables’ variance. Future research could therefore apply traditional twin-design structural equation modelling (SEM) [41], alongside the network analyses, to determine the exact genetic and environmental contributions to the observed results. Our study did not include an assessment of lifestyle factors, which encompass a broad range of daily behaviours, such as smoking, substance consumption, and nutritional intake [50, 51]. Both leisure and lifestyle factors represent essential aspects of daily life, and can act as risk factors for depression. Network models of the relationship between lifestyle factors and depression have already been studied [50–52]. Future research should integrate both lifestyle factors and leisure activities for a more thorough assessment of their combined impact on depression.

Combining network analyses with a twin design enables the evaluation of the independent associations between depressive symptoms and risk factors, and the study of how gender may affect these relationships. This approach contributes towards current intervention science efforts towards the identification of the diverse mechanisms leading to depression, and the associated personalised targets for prevention [53]. Although the present novel findings require replication in other representative samples, they do indicate that personalised prevention should take gender into consideration and integrate the promotion of relevant leisure activities.

## Supporting information

Zhang and Semkovska supplementary material Zhang and Semkovska supplementary material

## Data Availability

In compliance with Danish and EU regulations, the transfer and dissemination of individual-level data from Danish registries require prior authorization from the Danish Data Protection Agency. Current local data protection policies prohibit the sharing of individual-level data in public databases. Data request can be directed through https://www.sdu.dk/en/forskning/dtr. Data access has been purchased from the Danish Twin Registry.

## Supplementary material

The supplementary material for this article can be found at http://doi.org/10.1192/j.eurpsy.2025.31.
