# Supplementary material for: Gender differences in like-sex middle-aged twins: an extended network analysis of depressive symptoms, cognitive functions and leisure activities
Source: Eur Psychiatry. 2025 Mar 12;68(1):e46. doi: 10.1192/j.eurpsy.2025.31 (PMC12041731; doi:10.1192/j.eurpsy.2025.31)
Supplement: Zhang and Semkovska supplementary material [file S0924933825000318sup001.pdf]

Gender Differences in Like-Sex Middle-Aged Twins: An extended network Analysis of Depressive Symptoms, Cognitive Functions and Leisure Activities

Zhang & Semkovska, 2025

Table of contents

Table S1 Nodes, measures and corresponding scores. 3
Table S2 Comparison of global strength and maximum difference of 1000 reassignments of co-twins within each pair of zygosity-gender dataset.......... 29
Table S3 Correlation stability coefficient of averaged network A and B in each zygosity-gender pair. 29
Table S4 Comparison of MZ and DZ in each gender 44
Table S5 Correlation stability coefficient of networks in each gender 44
Table S6 Correlation stability coefficient in women and men.......... 49
Table S7 Centrality of women network. 50
Table S8 Centrality of men network. 51
Table S9 Edges of women network.......... 52
Table S10 Edges of men network 58
Table S11 Non-significant edges connecting the depressive symptoms and other variables in women network.......... 63
Table S12 Non-significant edges connecting the depressive symptoms and other variables in men network.......... 64

Figure S1 Averaged network A and B in MZ women after 1000 reassignments. 5
Figure S2 Centrality (Strength and EI) of averaged network A and B in MZ women. 6
Figure S3 Stability of centrality indices of averaged network A and B in MZ women. 7
Figure S4 Stability of edges of averaged network A and B in MZ women. 8
Figure S5 Differences in centrality of averaged network A and B in MZ women. 9
Figure S6 Differences in edges of averaged network A and B in MZ women.......... 10
Figure S7 Averaged network A and B in DZ women after 1000 reassignments. 11
Figure S8 Centrality (Strength and EI) of averaged network A and B in DZ women. 12
Figure S9 Stability of centrality indices of averaged network A and B in DZ women.......... 13
Figure S10 Stability of edges of averaged network A and B in DZ women. 14
Figure S11 Differences in centrality of averaged network A and B in DZ women. 15
Figure S12 Differences in edges of averaged network A and B in DZ women.......... 16
Figure S13 Averaged network A and B in MZ men after 1000 reassignments.......... 17
Figure S14 Centrality (Strength and EI) of averaged network A and B in MZ men.......... 18
Figure S15 Stability of centrality indices of averaged network A and B in MZ men.......... 19
Figure S16 Stability of edges of averaged network A and B in MZ men.......... 20
Figure S17 Differences in centrality of averaged network A and B in MZ men. 21
Figure S18 Differences in edges of averaged network A and B in MZ men. 21
Figure S19 Averaged network A and B in DZ men after 1000 reassignments.......... 23
Figure S20 Centrality (Strength and EI) of averaged network A and B in DZ men.......... 24
Figure S21 Stability of centrality indices of averaged network A and B in DZ men. 25
Figure S22 Stability of edges of averaged network A and B in DZ men.......... 26

Figure S23 Differences in centrality of averaged network A and B in DZ men.................................... 27
Figure S24 Differences in edges of averaged network A and B in DZ men. 28
Figure S25 Networks of MZ and DZ in women. 30
Figure S26 Centrality (Strength and EI) comparisons of MZ and DZ in women. 31
Figure S27 Network comparison in global strength and network structure of MZ and DZ in women. 32
Figure S28 Stability of centrality indices in women. The top represents MZ twins and the bottom represents DZ. 33
Figure S29 Stability of edges in women. The top represents MZ twins and the bottom represents DZ.34
Figure S30 Differences in centrality of nodes in women. The top two represent MZ twins and the bottom represent DZ. 35
Figure S31 Differences in edges of women. The top represents MZ twins and the bottom represents DZ. 36
Figure S32 Networks of MZ and DZ in men. 37
Figure S33 Centrality (Strength and EI) comparisons of MZ and DZ in men. 38
Figure S34 Network comparison in global strength and network structure of MZ and DZ in men. 39
Figure S35 Stability of centrality indices in men. The top represents MZ twins and the bottom represents DZ 40
Figure S36 Stability of edges in men. The top represents MZ twins and the bottom represents DZ. ... 41
Figure S37 Differences in centrality of nodes in men. The top two represent MZ twins and the bottom represents DZ. 42
Figure S38 Differences in edges of men. The top represents MZ twins and the bottom represents DZ.43
Figure S39 Stability of centrality indices. The top represents women and the bottom represents men.45
Figure S40 Stability of edges. The top represents women and the bottom represents men. 46
Figure S41 Differences in centrality of nodes. The top two represent women and the bottom represent men. 47
Figure S42 Differences in edges. The top represents women and the bottom represents men. 48
Figure S43 Network comparison in centrality of women and men. 49

Measures of network nodes

Table S1 Nodes, measures and corresponding scores.

Node name	Items from questionnaires/tests
Depressive symptoms	
HappyNow (reversed)[a]	Are you happy with your life as it is now?
HappyFre (reversed)[b]	How often do you feel happy?
Lonely [b]	Have you been feeling lonely lately?
Tense [b]	Do you feel tense and are you more worried about trifles than you used to be?
Sad [b]	Do you feel sad, depressed or unhappy now?
Nervous[c]	Do you consider yourself to be nervous?
WorthNothing [b]	Do you feel like you're worth nothing at times?
Outlook[d]	How do look upon your future?
WorthLiving[c]	Do you sometimes feel that life is not worth living?
Cognitive functions	
PsychSpeed	Psychomotor processing speed (Digit Symbol Substitution)
AuditAtt	Auditory attention (Digit Span Forward)
WorkMemo	Working memory (Digit Span Backward)
Learning	Verbal learning abilities (Word Immediate Recall)
DelayedRecall	Delayed verbal memory (Word Delayed Recall)
Category fluency	Verbal Fluency
Intellectual activities	
Museum[e]	How often have you been to museums, art galleries or the like?
Library[e]	How often have you used the internet or a library or consulted an encyclopedia to find information that could answer a question or solve a problem?
Sudoku[e]	How often have you solved a sudoku, a cross-word puzzle or the like?
Books[e]	How often have you read a book, a news magazine or a technical report?
Courses[e]	How often have you attended a course, a study group or been to a public lecture?
WriteStory[e]	How often have you written a story, a report, a poem, an essay or kept a diary?
Cinema[e]	How often have you gone to the cinema, the theatre or a concert?
Newspaper[e]	How often have you read a newspaper?
Physical activities	
Exercised[e]	How often have you exercised, done aerobic or gymnastic exercises so that you perspired and/or became short of breath?
BriskWalk[e]	How often have you taken a brisk walk for at least 30 minutes in order to get exersice?
Bicycle[e]	How often have you ridden your bicycle for at least 3 km?
Yoga[e]	How often have you done stretch exercises, yoga, or pilates to increase suppleness, strength and a better balance?
HardWork[e]	How often have you been engaged in hard physical activity at work or at home?
Sport[e]	How often have you been engaged in physically strenuous sports like tennis, running, swimming or riding a bicycle over a long distance?

Social activities	
GoParty[e]	How often have you participated in a party or a social arrangement?
Restaurant[f]	How often have you been to a restaurant, to the cinema or the like together with friends or relatives?
Phone[e]	How often have you been in contact with family or friends over the phone or via mail?
Diner[e]	How often have you invited family or friends to dinner in your own home?
Friends Diner[e]	How often have you visited friends or family in their homes?
Associations[e]	How often have you participated in association meetings or meetings in general?
MeetTwin[g]	How often do you meet with your twin?
PhoneTwin[g]	How often do you speak with your twin over the phone?
Covariates	
Age	Participants' age at the time of the survey
Alcohol consumption	Alcohol consumption per week (multiply by 12 to get grams)

Note. [a] – scored on a scale ranging from 1 (*Always*) to 5 (*Never*), [b] – scored on a scale ranging from 1 (*No/Never*) to 3 (*Most/Mostly*), [c] - scored 1 (*No*) or 2 (*Yes*), d – scored 1 (*Optimistic*), 2 (*Neutral*) or 3 (*Pessimistic*), [e] - scored on scale ranging from 1 (*Never*) to 5 (*Daily*), [f] - scored on scale ranging from 1 (*Never*) to 4 (*Daily*), [g] - scored on scale ranging from 1 (*Never*) to 5 (*Daily*), [g] - scored on scale ranging from 1 (*Never*) to 7 (*Daily*).

Network analysis results of co-twins in each zygosity and gender

1. Results of MZ women

Figure S1 Averaged network A and B in MZ women after 1000 reassignments.

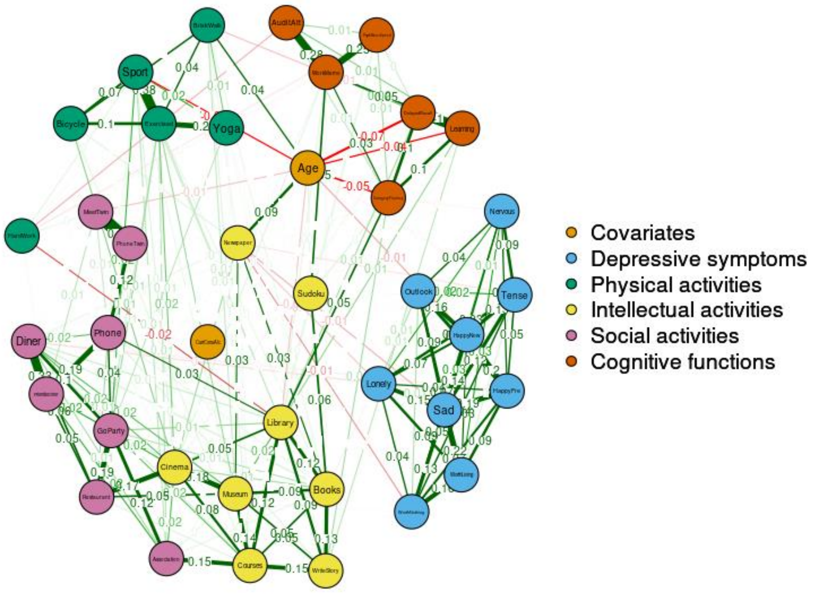

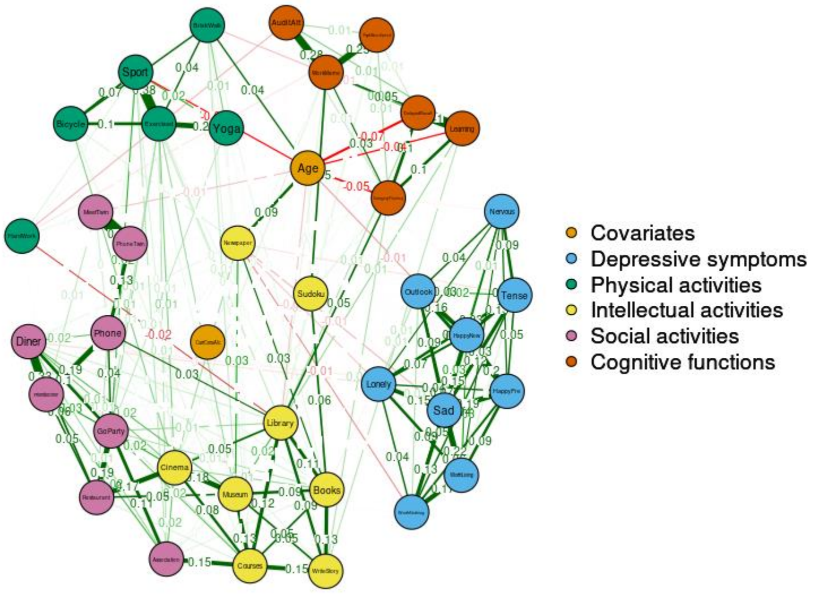

Figure S2 Centrality (Strength and EI) of averaged network A and B in MZ women.

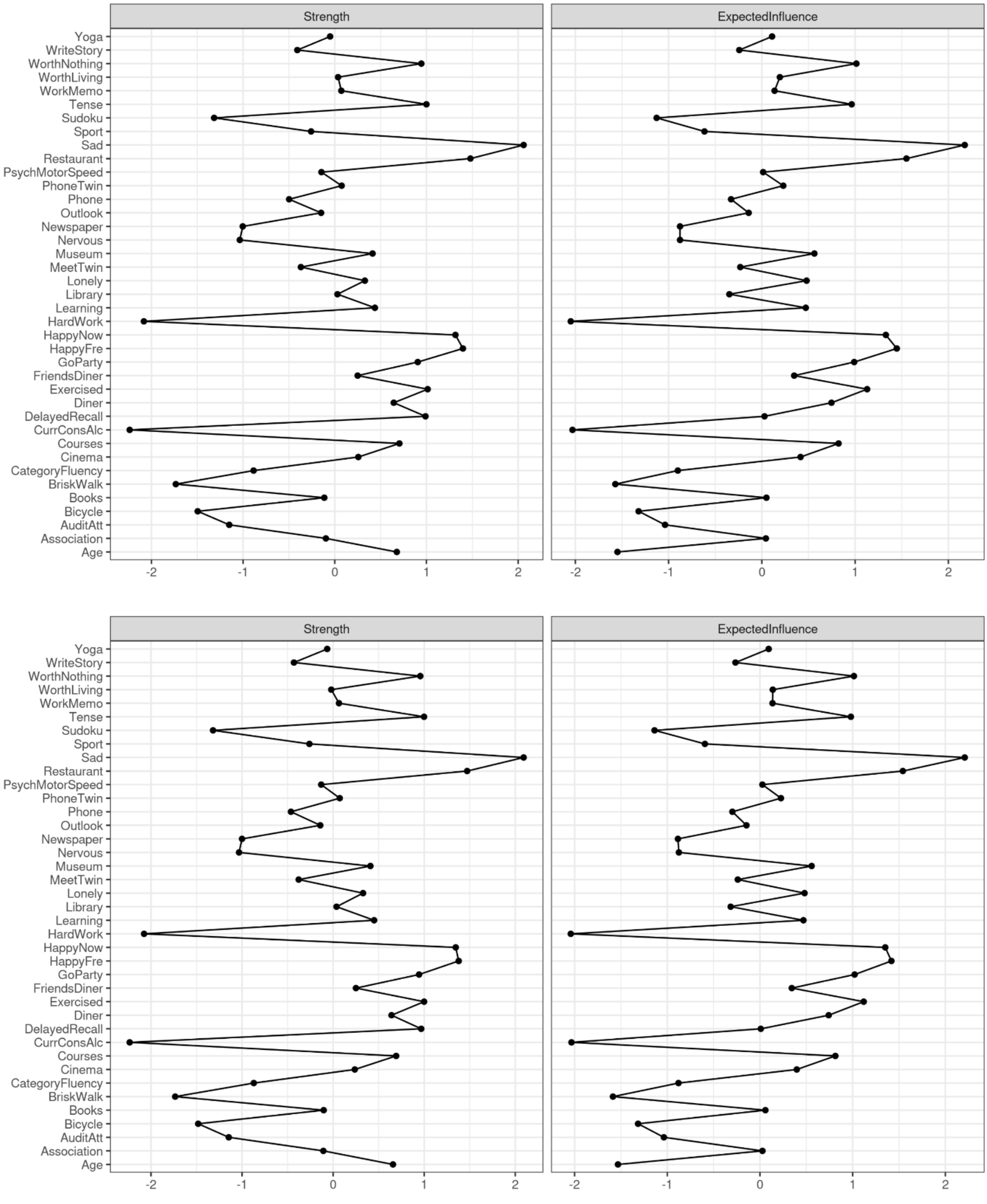

Figure S3 Stability of centrality indices of averaged network A and B in MZ women.

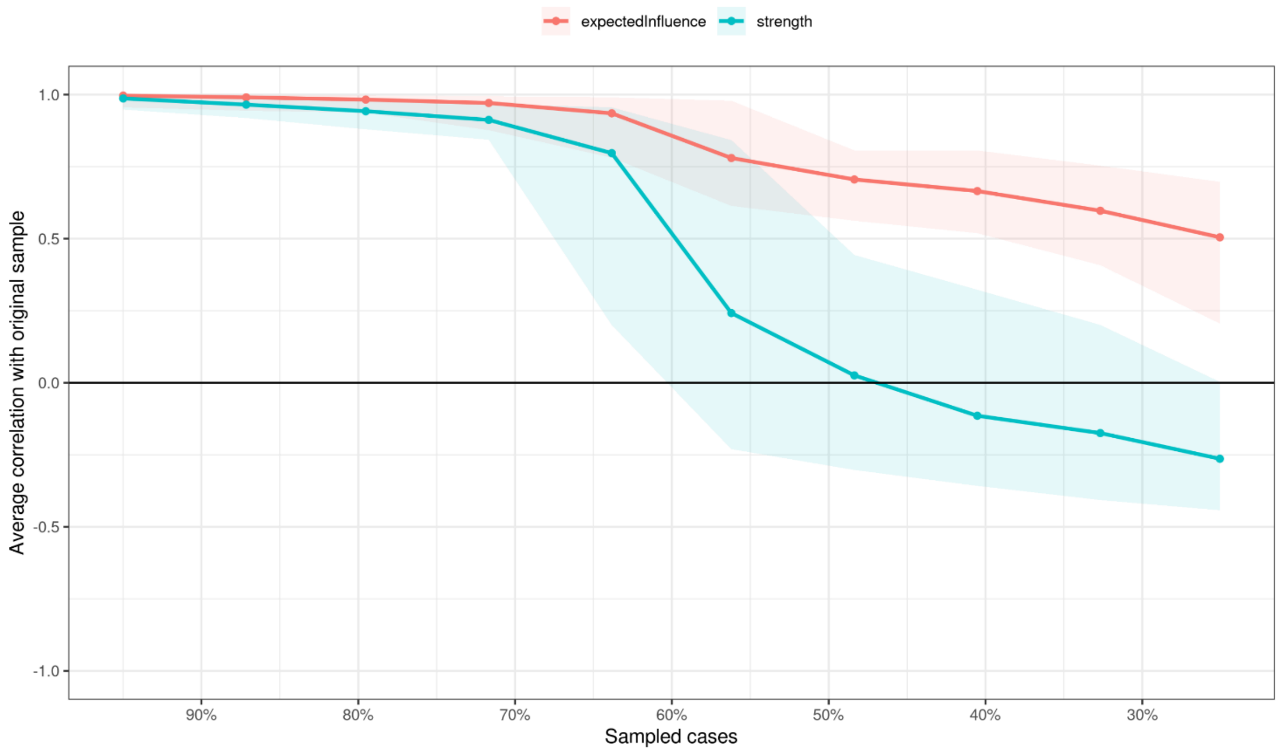

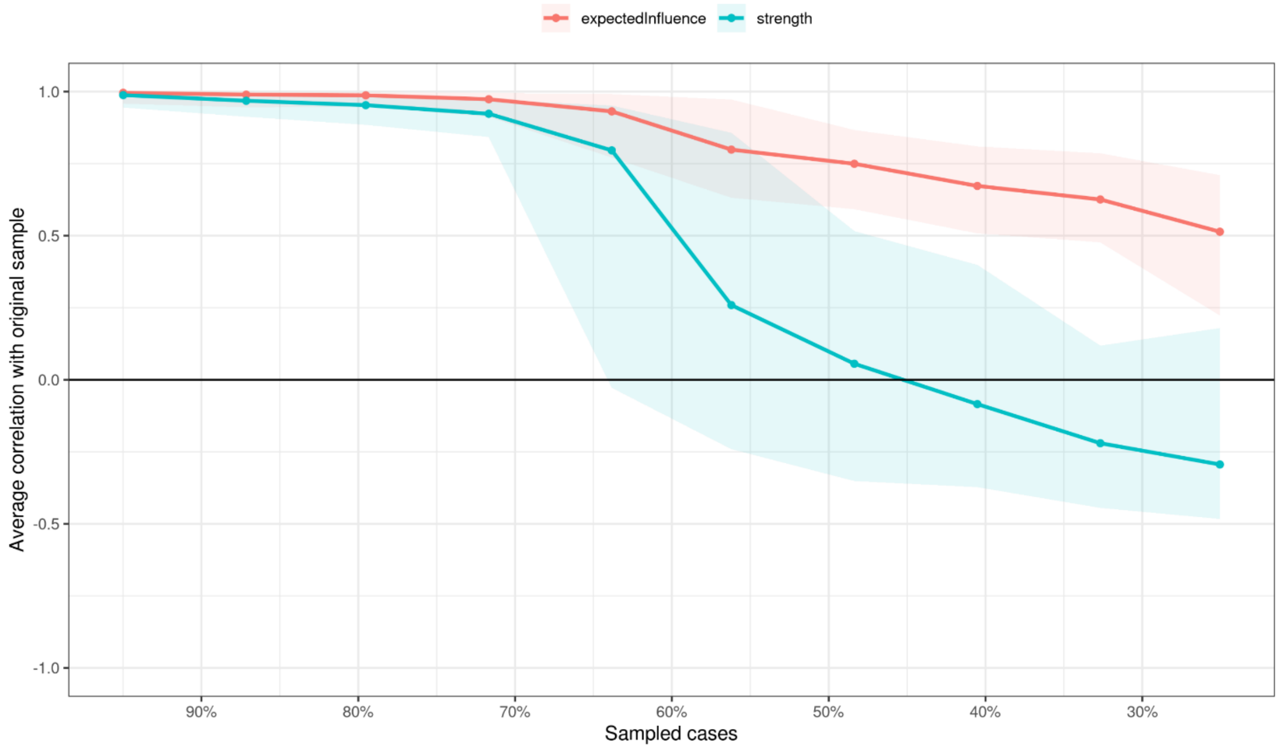

Figure S4 Stability of edges of averaged network A and B in MZ women.

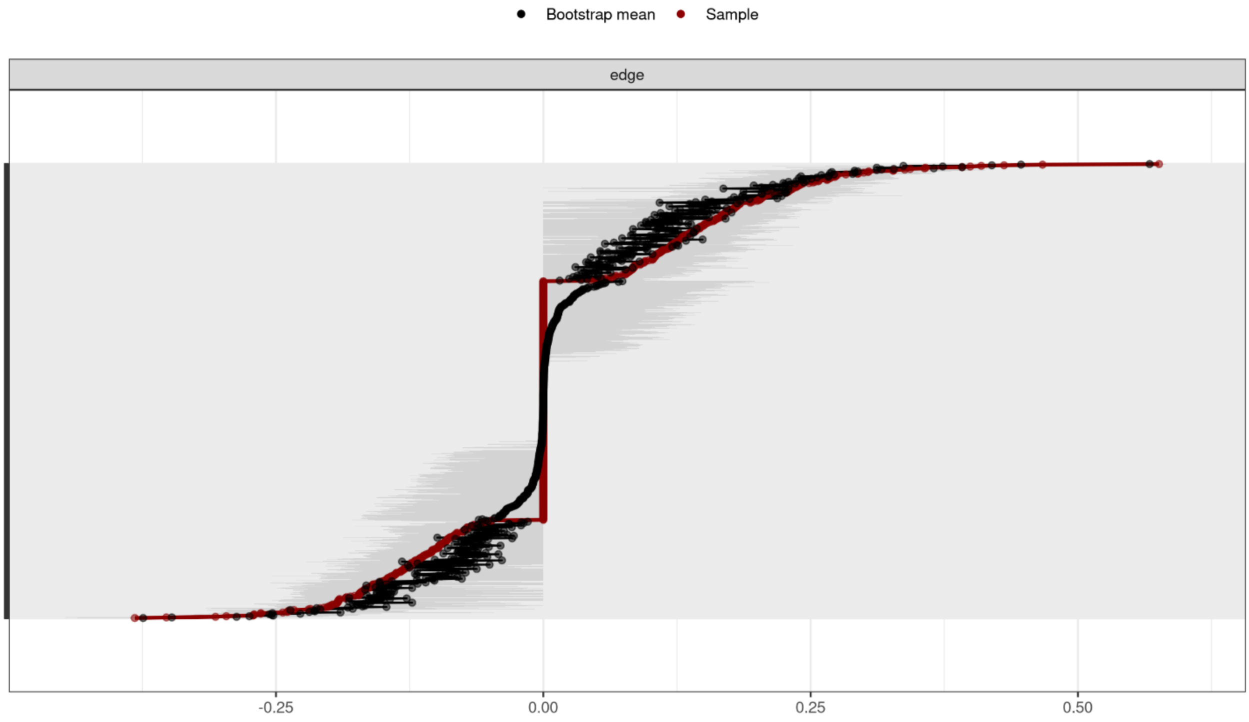

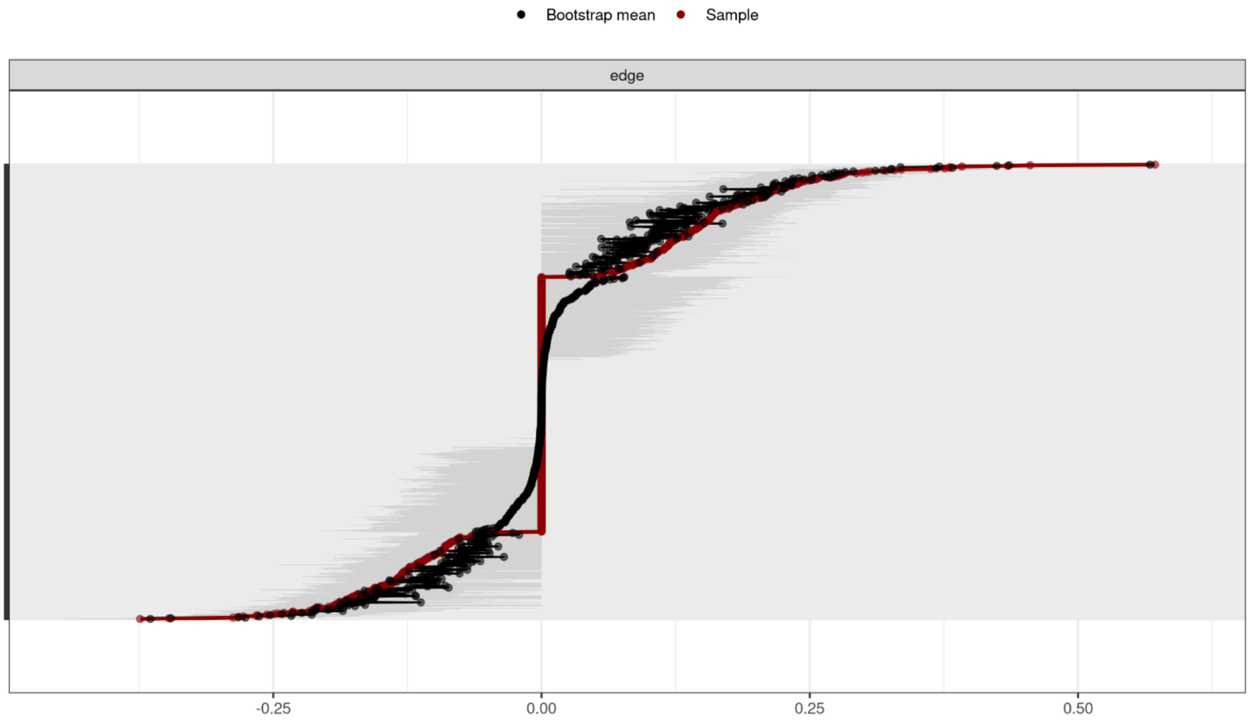

Figure S5 Differences in centrality of averaged network A and B in MZ women.

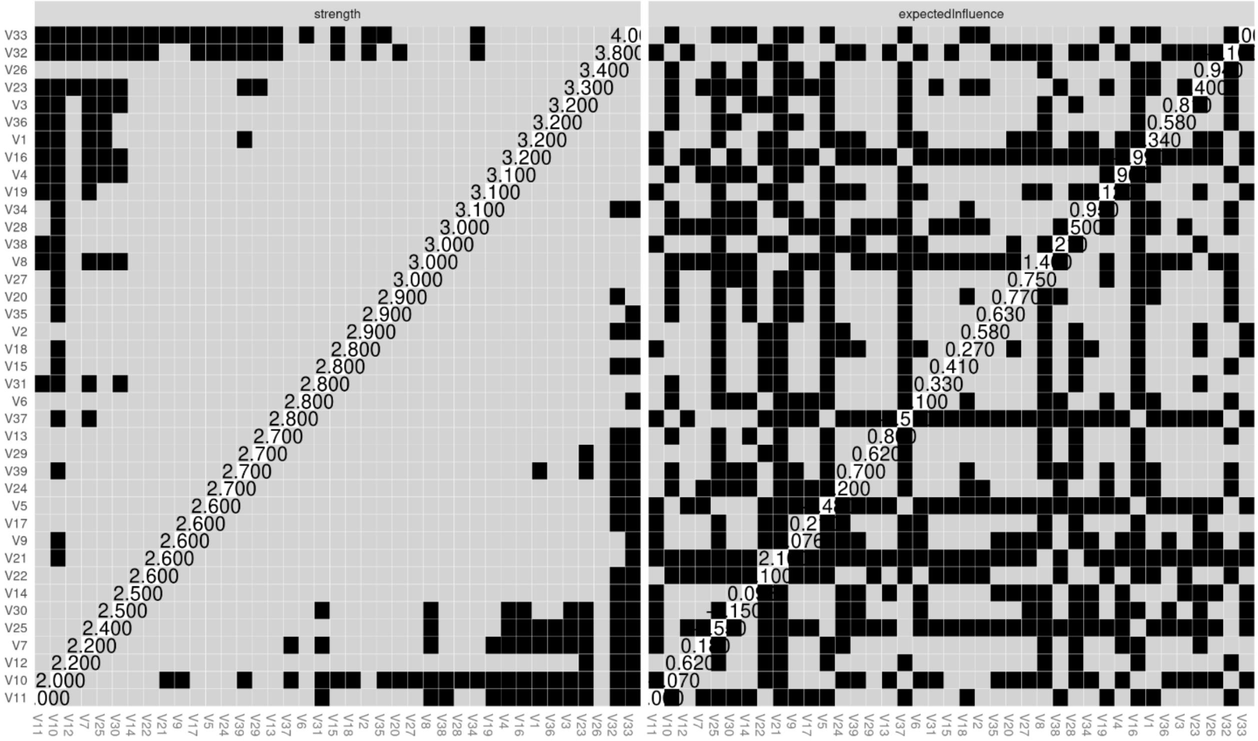

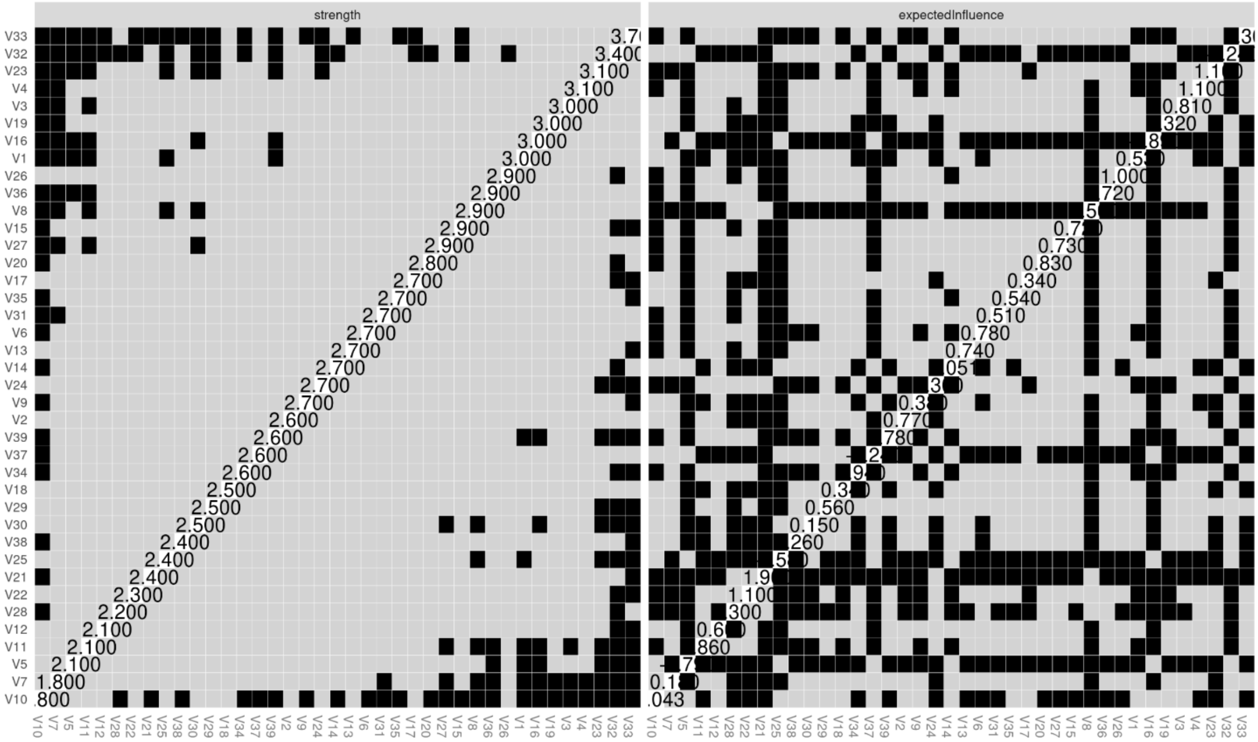

Figure S6 Differences in edges of averaged network A and B in MZ women.

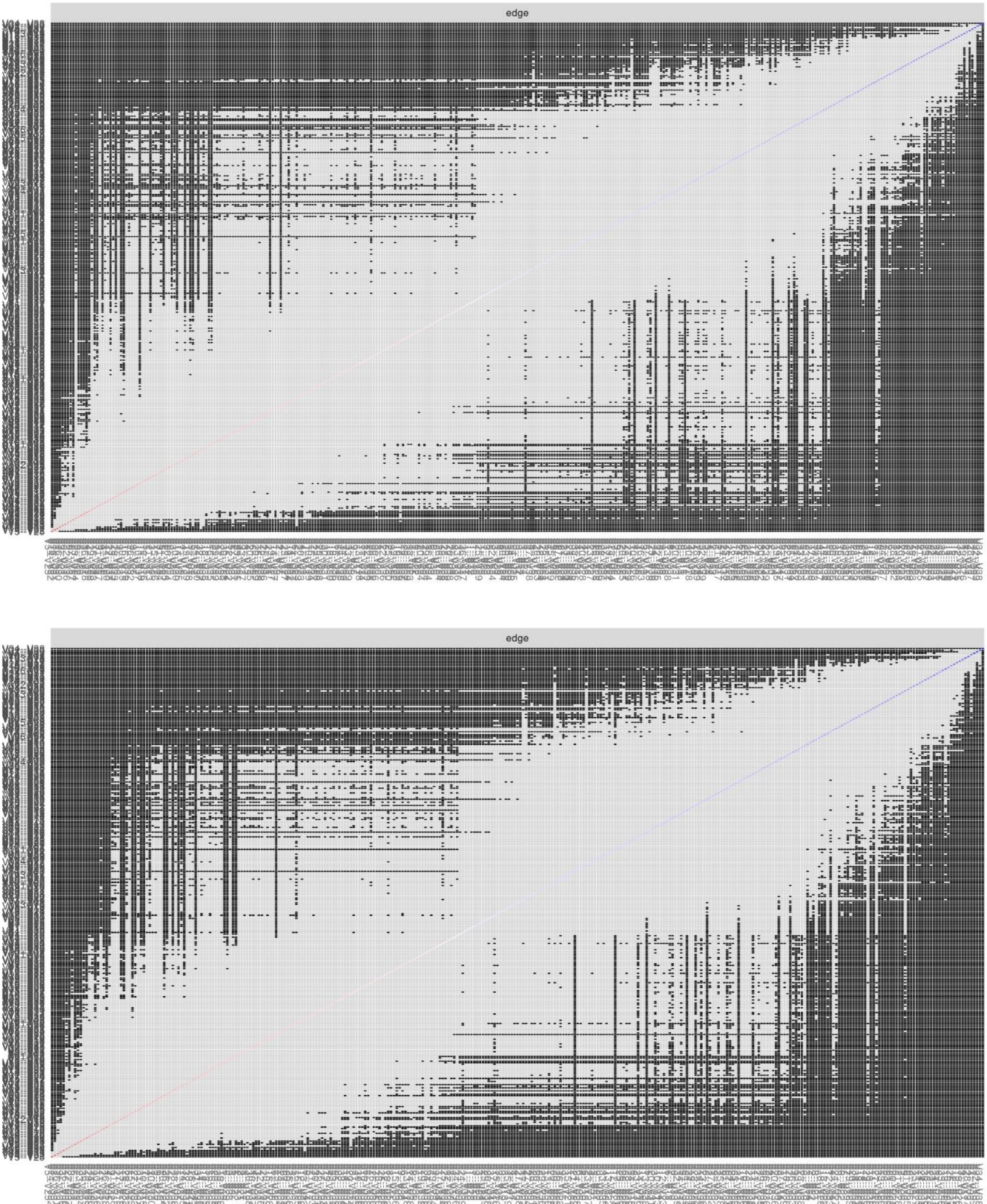

2. Results of DZ women

Figure S7 Averaged network A and B in DZ women after 1000 reassignments.

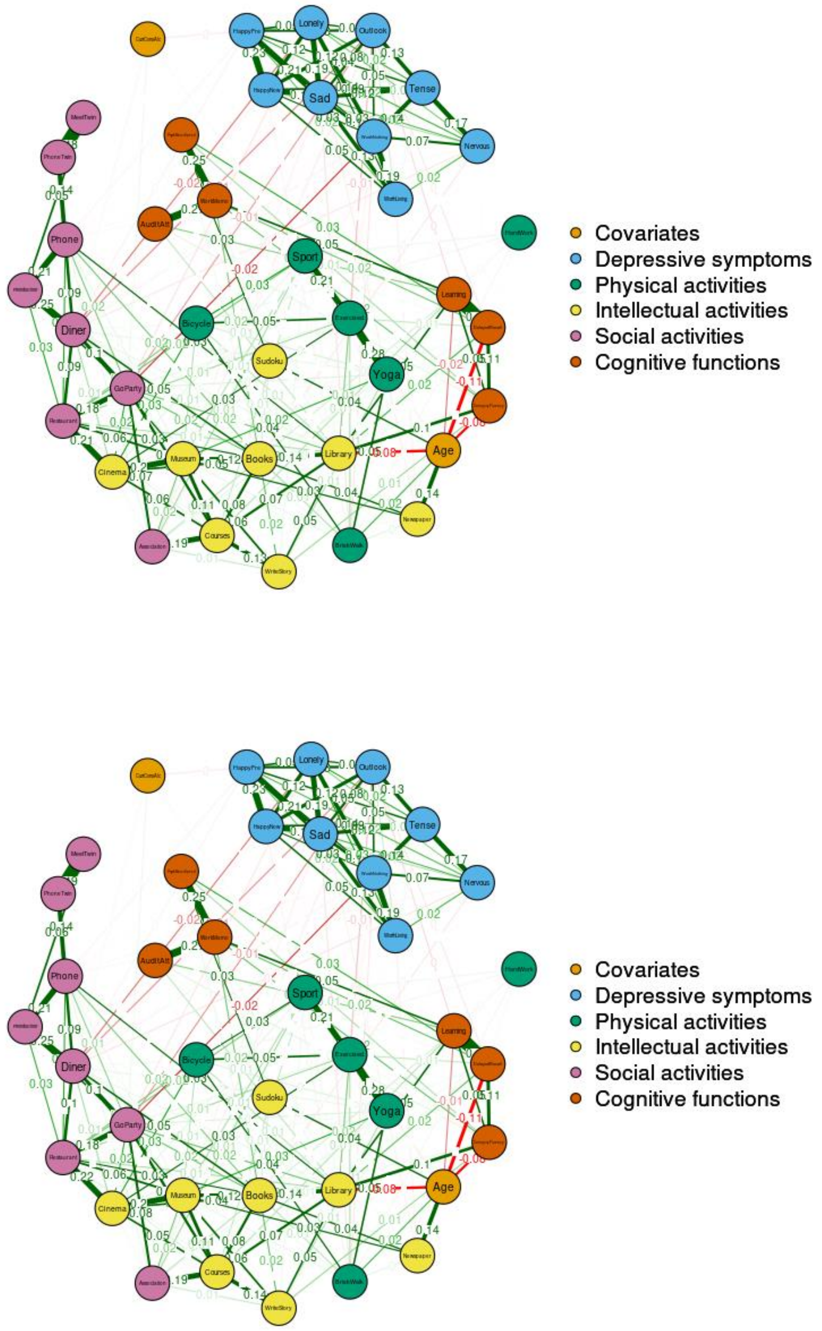

Figure S8 Centrality (Strength and EI) of averaged network A and B in DZ women.

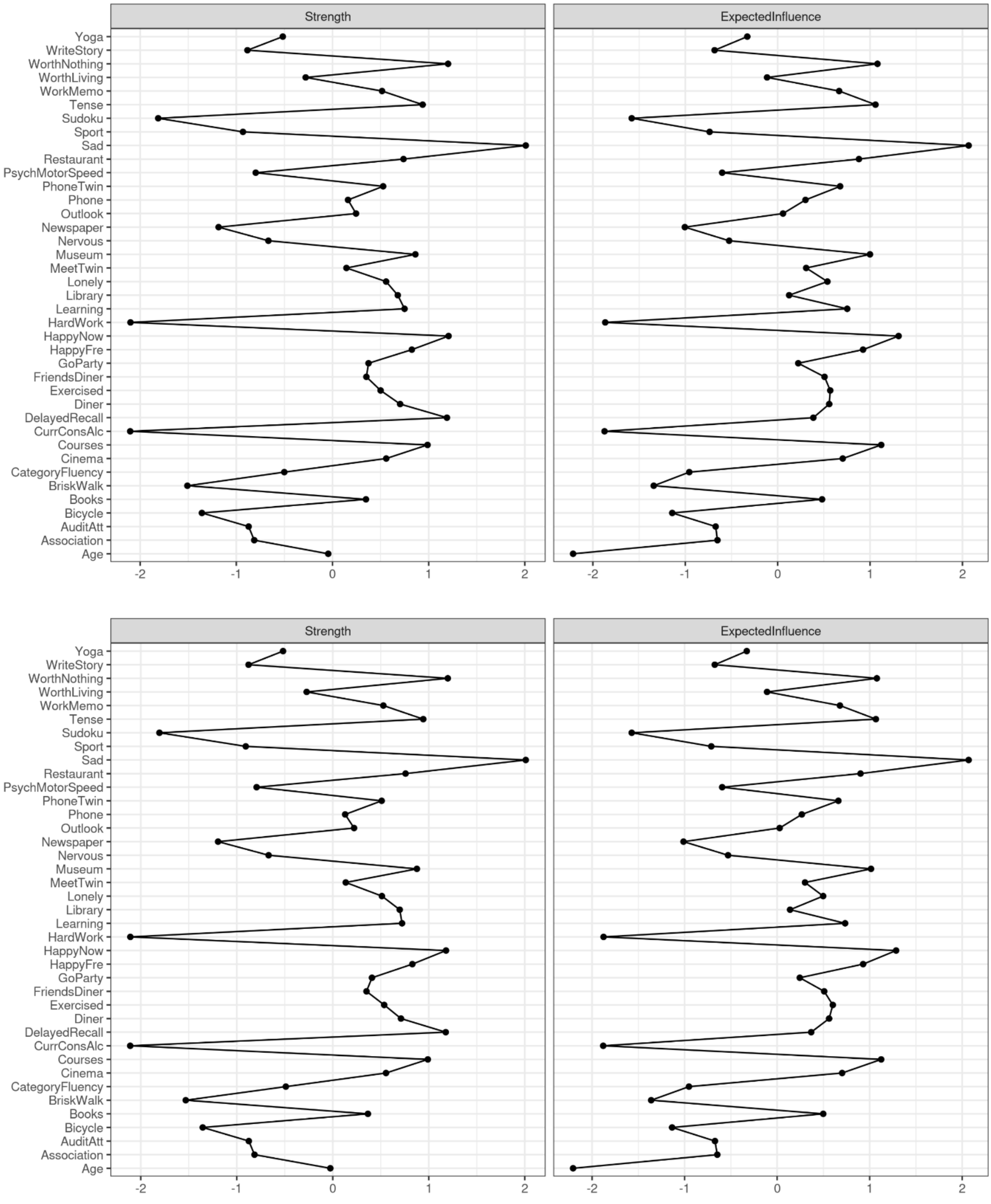

Figure S9 Stability of centrality indices of averaged network A and B in DZ women.

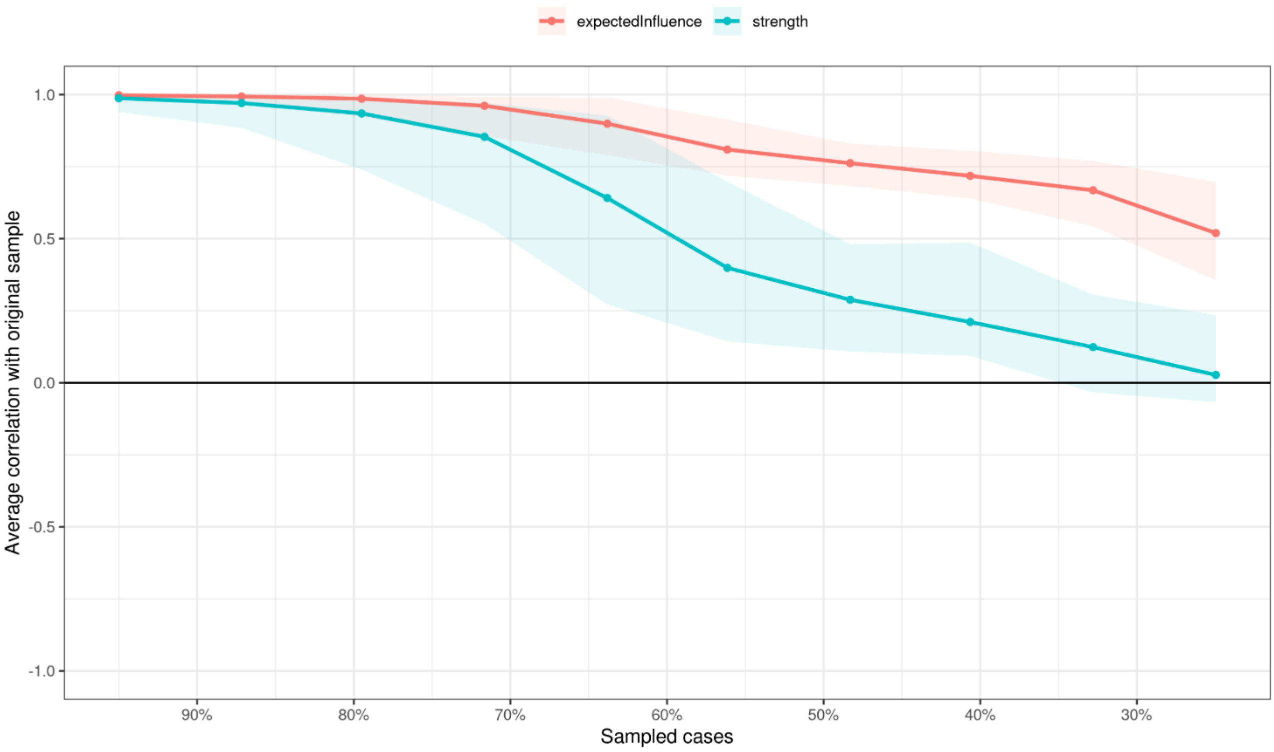

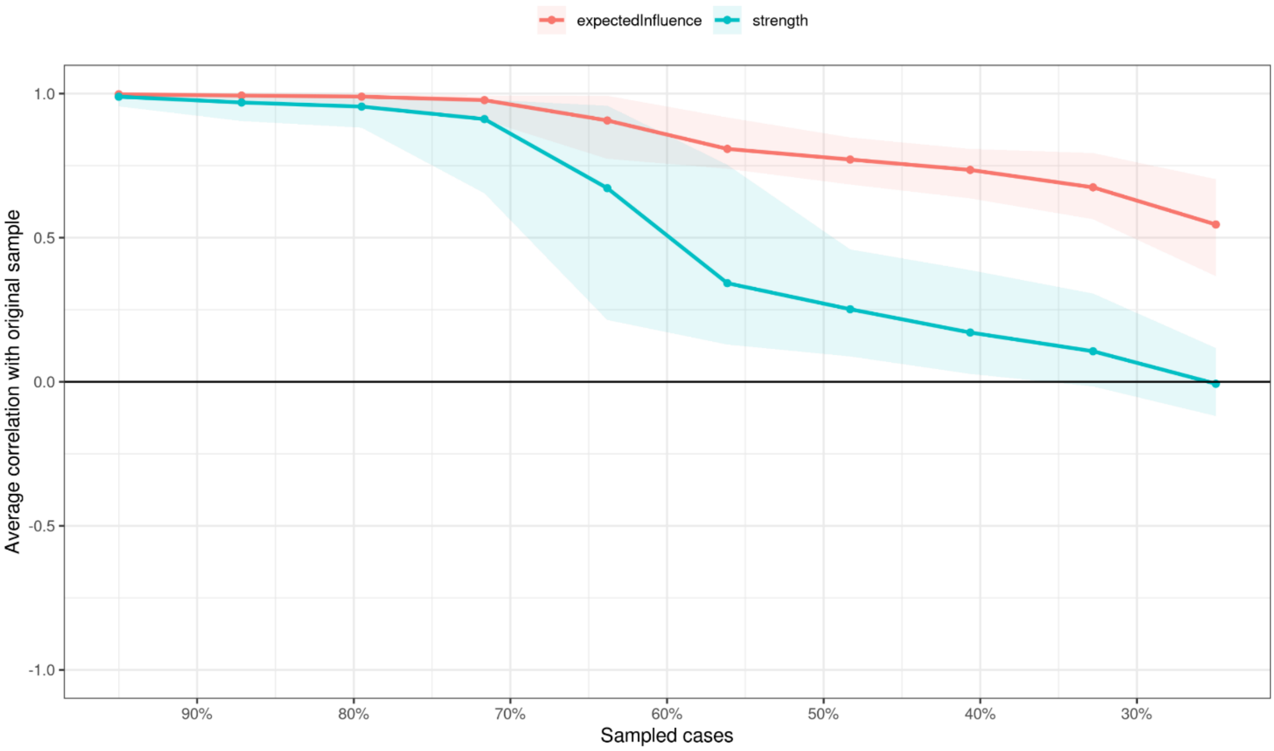

Figure S10 Stability of edges of averaged network A and B in DZ women.

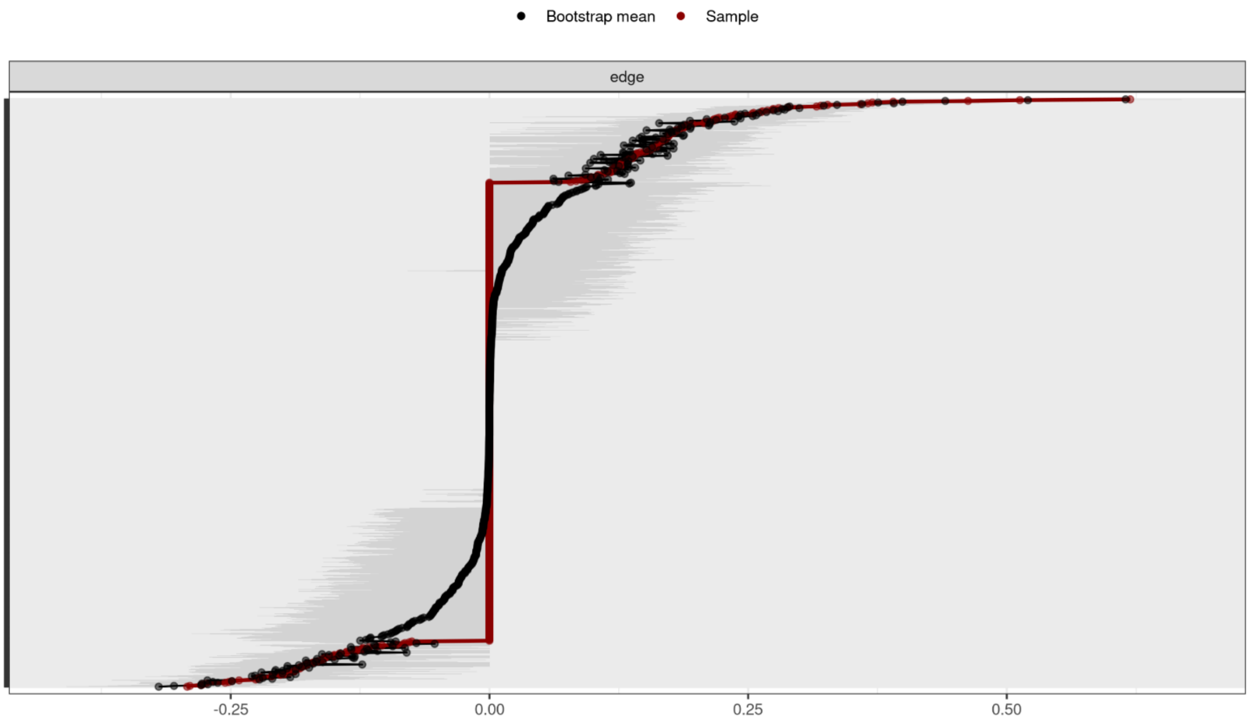

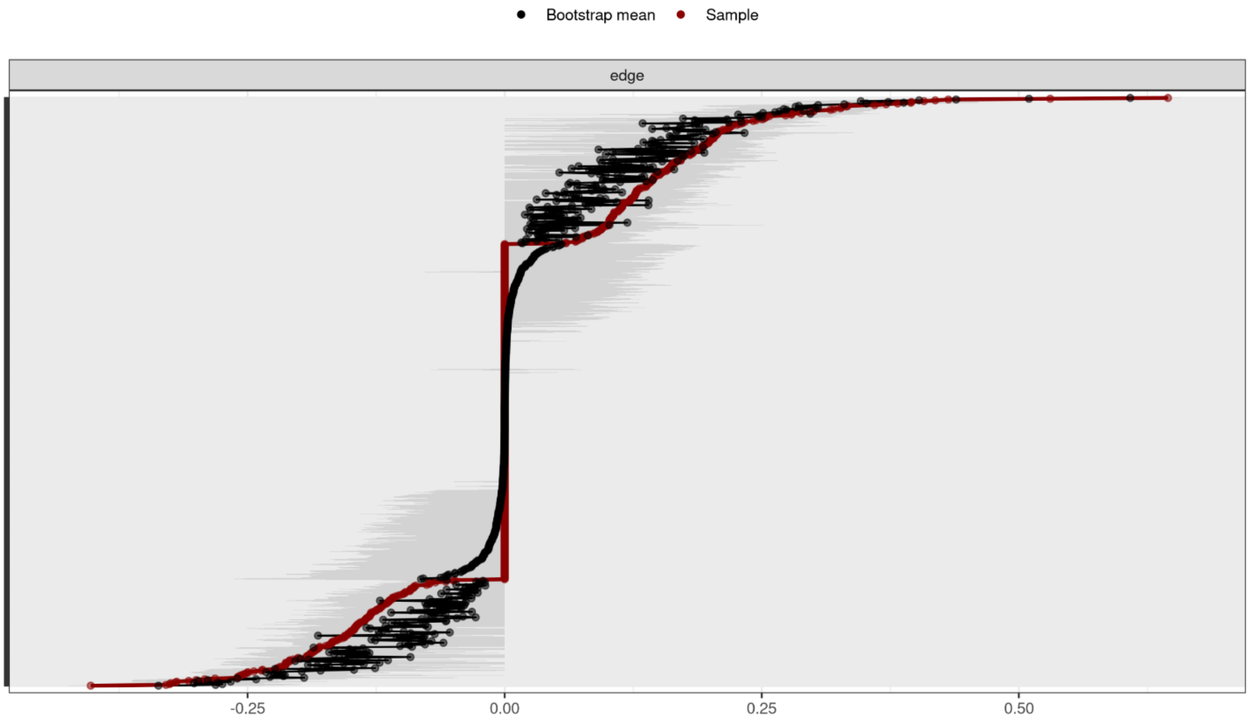

Figure S11 Differences in centrality of averaged network A and B in DZ women.

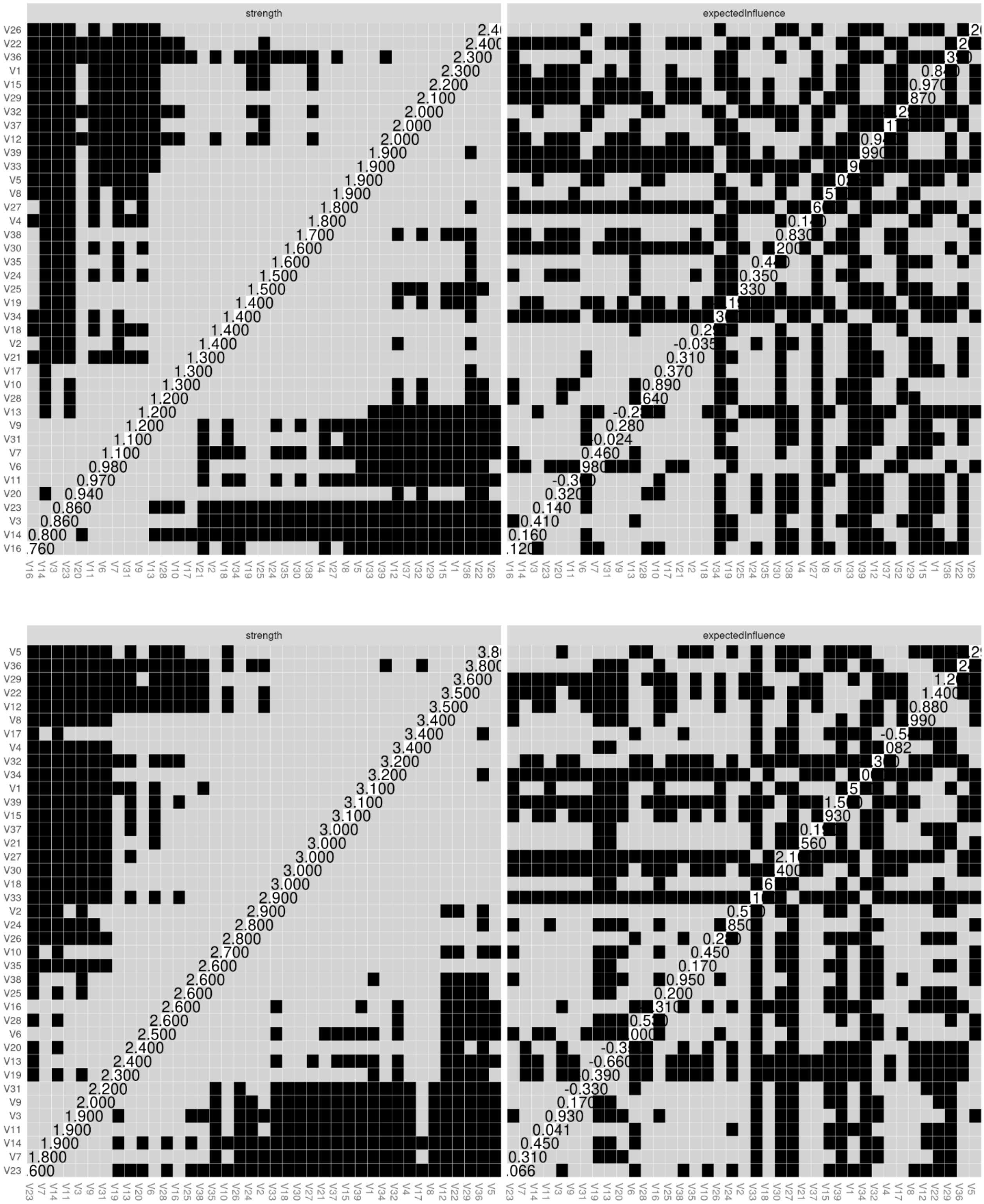

Figure S12 Differences in edges of averaged network A and B in DZ women.

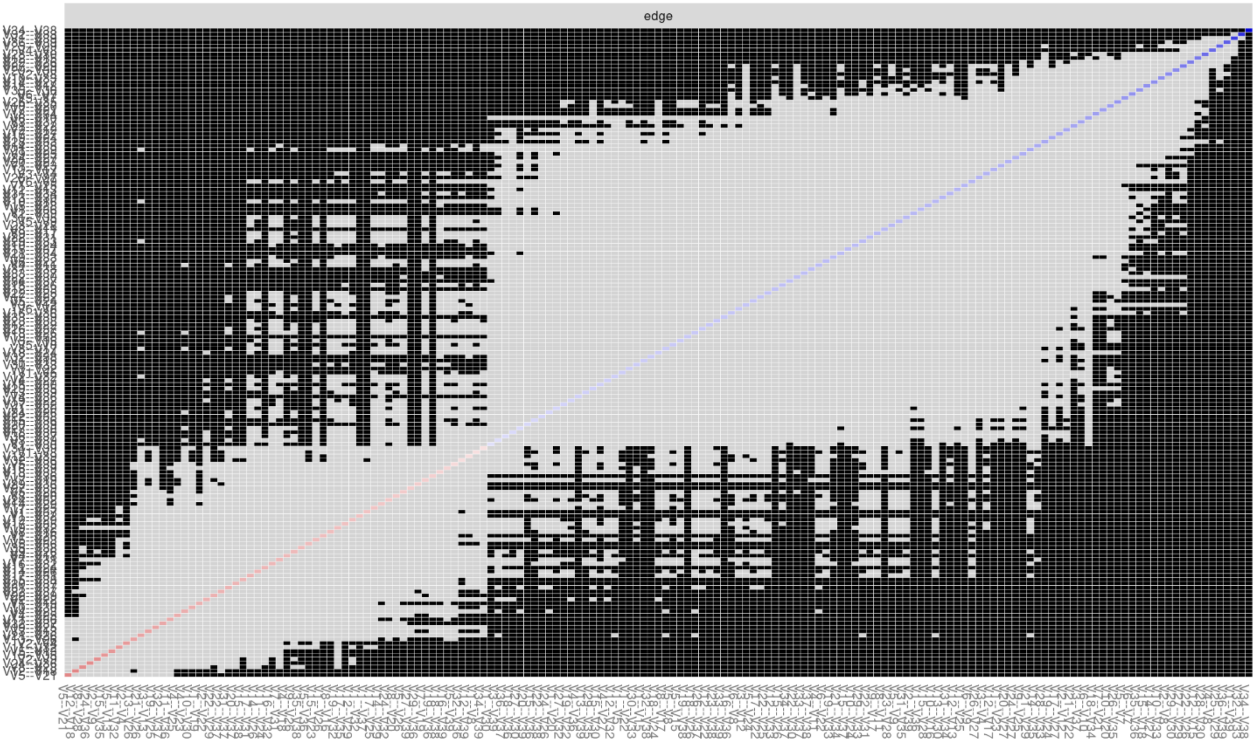

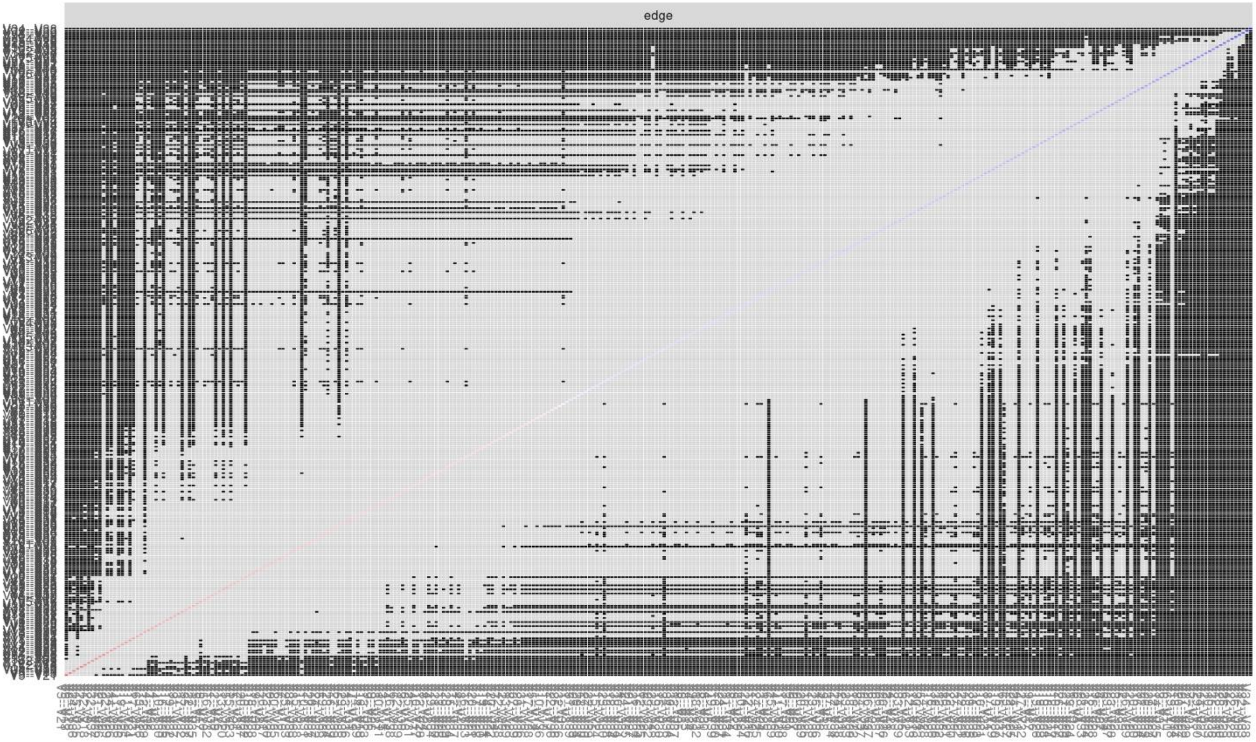

3. Results of MZ men

Figure S13 Averaged network A and B in MZ men after 1000 reassignments.

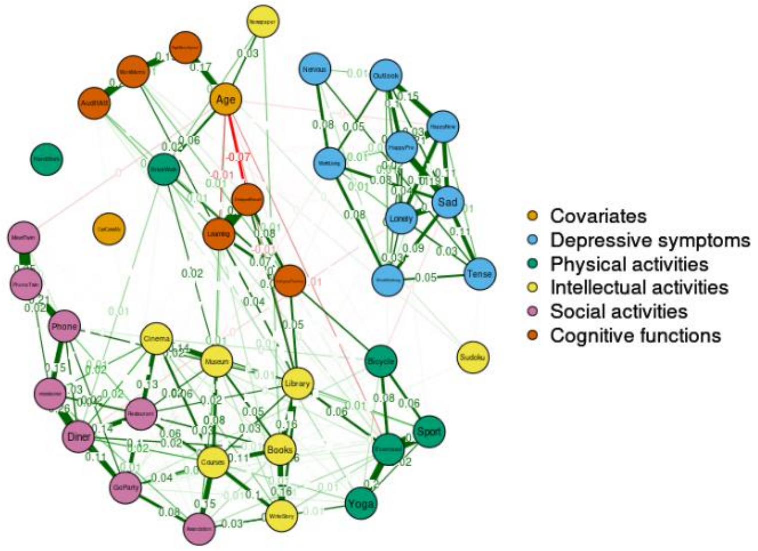

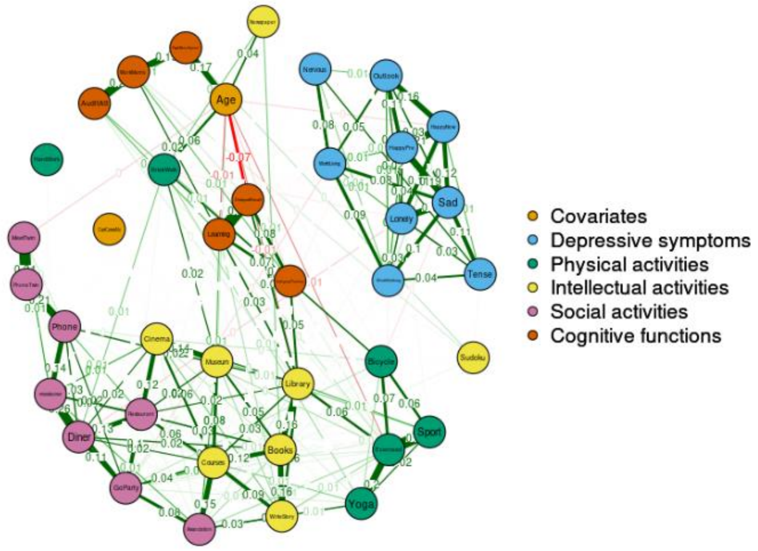

Figure S14 Centrality (Strength and EI) of averaged network A and B in MZ men.

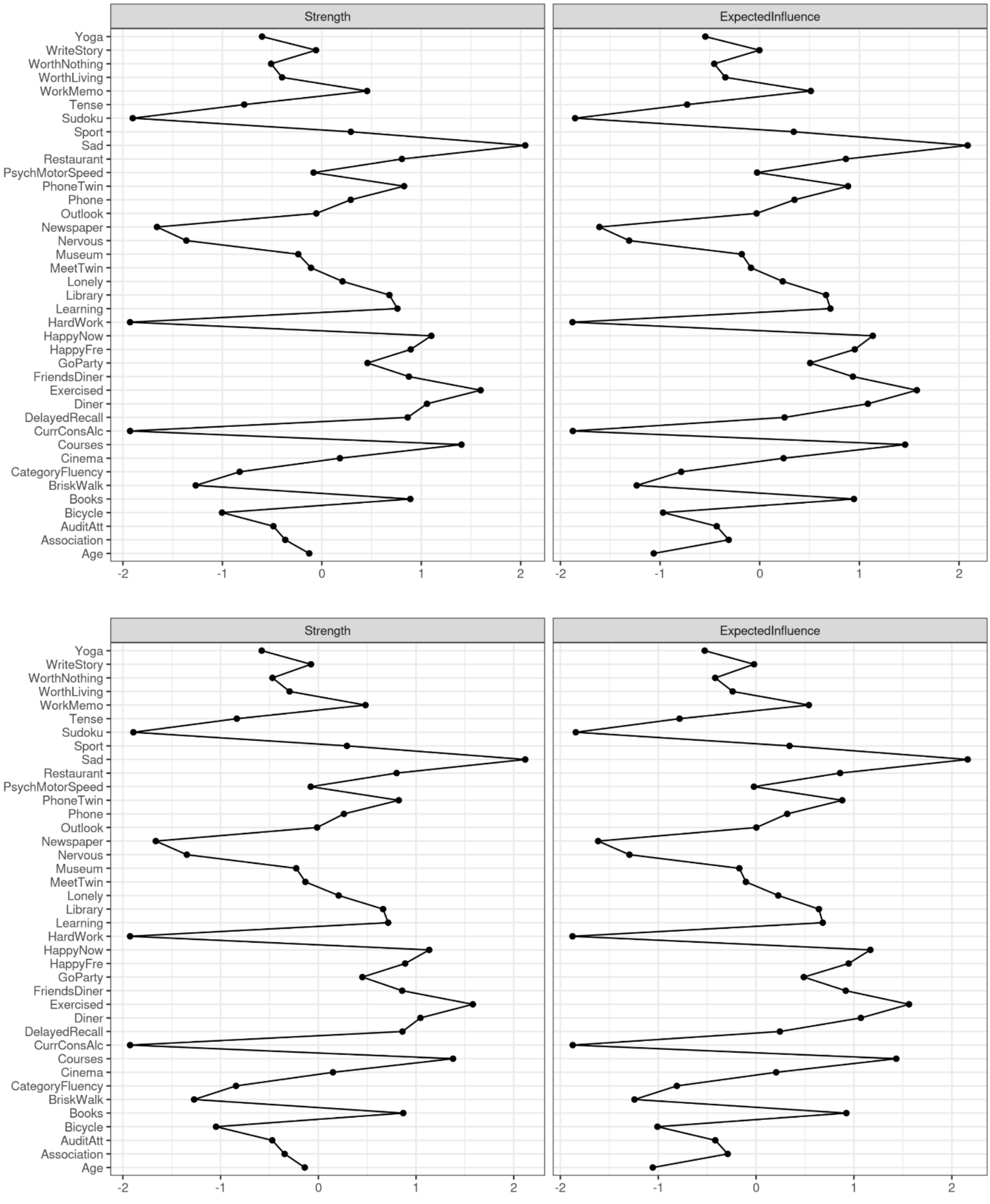

Figure S15 Stability of centrality indices of averaged network A and B in MZ men.

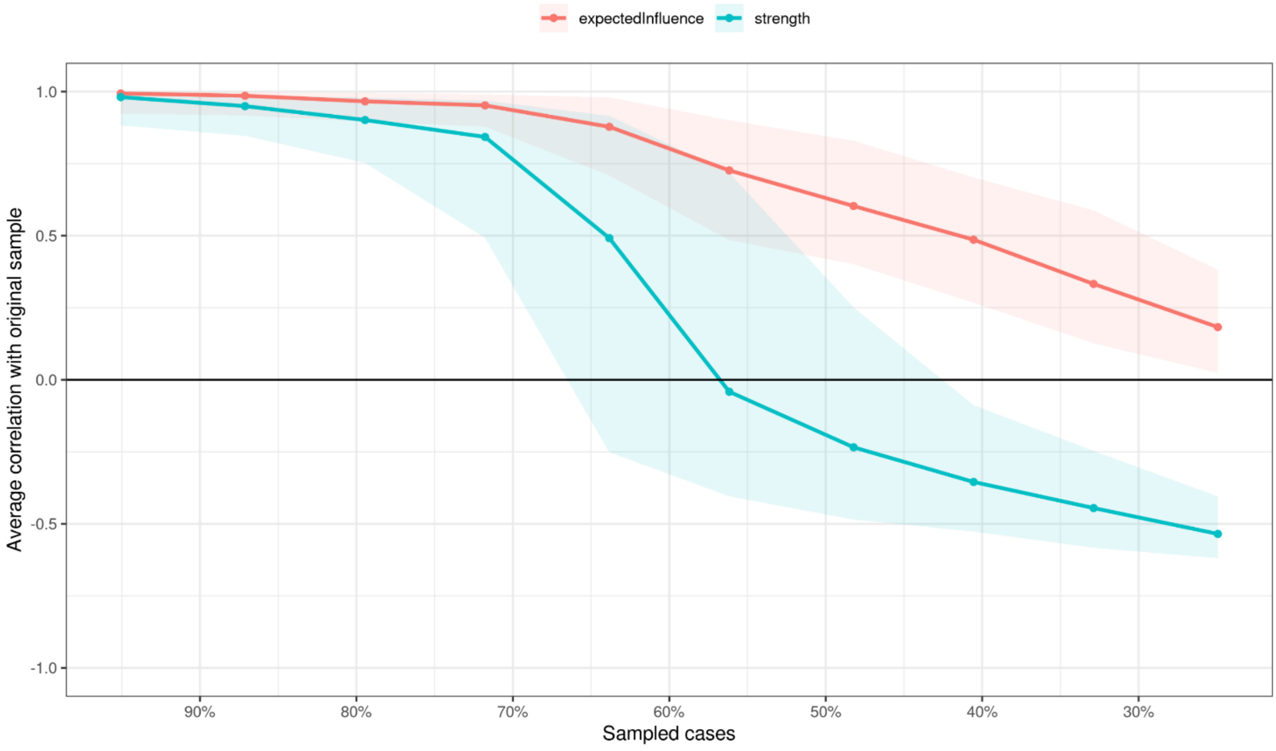

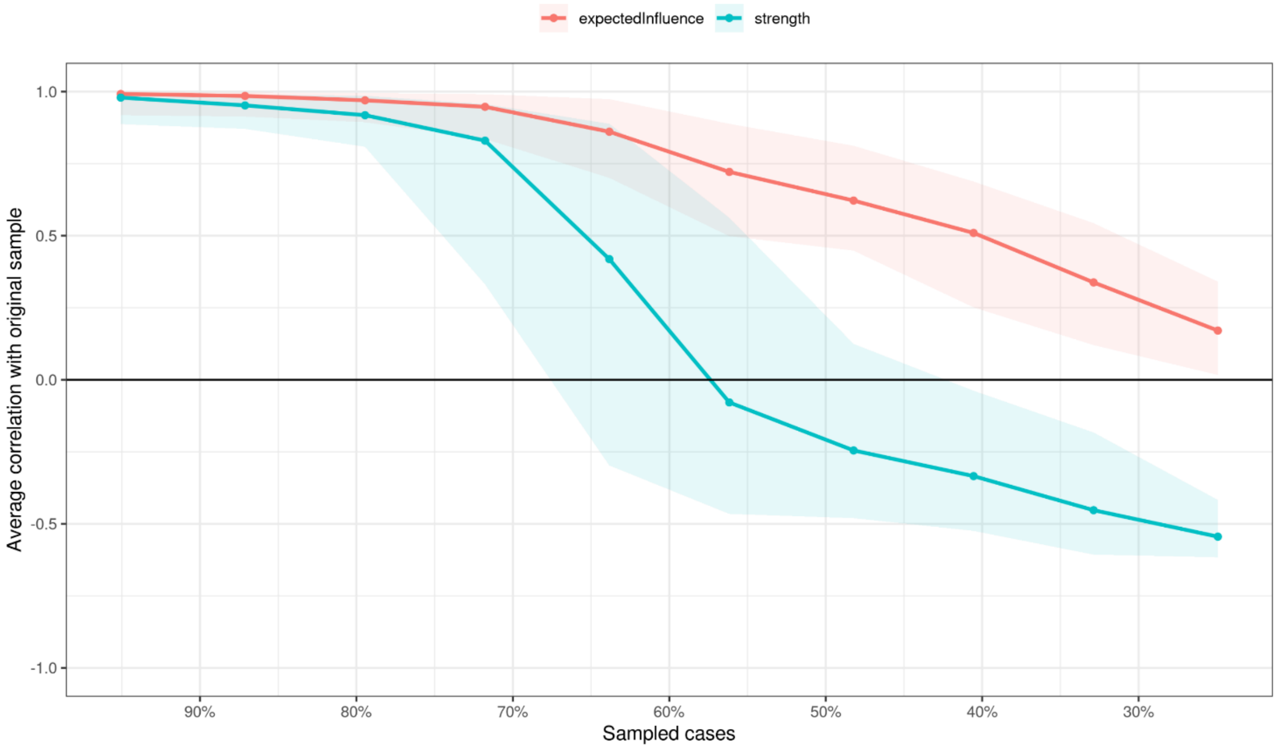

Figure S16 Stability of edges of averaged network A and B in MZ men.

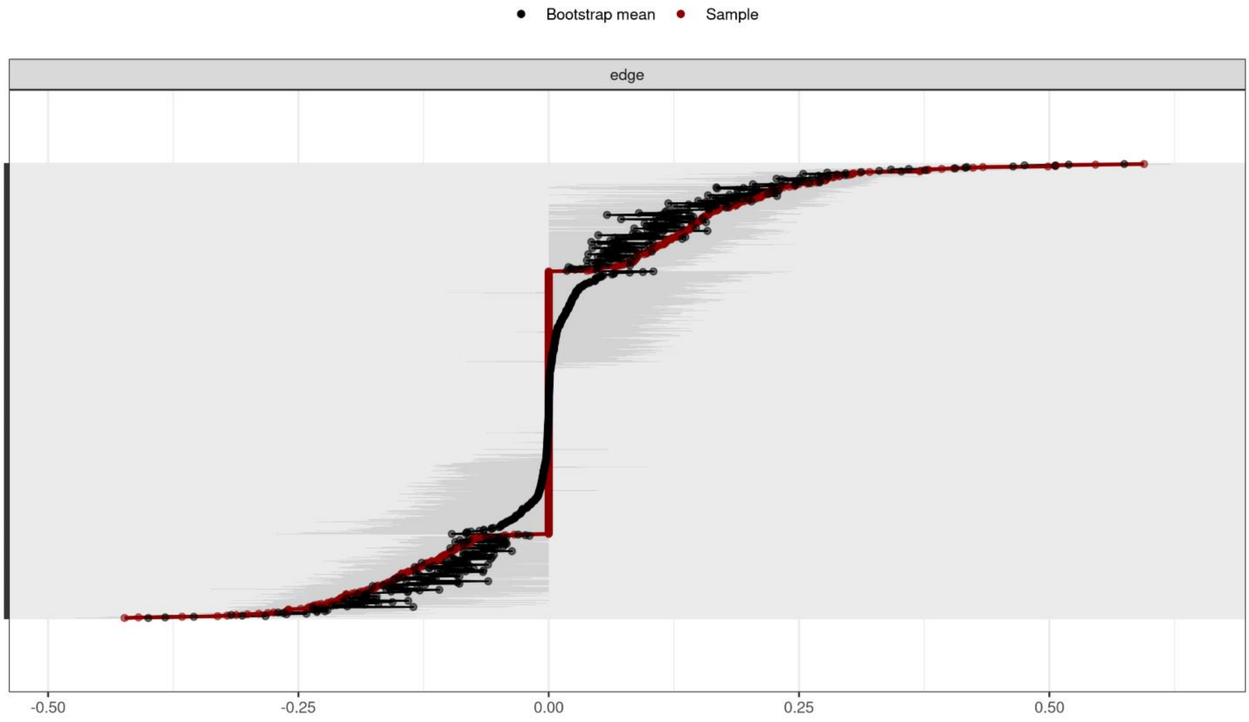

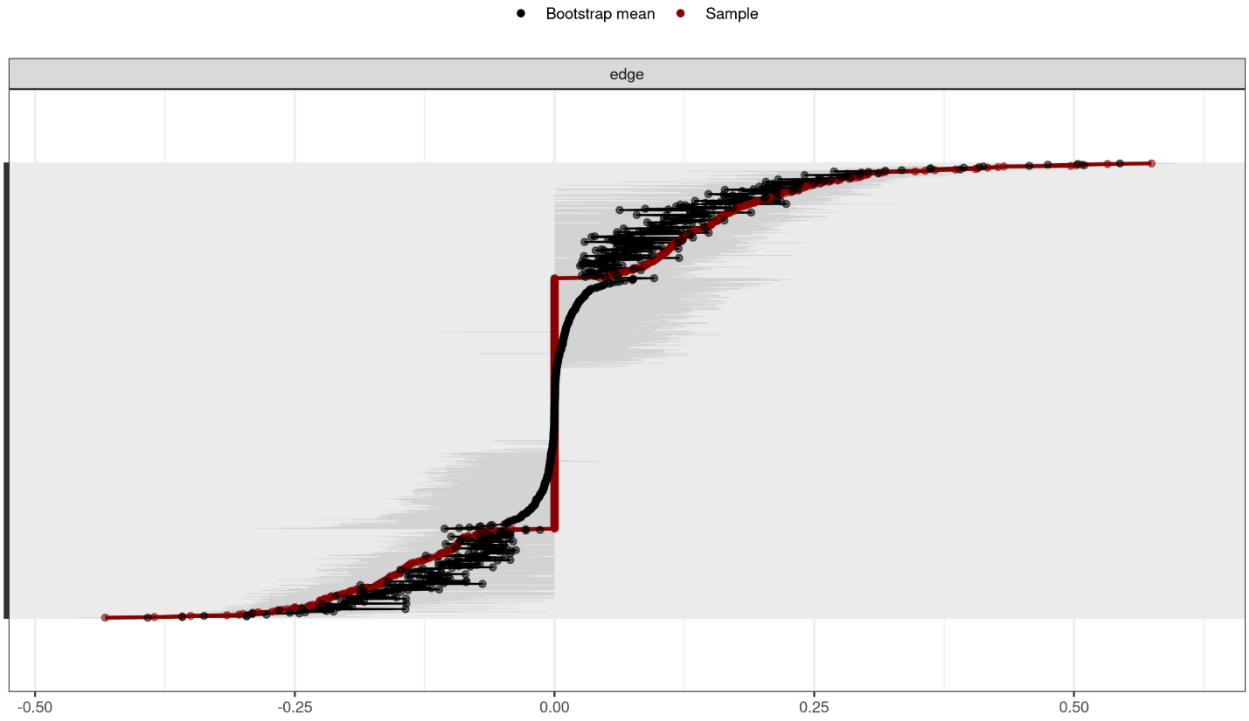

Figure S17 Differences in centrality of averaged network A and B in MZ men.

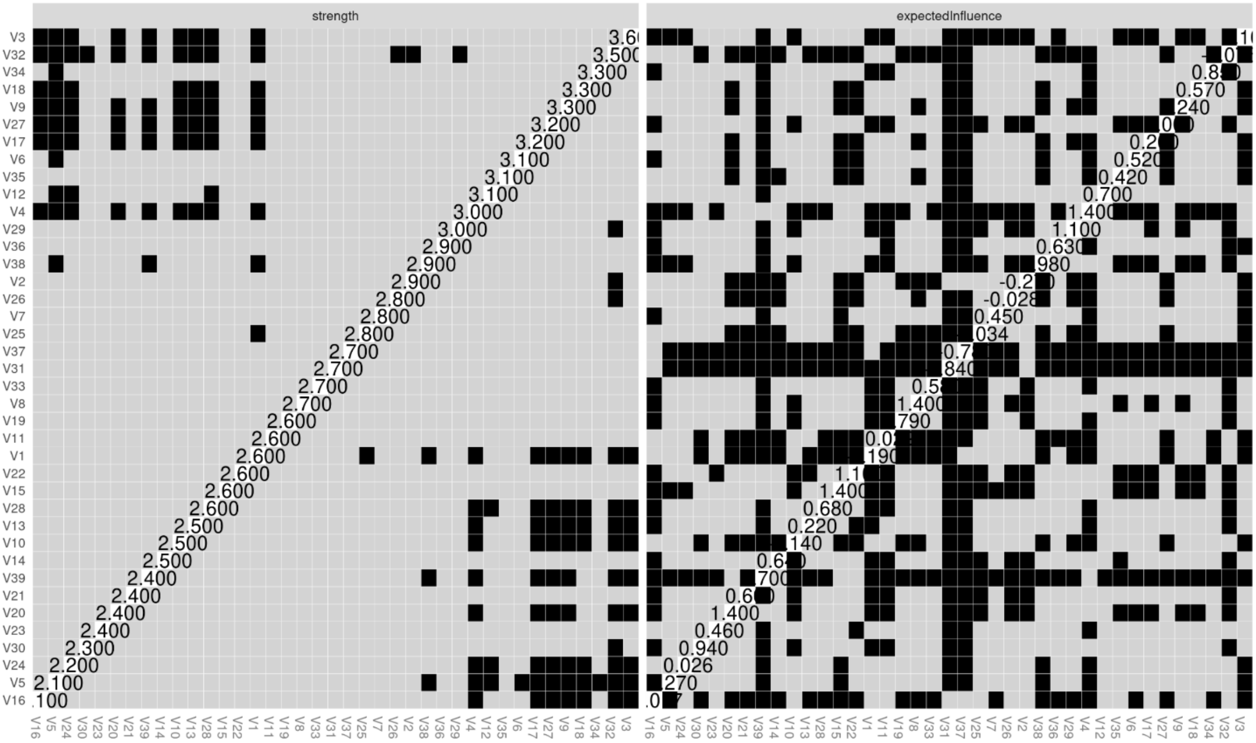

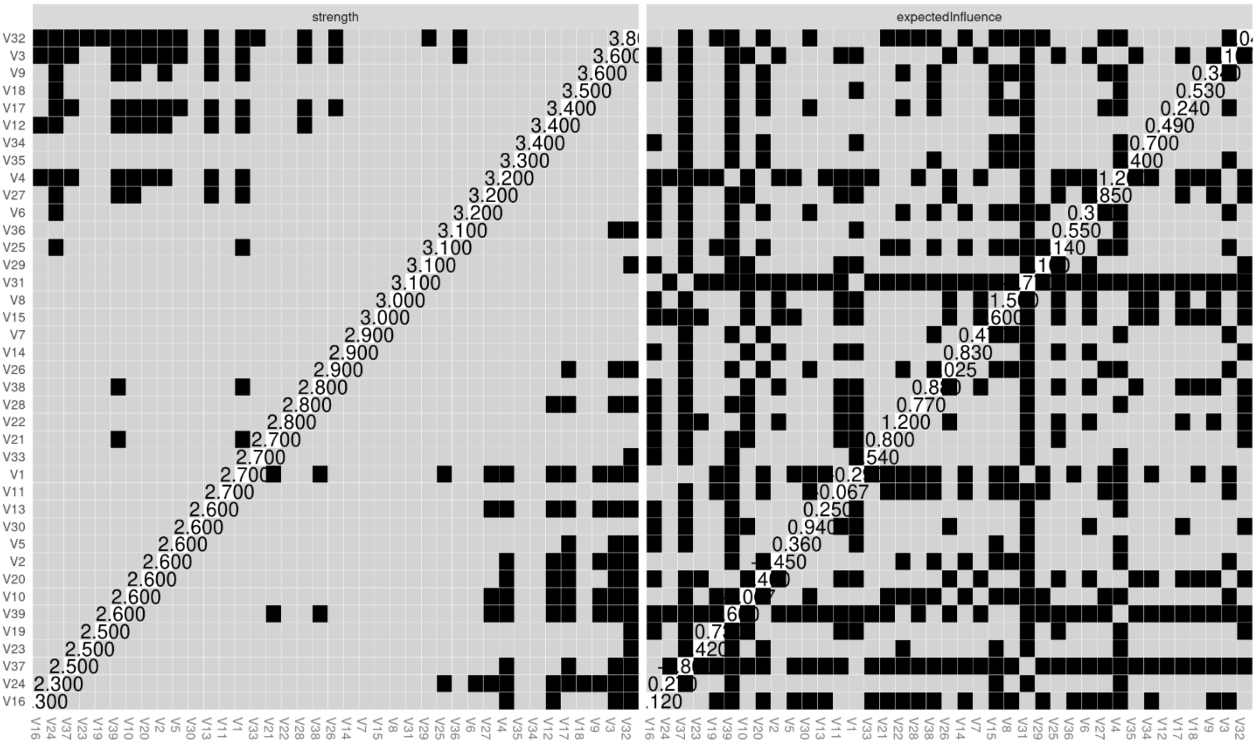

Figure S18 Differences in edges of averaged network A and B in MZ men.

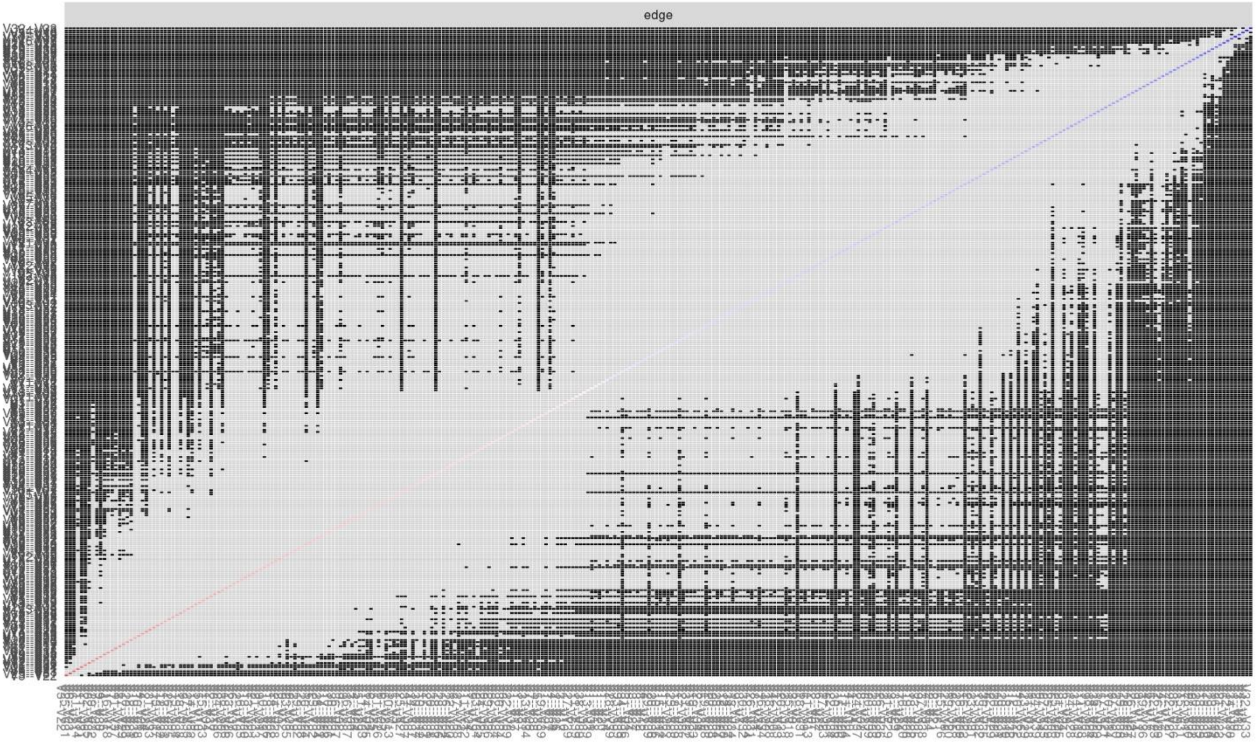
edge

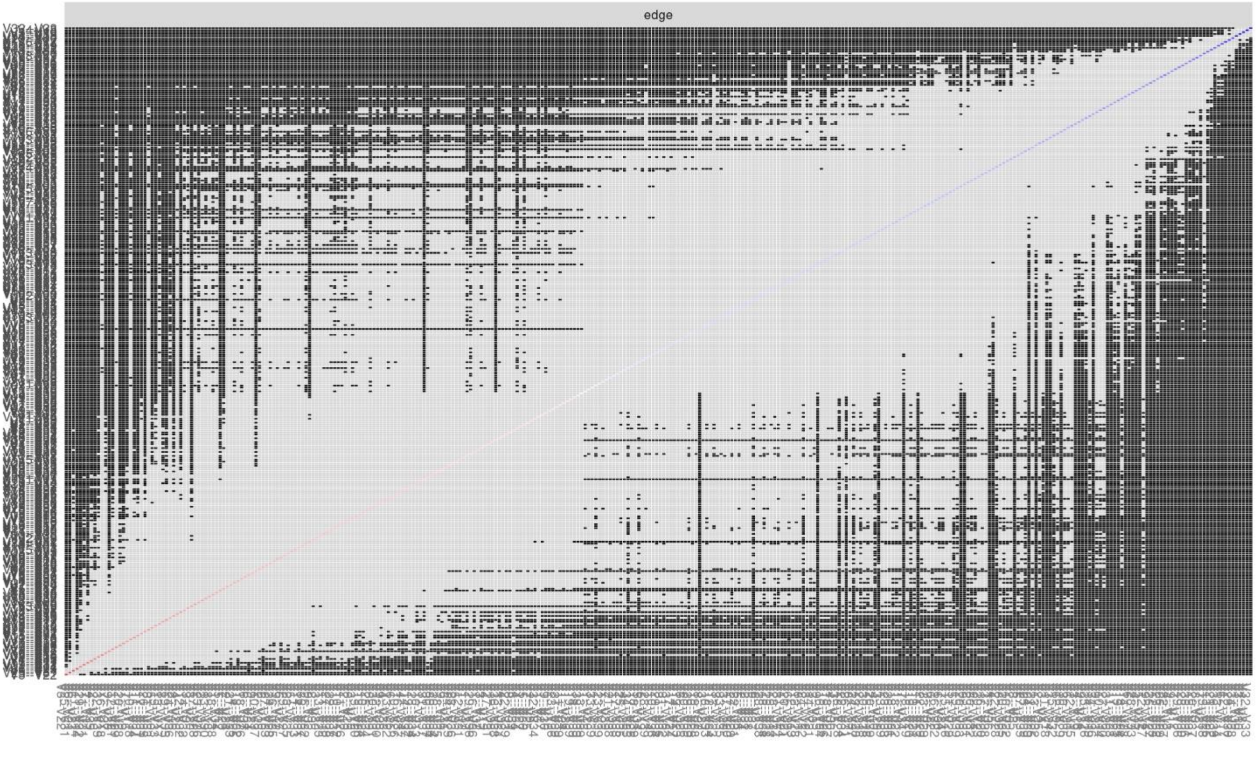
edge

4. Results of DZ men

Figure S19 Averaged network A and B in DZ men after 1000 reassignments.

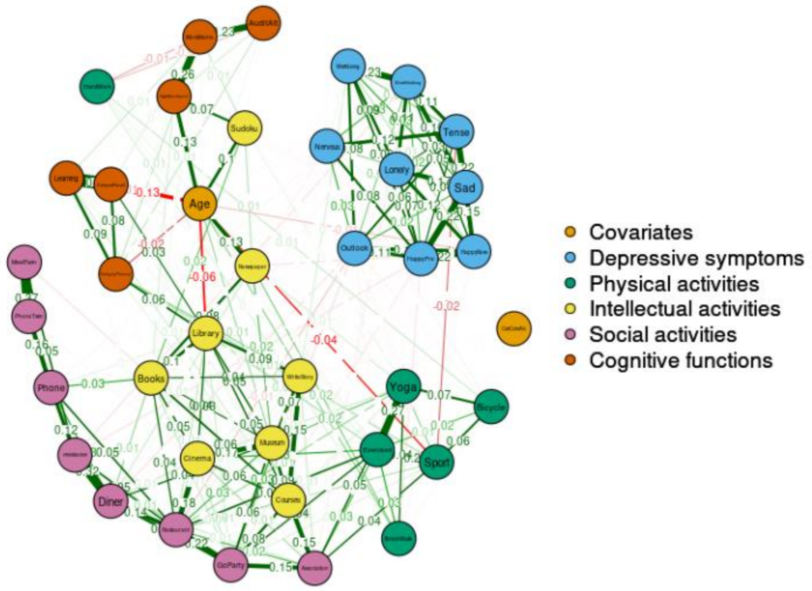

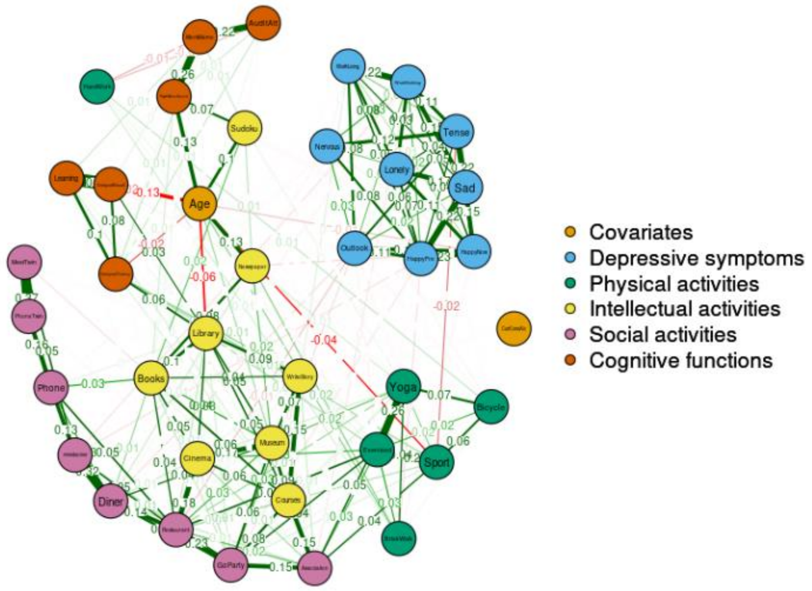

Figure S20 Centrality (Strength and EI) of averaged network A and B in DZ men.

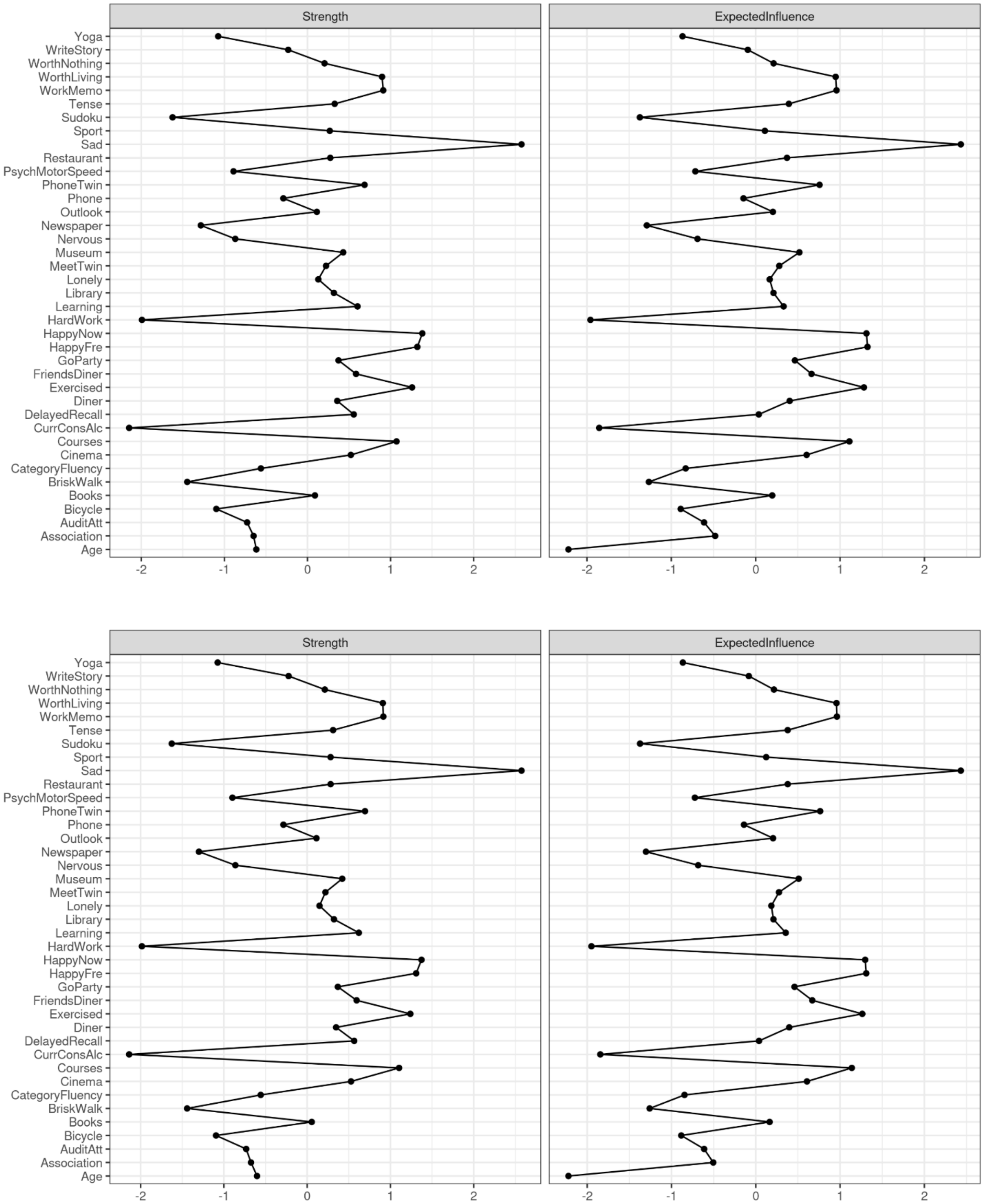

Figure S21 Stability of centrality indices of averaged network A and B in DZ men.

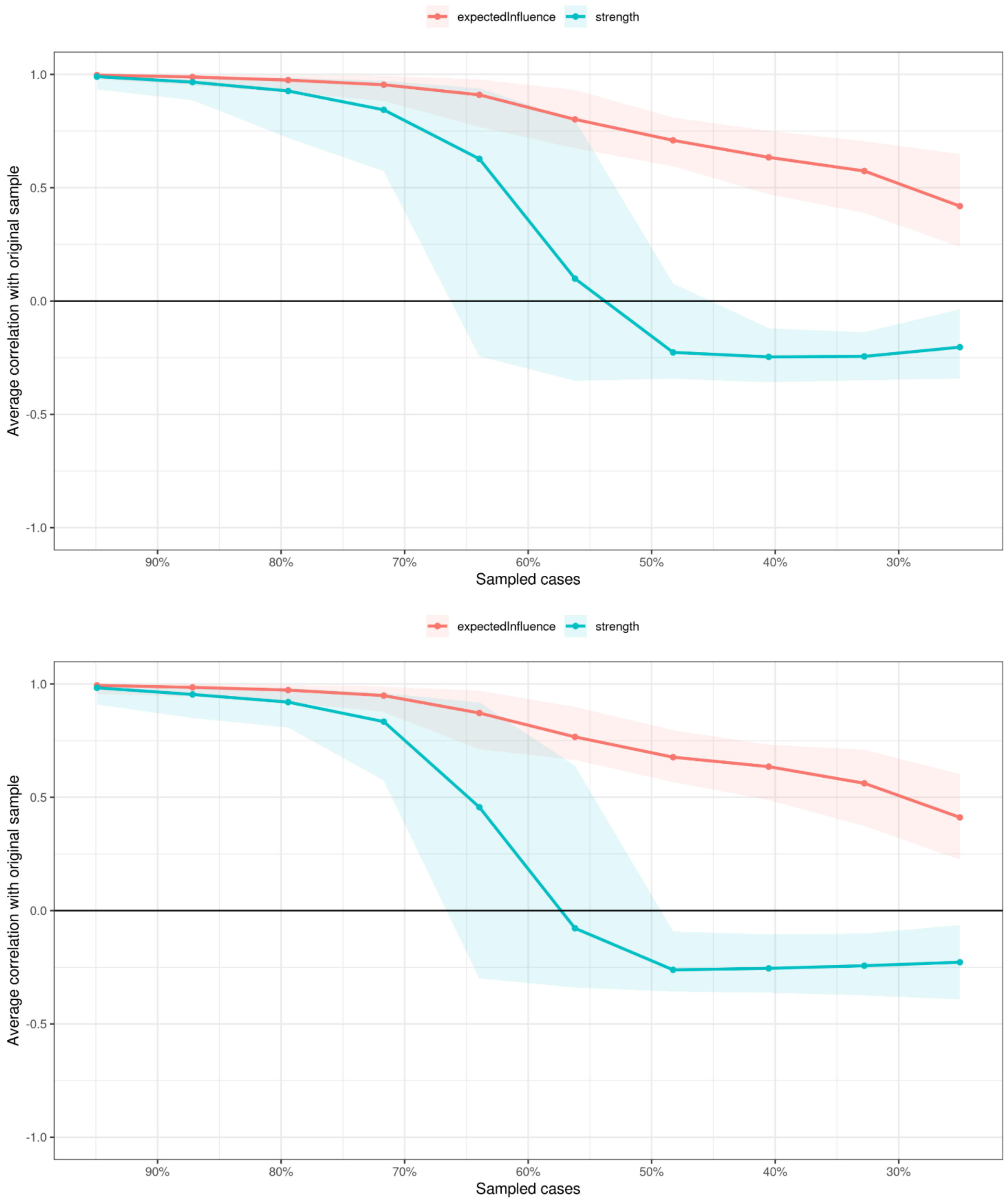

Figure S22 Stability of edges of averaged network A and B in DZ men.

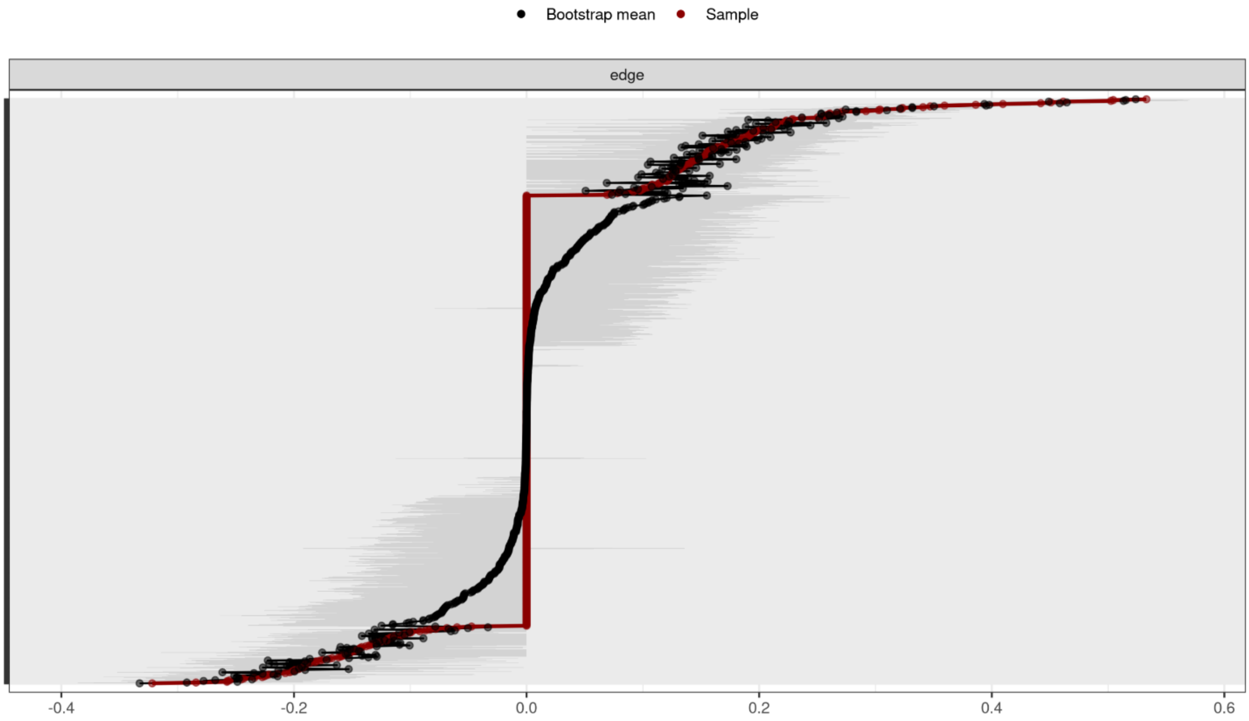

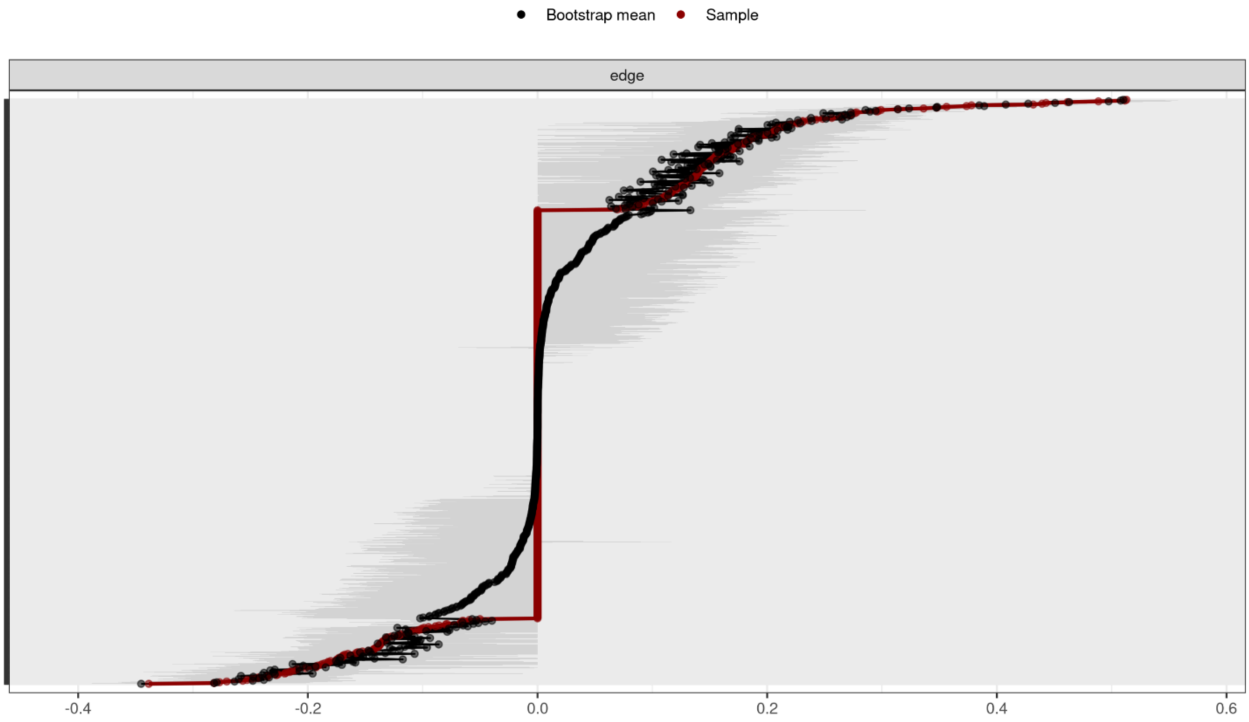

Figure S23 Differences in centrality of averaged network A and B in DZ men.

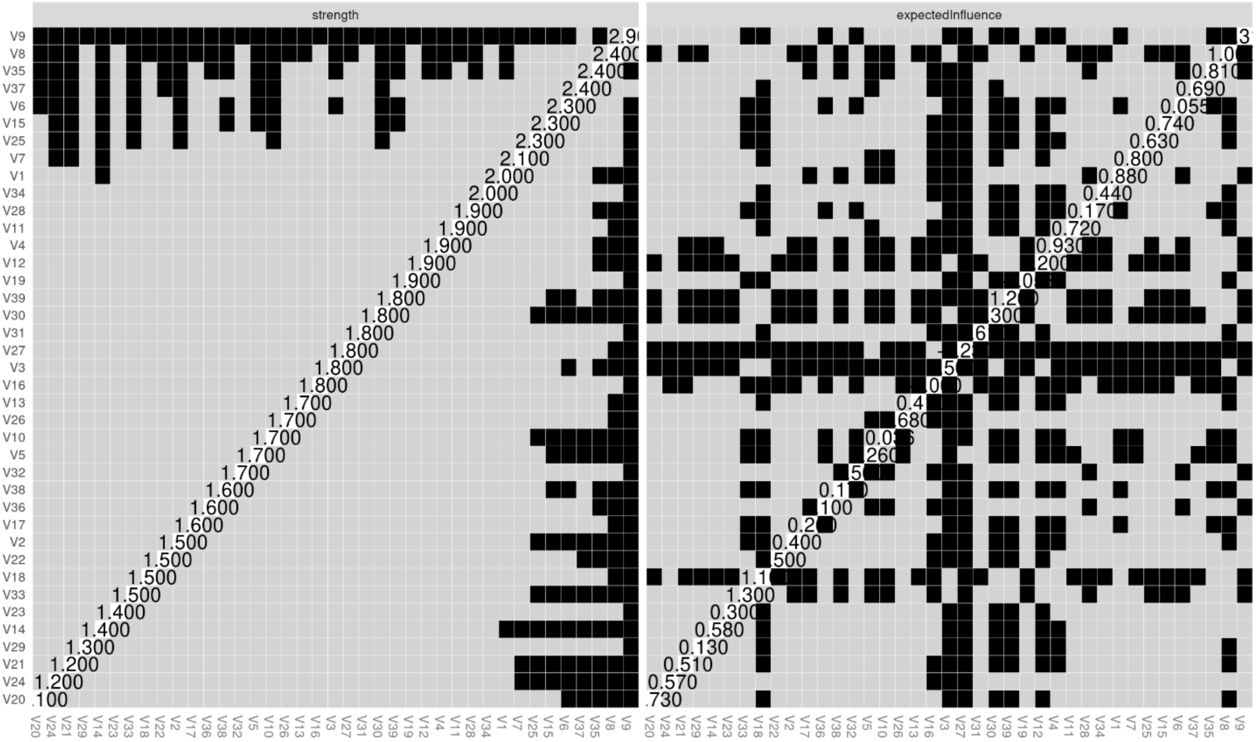

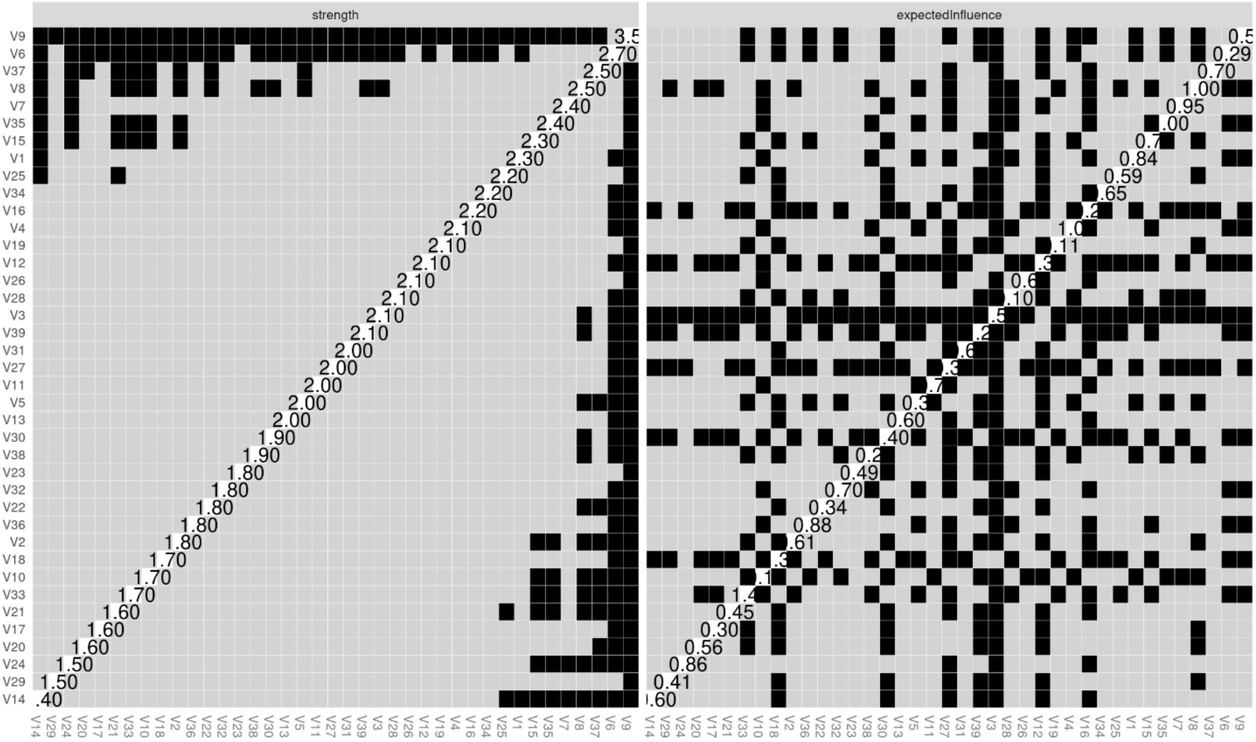

Figure S24 Differences in edges of averaged network A and B in DZ men.

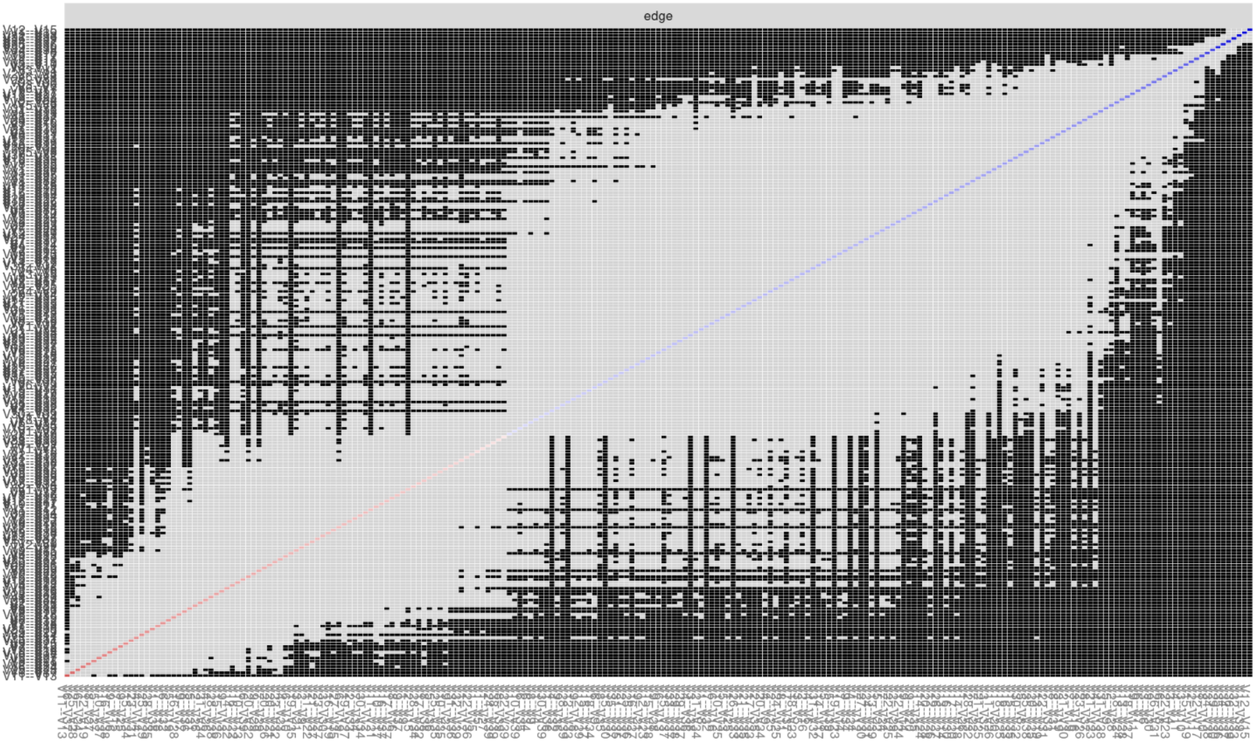

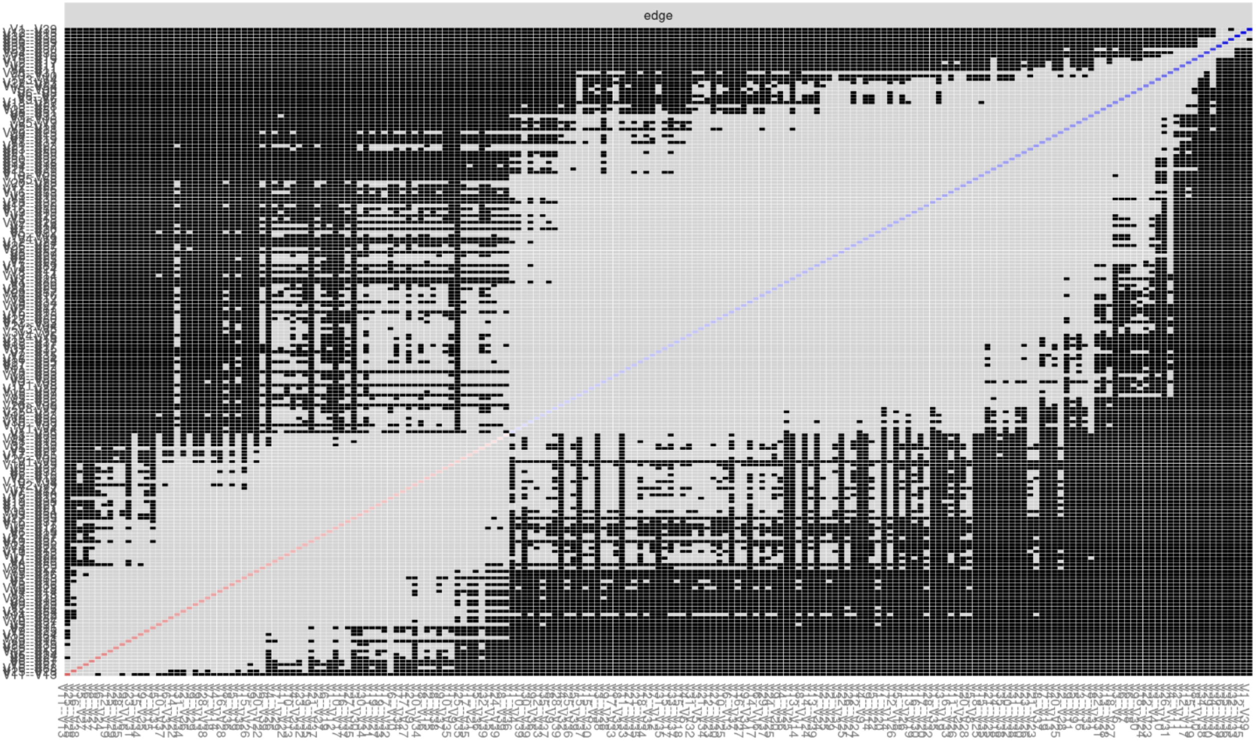

5. Comparison details

Table S2 Comparison of global strength and maximum difference of 1000 reassignments of co-twins within each pair of zygosity-gender dataset.

Gender	Zygosity	Mean value of S[a]	Sd value of S[a]	P-value of S[a]	Mean value of M[b]	Sd value of M[b]	P-value of M[b]
women	MZ	0.4967323	0.2858170	0.9510490	0.5548462	0.3016900	0.9470529
men	MZ	0.5114832	0.2861124	0.9629630	0.5192033	0.3007225	0.9460317
women	DZ	0.5012567	0.2874116	0.9460539	0.5517962	0.2815691	0.9720280
men	DZ	0.5227562	0.2902725	0.9550450	0.5030569	0.2943555	0.9400599

Note. [a] S means global strength. [b] M means maximum difference.

Table S3 Correlation stability coefficient of averaged network A and B in each zygosity-gender pair.

Gender	Zygosity	id	CS[a](Strength)	CS[a](ExpectedInfluence)
women	MZ	1	0.2832244	0.3616558
		2	0.2832244	0.3616558
men	MZ	1	0.2054795	0.3616438
		2	0.2054795	0.3616438
women	DZ	1	0.2049911	0.4385027
		2	0.2834225	0.5169340
men	DZ	1	0.2057026	0.2057026
		2	0.4378819	0.3604888

Note. [a] CS means coefficient of correlation stability.

Network analysis results of comparing MZ and DZ in each gender

1. Results of women

Figure S25 Networks of MZ and DZ in women.

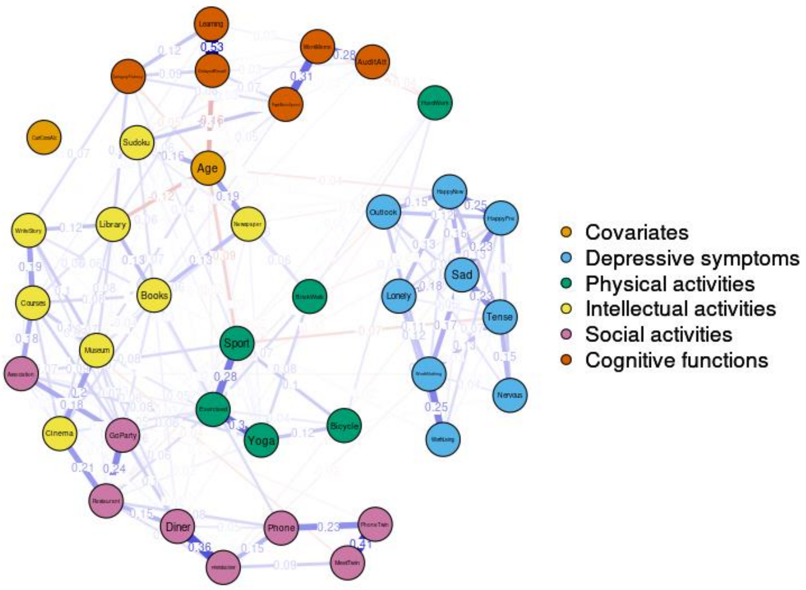

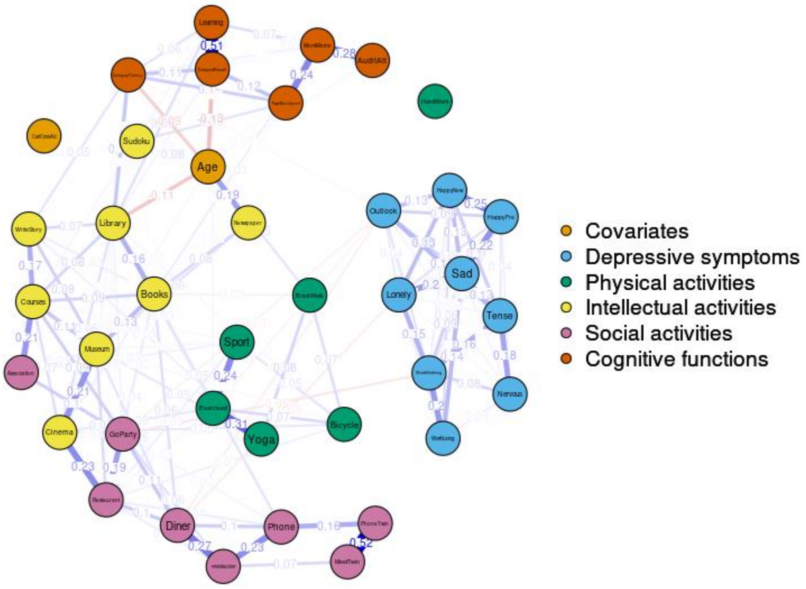

Figure S26 Centrality (Strength and EI) comparisons of MZ and DZ in women.

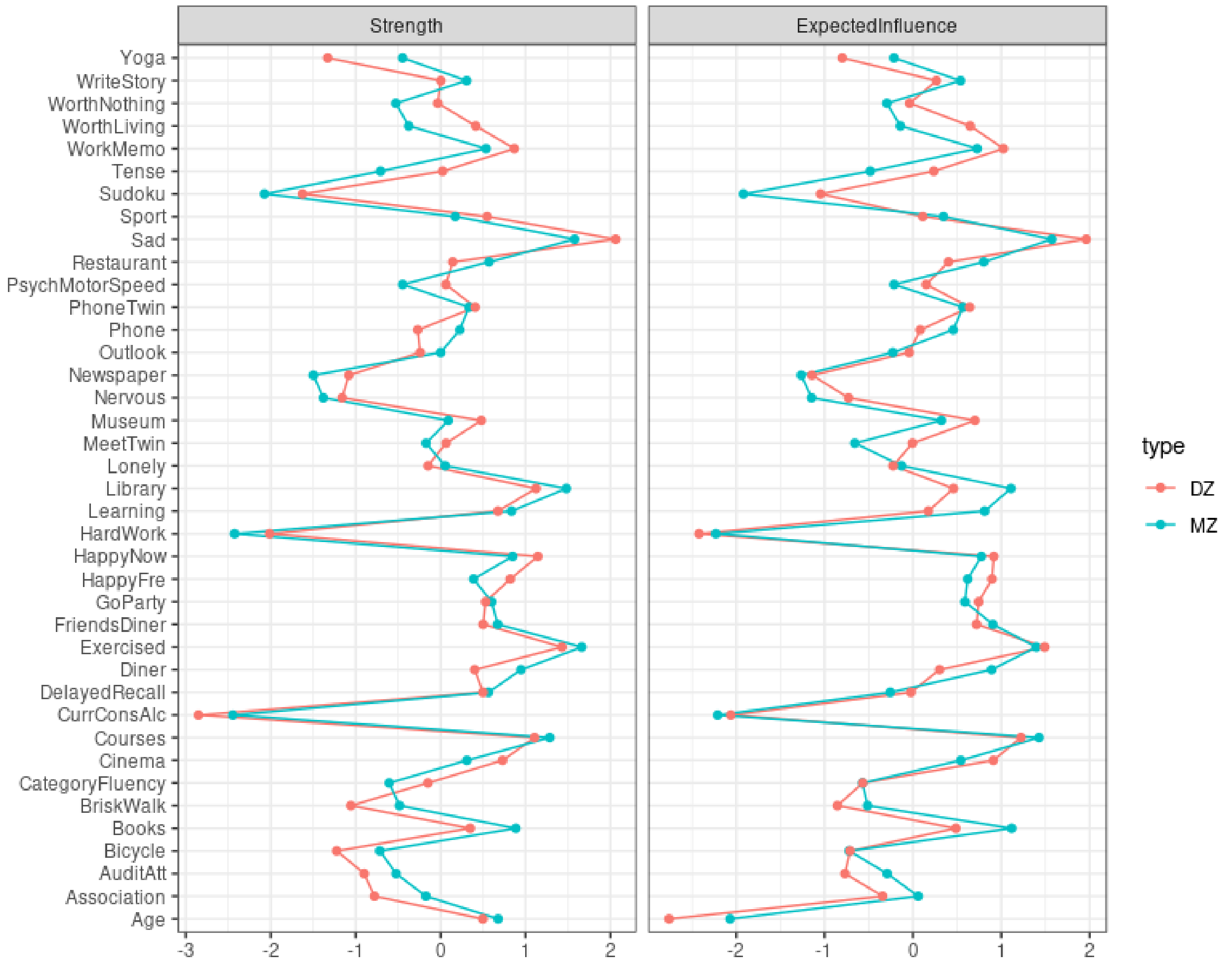

Figure S27 Network comparison in global strength and network structure of MZ and DZ in women.

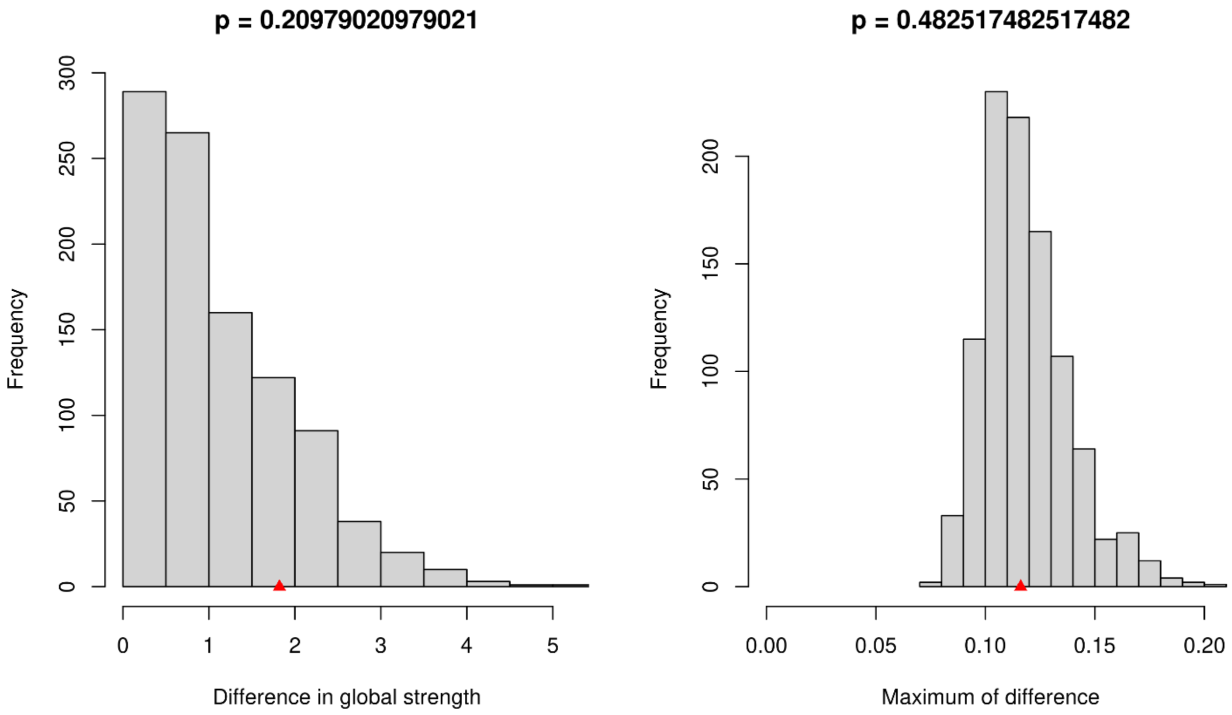

Figure S28 Stability of centrality indices in women. The top represents MZ twins and the bottom represents DZ.

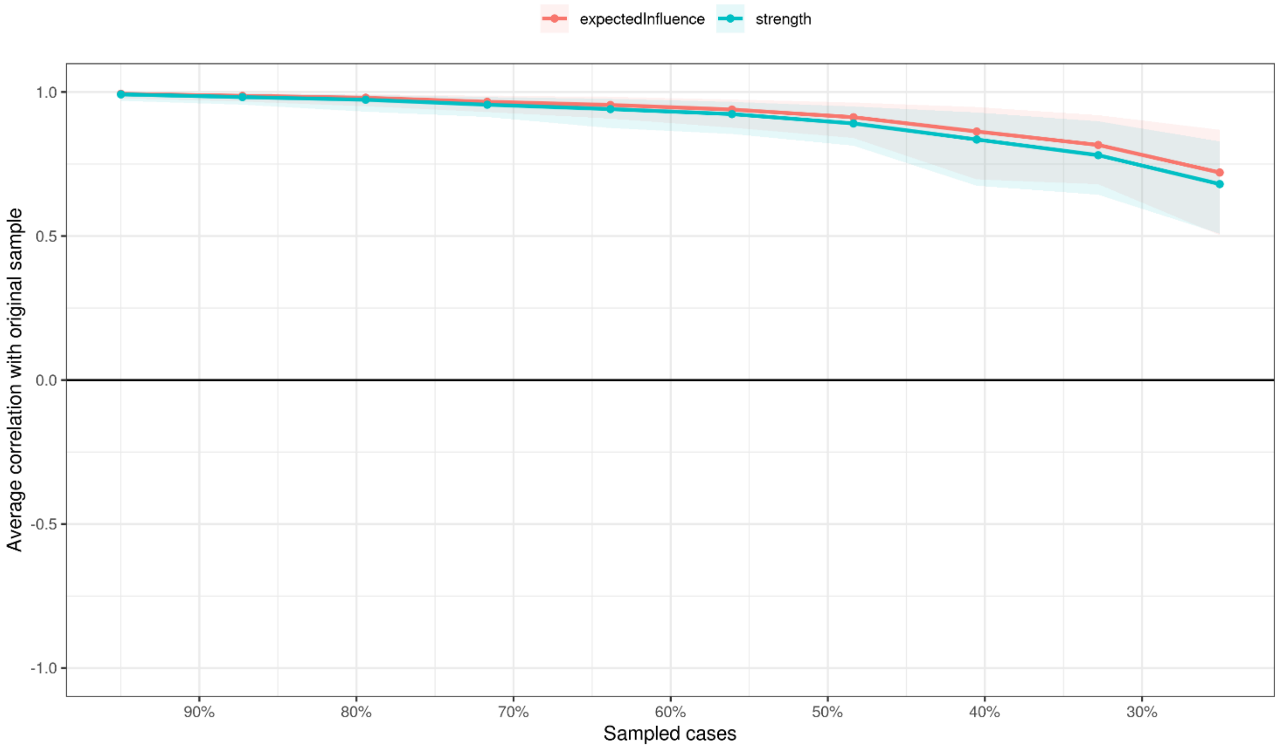

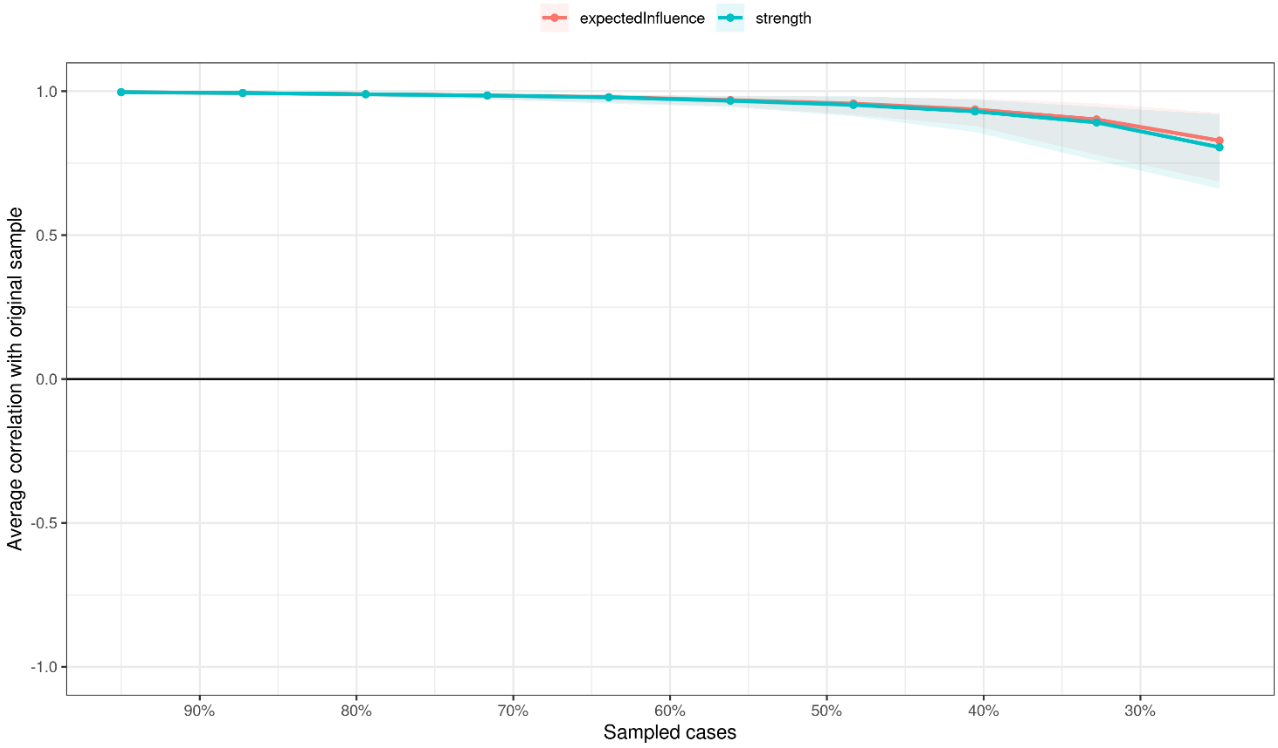

Figure S29 Stability of edges in women. The top represents MZ twins and the bottom represents DZ.

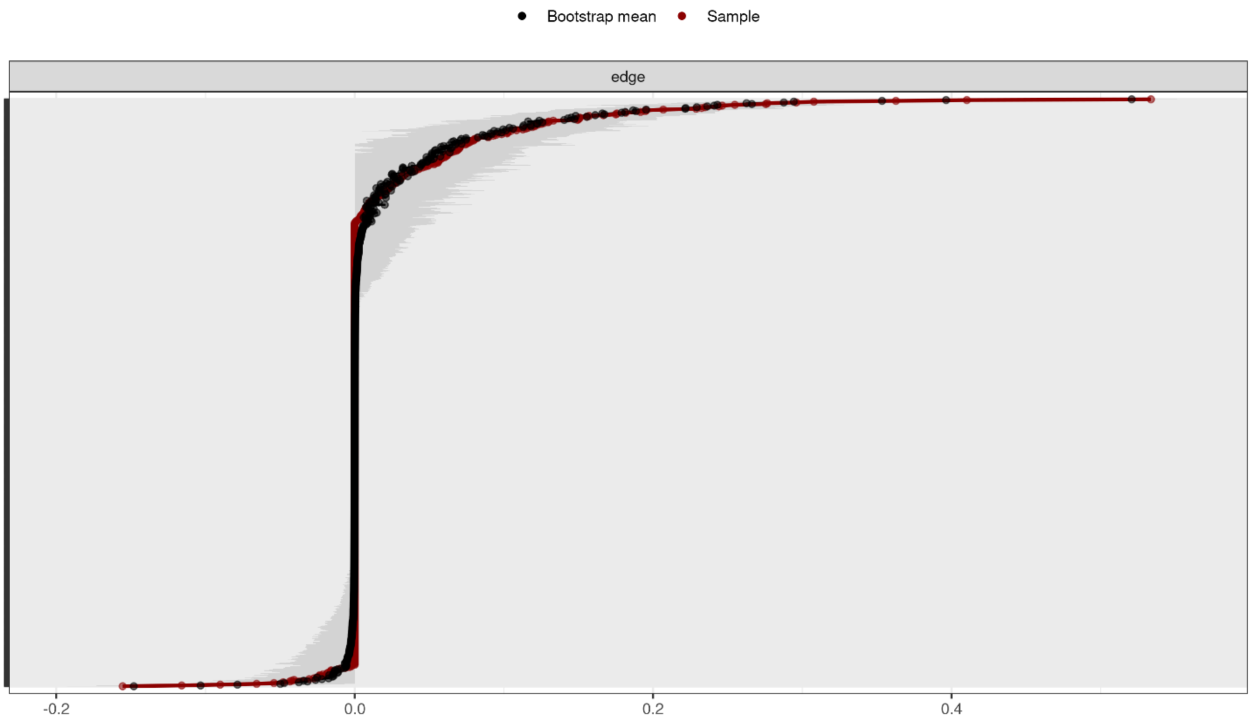

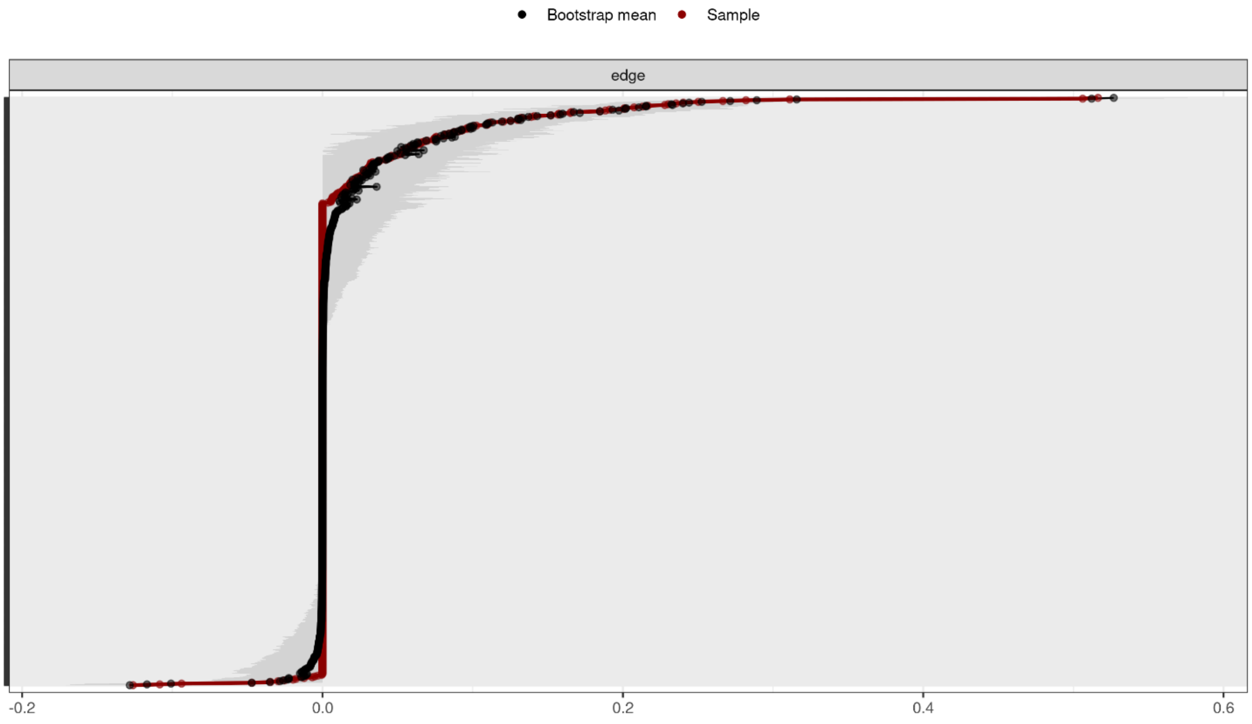

Figure S30 Differences in centrality of nodes in women. The top two represent MZ twins and the bottom represent DZ.

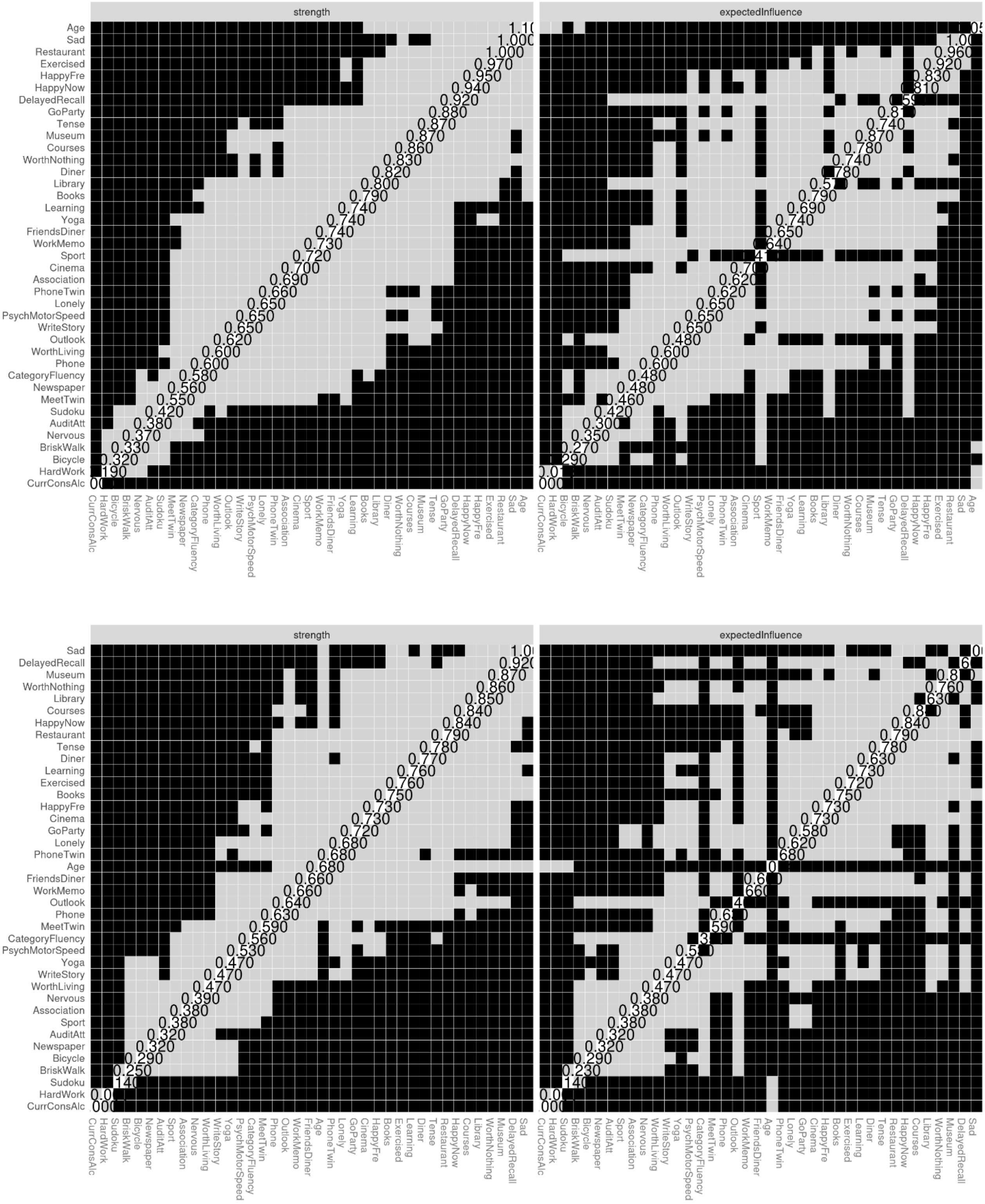

Figure S31 Differences in edges of women. The top represents MZ twins and the bottom represents DZ.

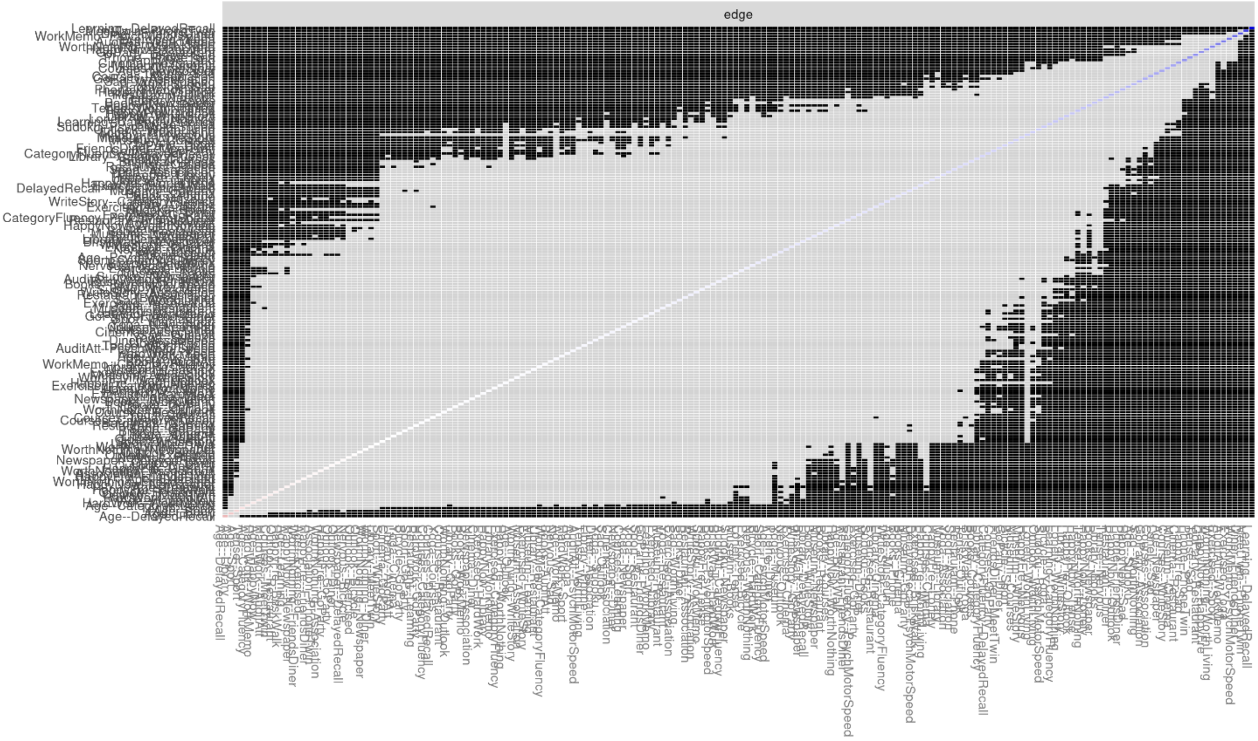

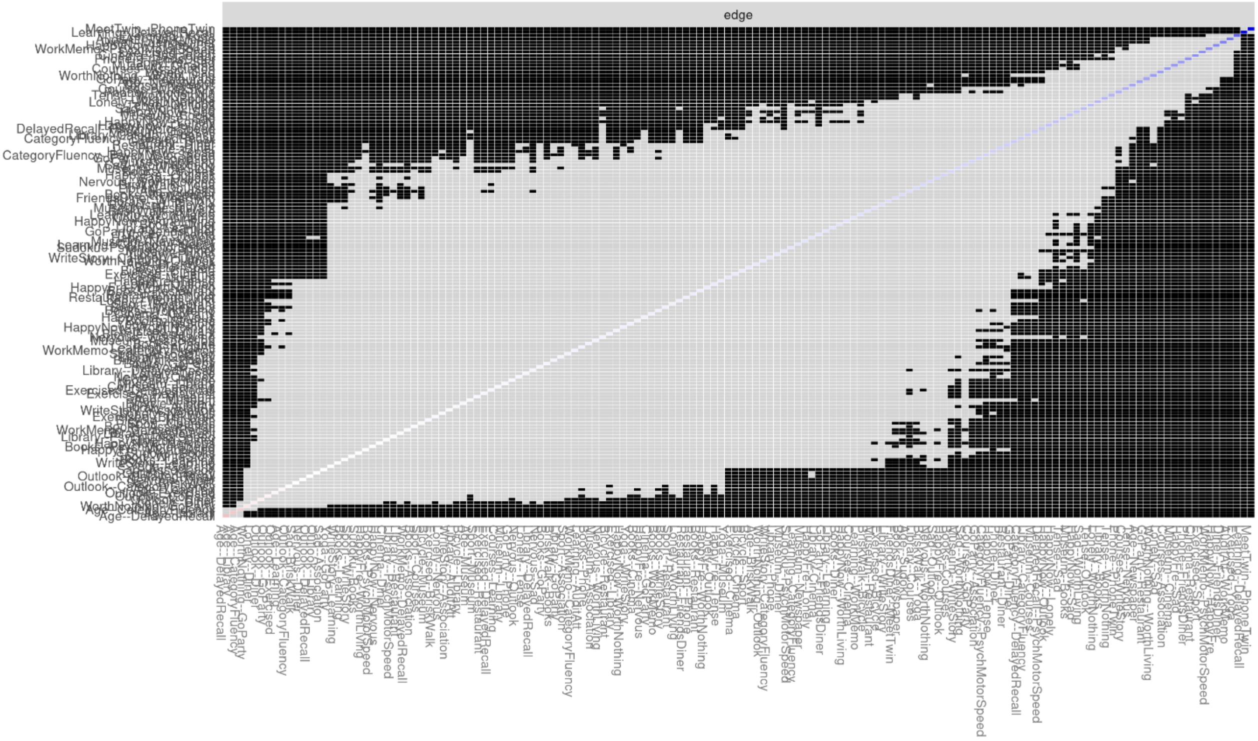

2. Results of men

Figure S32 Networks of MZ and DZ in men.

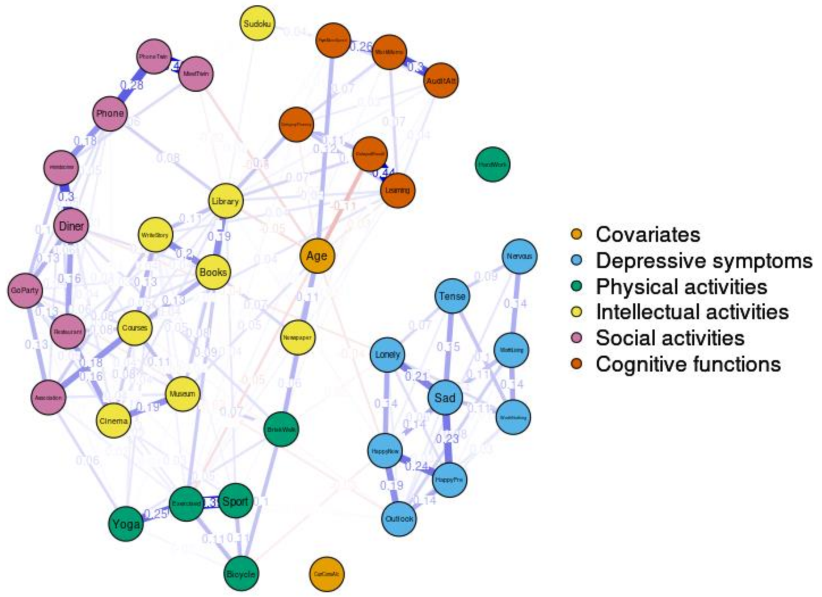

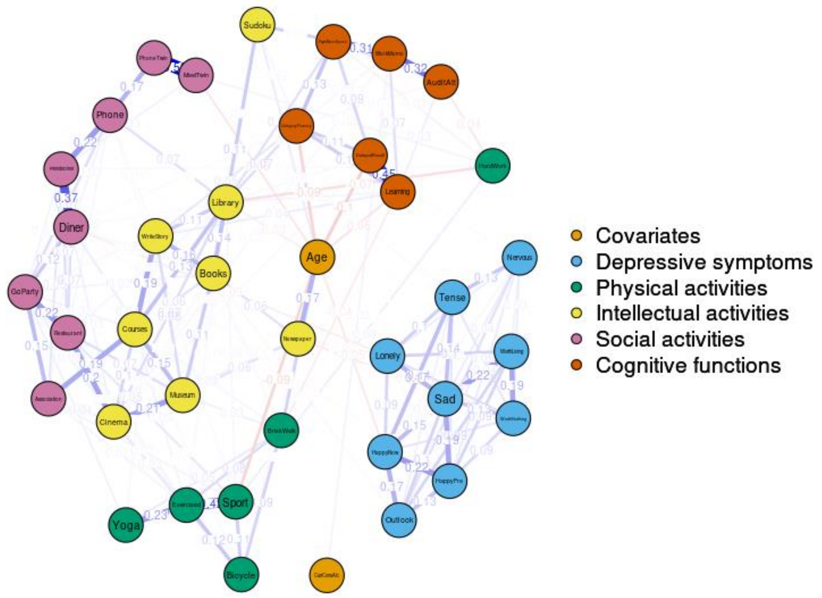

Figure S33 Centrality (Strength and EI) comparisons of MZ and DZ in men.

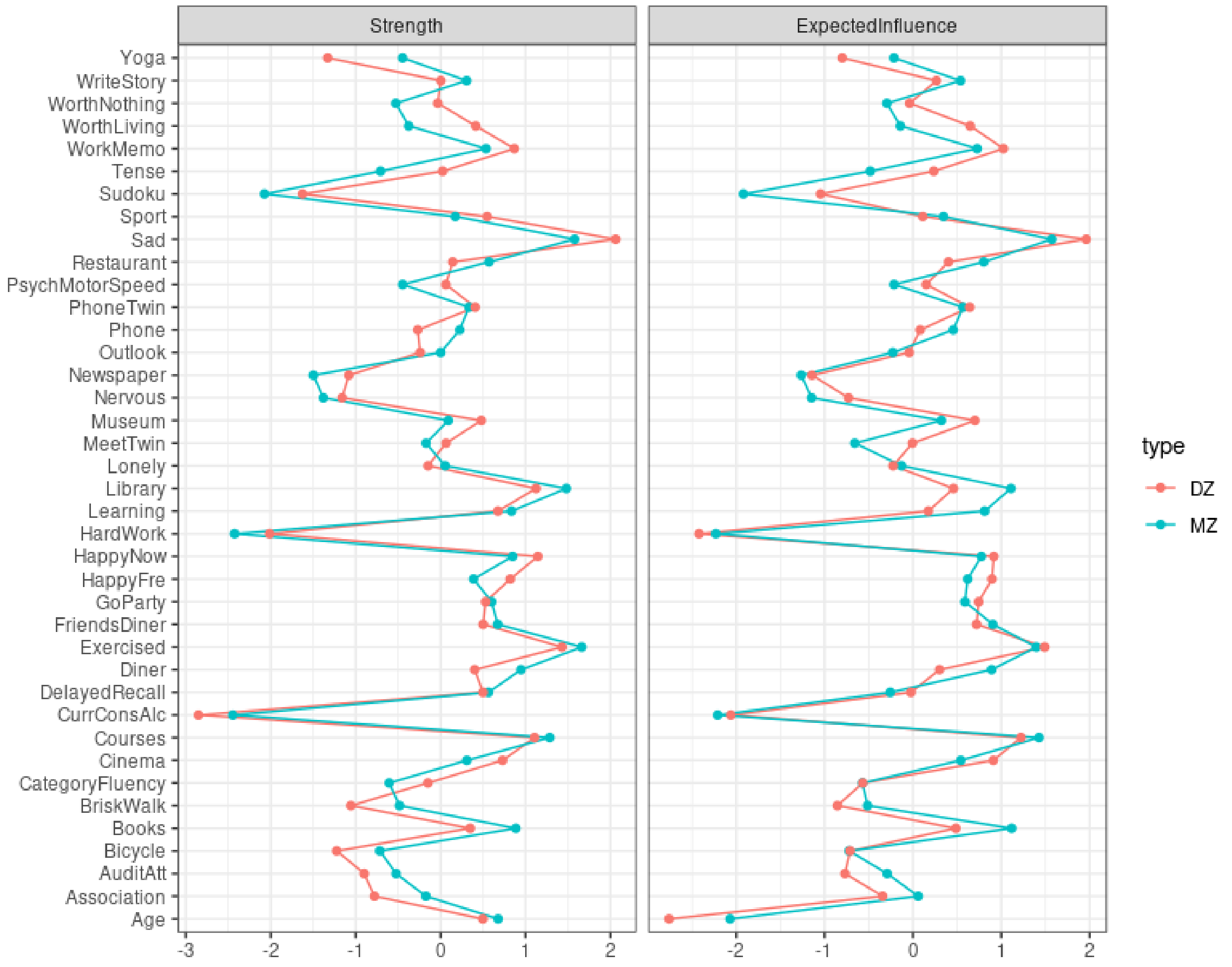

Figure S34 Network comparison in global strength and network structure of MZ and DZ in men.

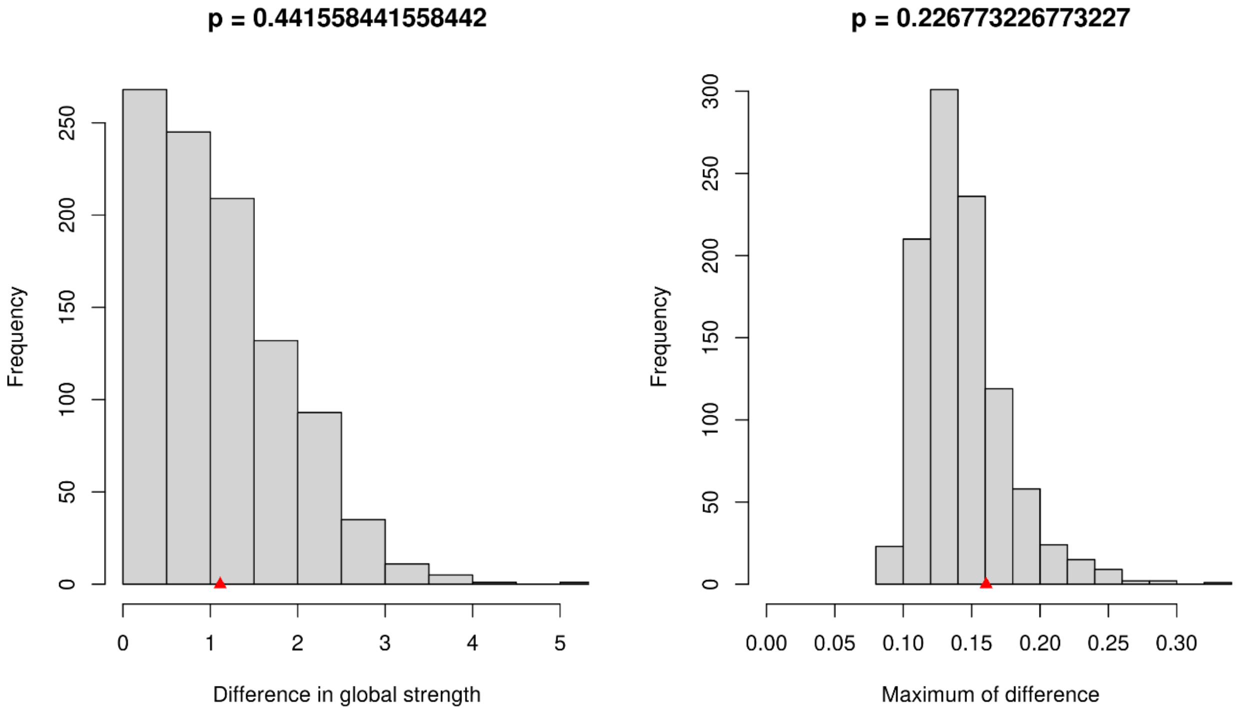

Figure S35 Stability of centrality indices in men. The top represents MZ twins and the bottom represents DZ

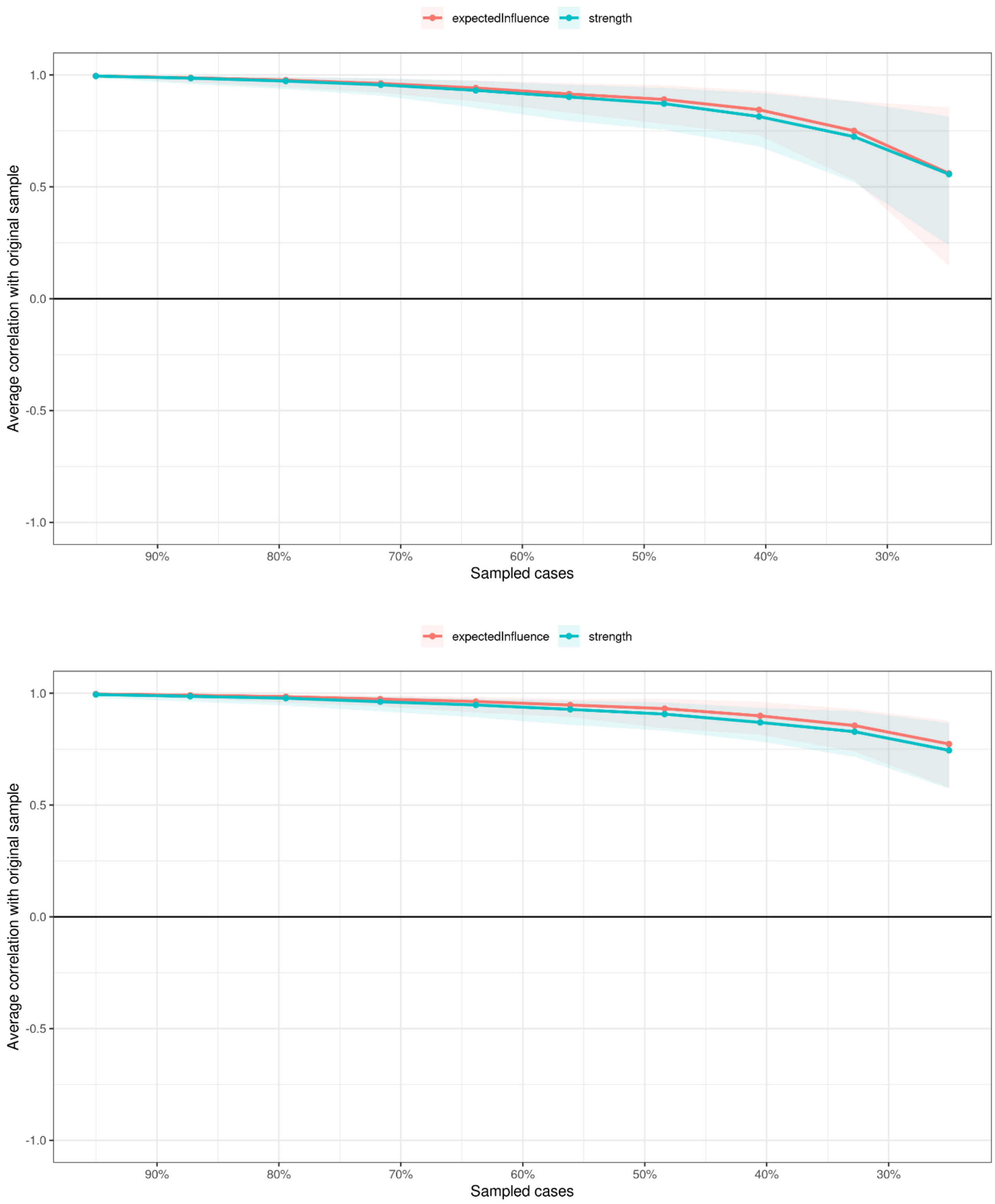

Figure S36 Stability of edges in men. The top represents MZ twins and the bottom represents DZ.

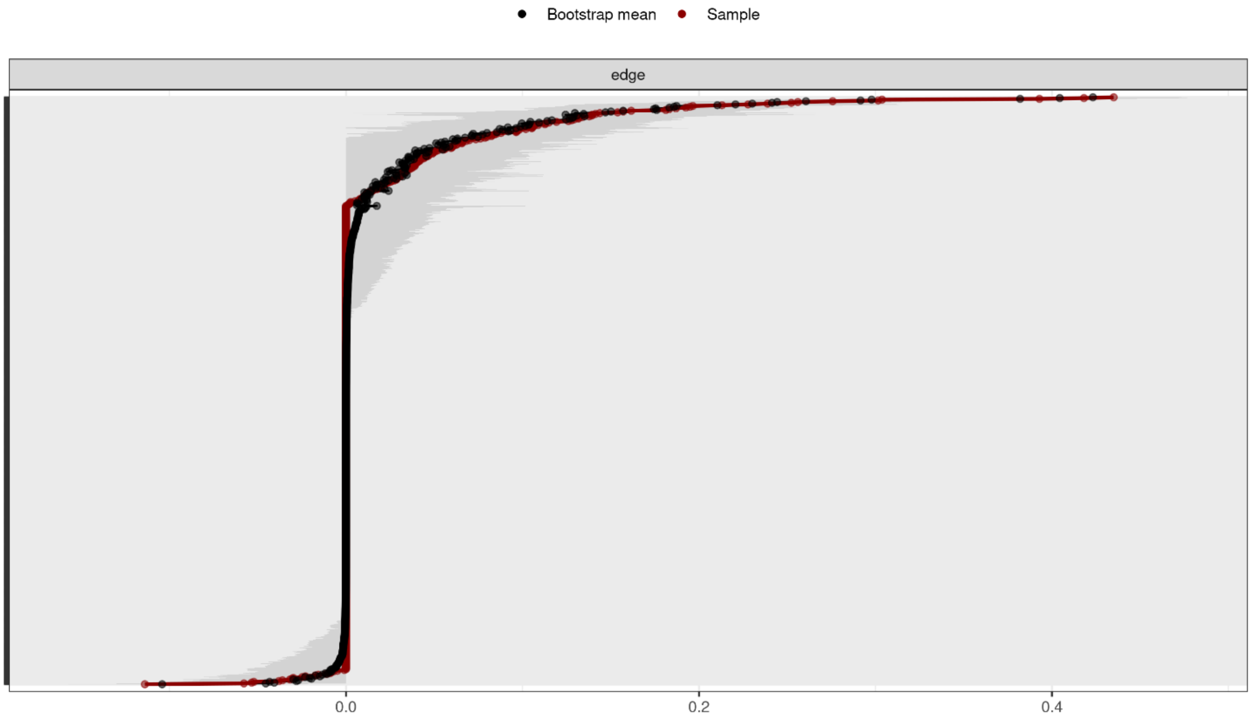

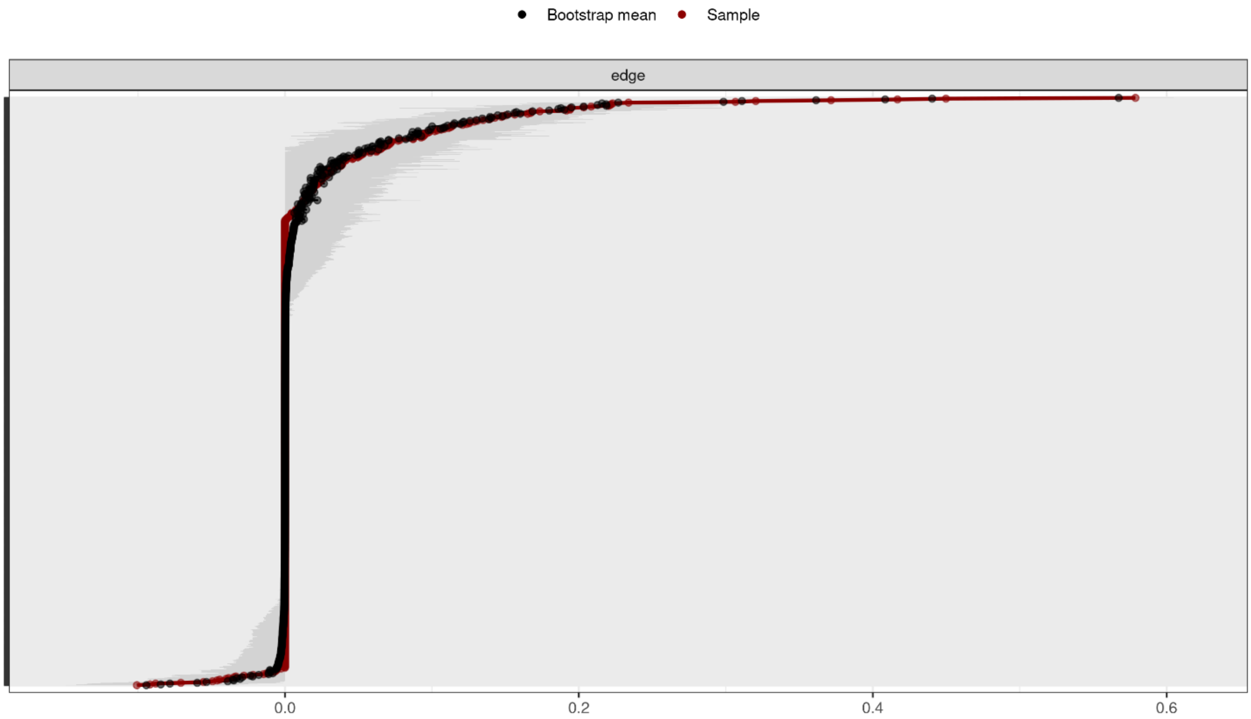

Figure S37 Differences in centrality of nodes in men. The top two represent MZ twins and the bottom represents DZ.

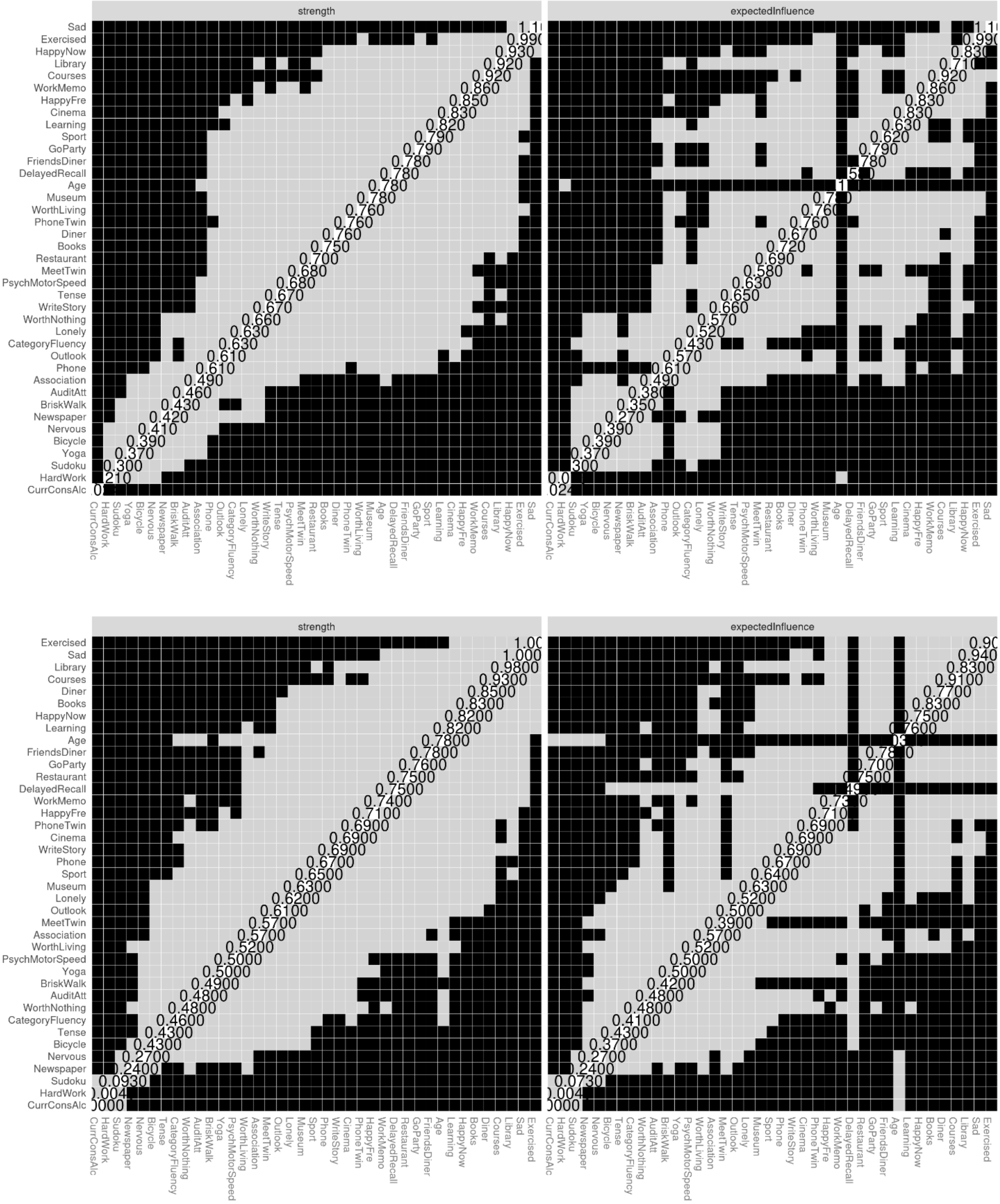

Figure S38 Differences in edges of men. The top represents MZ twins and the bottom represents DZ.

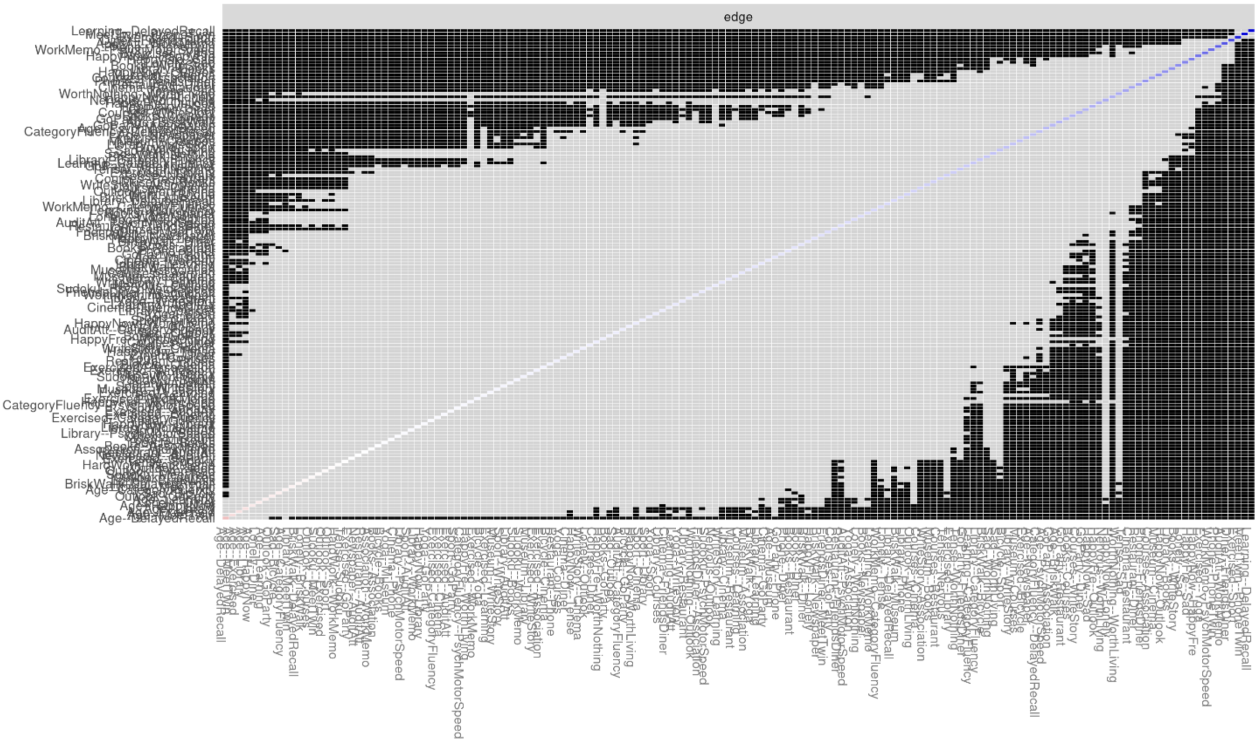

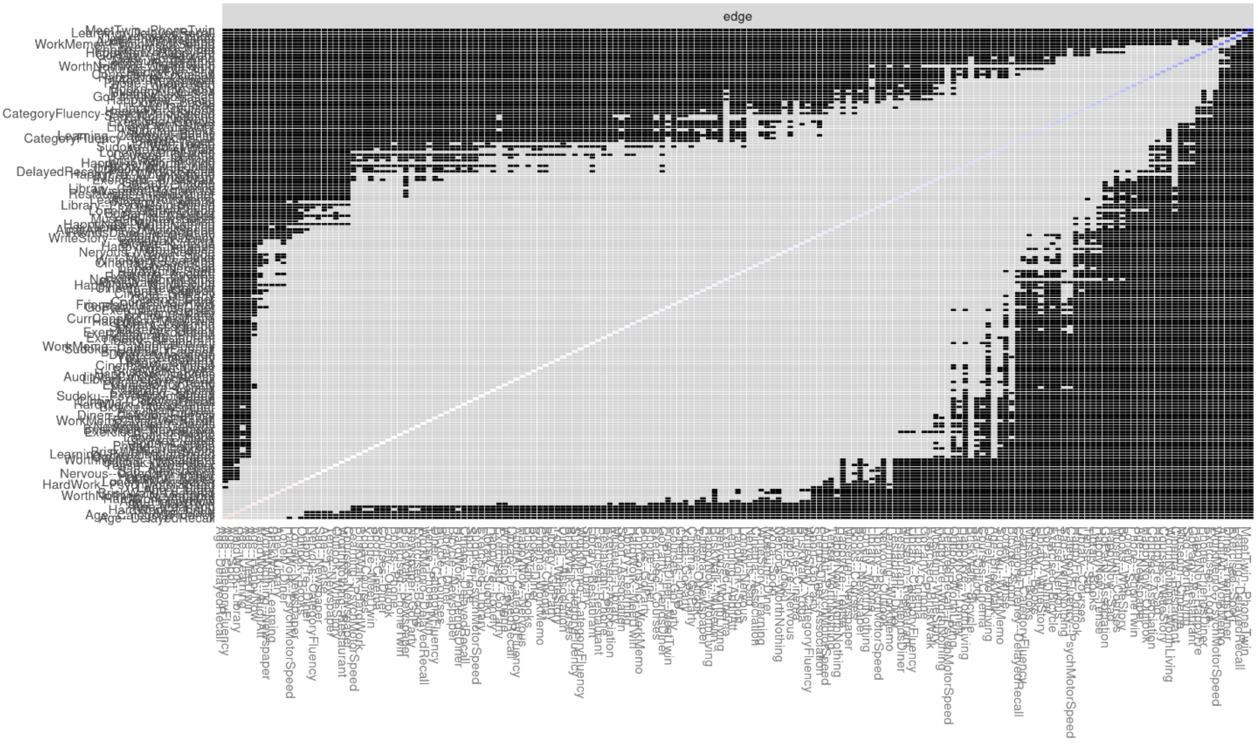

3. Comparison details

Table S4 Comparison of MZ and DZ in each gender

Gender	Zygosity	Difference in S[a]	P-value of S[a]	Difference in M[b]	P-value of M[b]
women	MZDZ	1.82	0.2097902	0.11	0.4825175
men	MZDZ	1.11	0.4415584	0.16	0.2267732

Note. [a] S means global strength. [b] M means maximum difference.

Table S5 Correlation stability coefficient of networks in each gender

Gender	Zygosity	CS[a](Strength)	CS[a](ExpectedInfluence)
women	MZ	0.5947712	0.5947712
women	DZ	0.6720143	0.7504456
men	MZ	0.5164384	0.5945205
men	DZ	0.6720978	0.6720978

Note. [a] CS means coefficient of correlation stability.

Network analysis results across gender

1. Comparison details between gender

Figure S39 Stability of centrality indices. The top represents women and the bottom represents men.

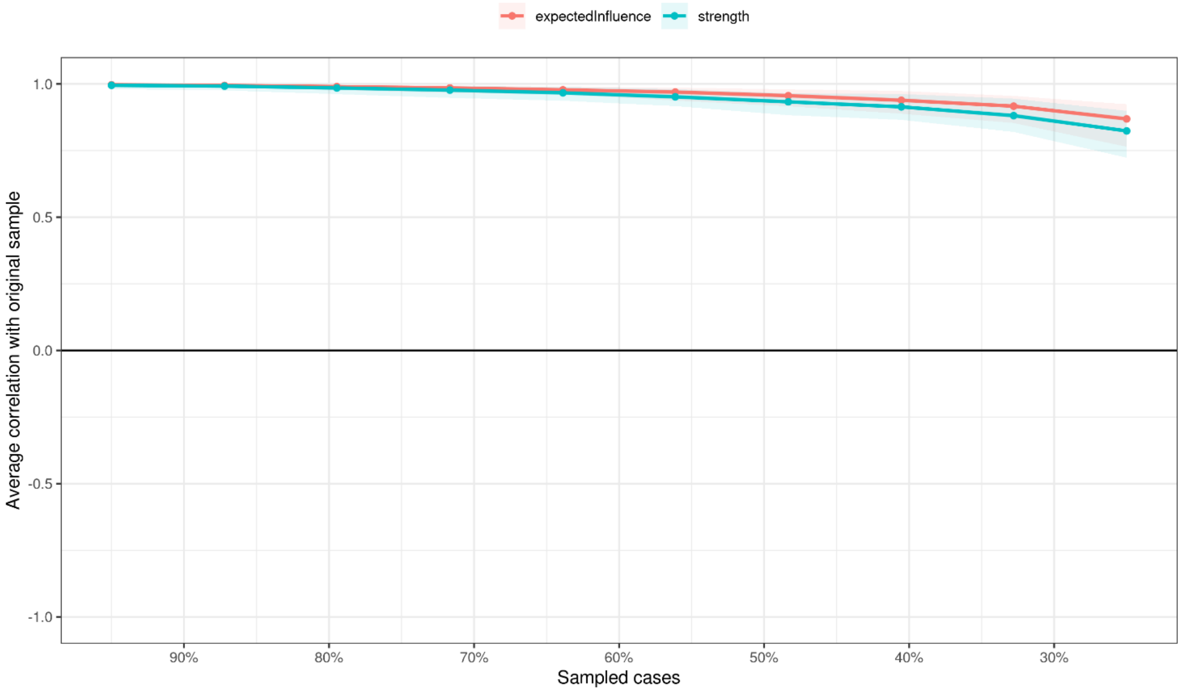

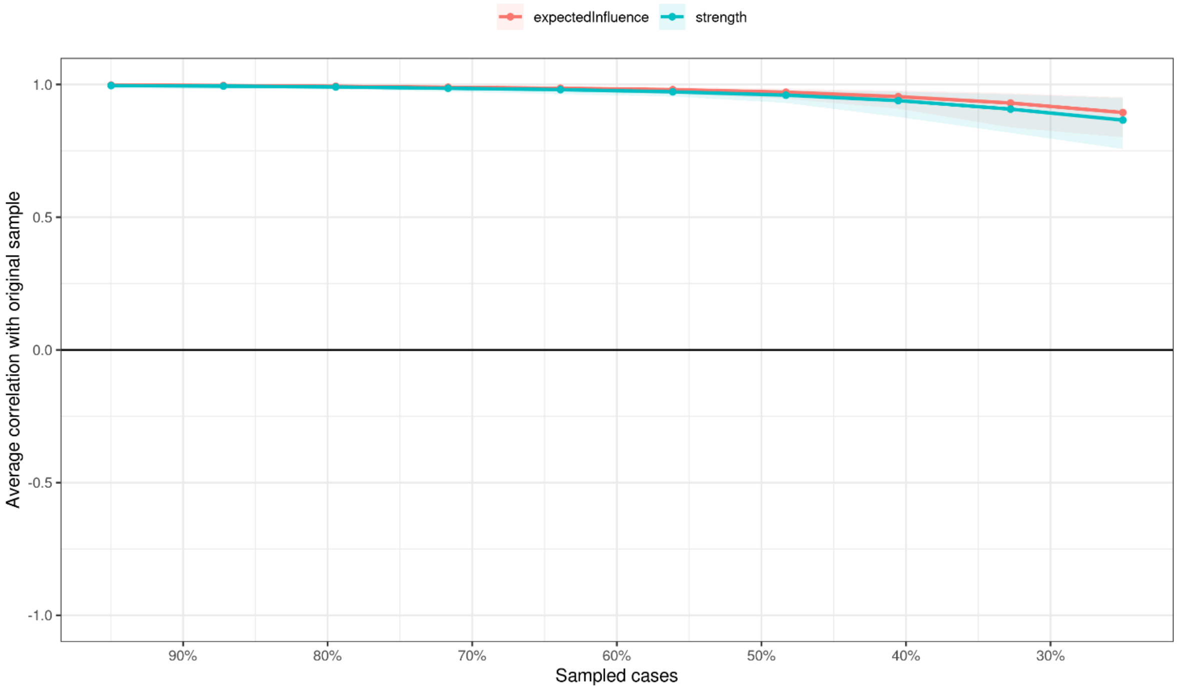

Figure S40 Stability of edges. The top represents women and the bottom represents men.

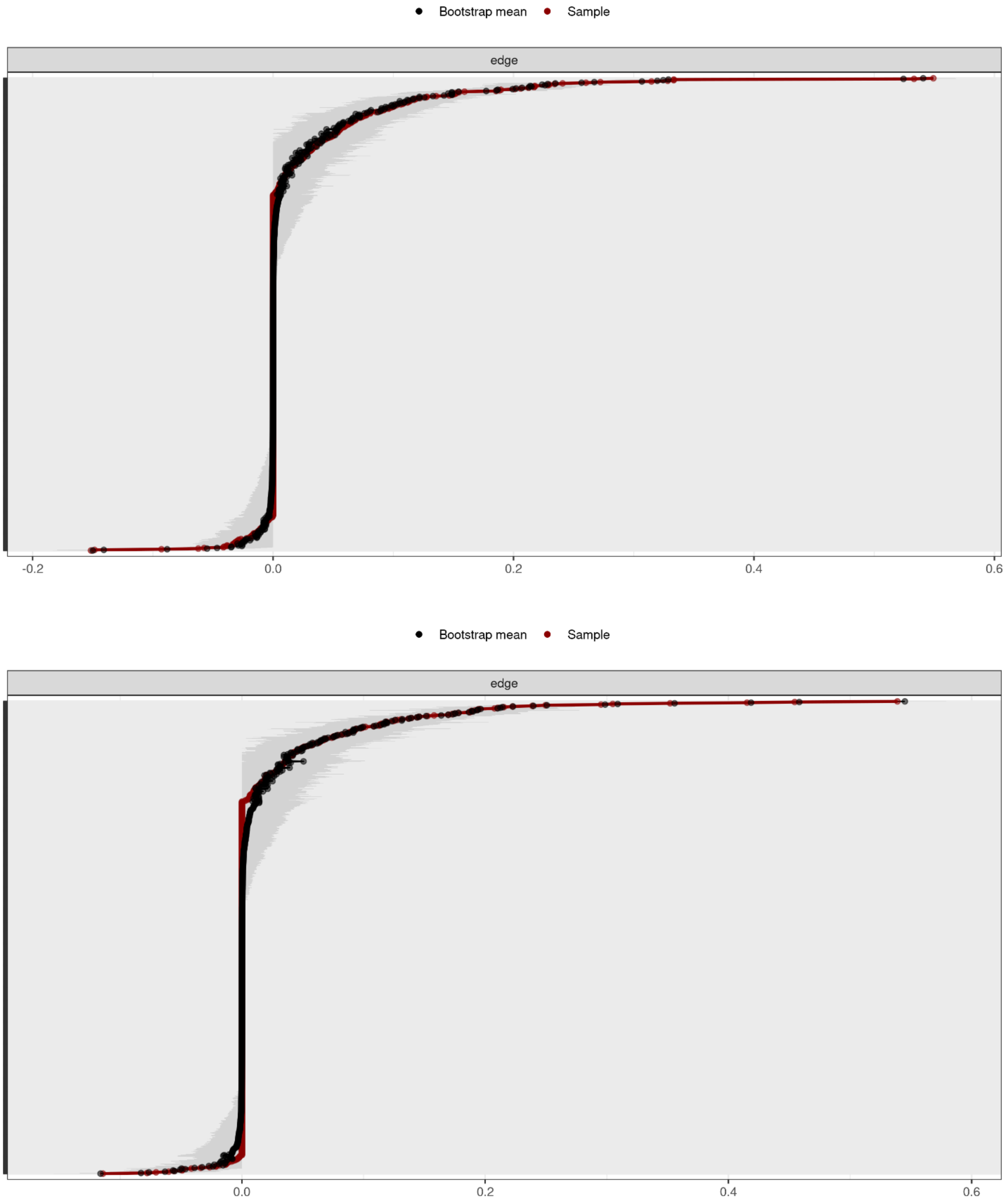

Figure S41 Differences in centrality of nodes. The top two represent women and the bottom represent men.

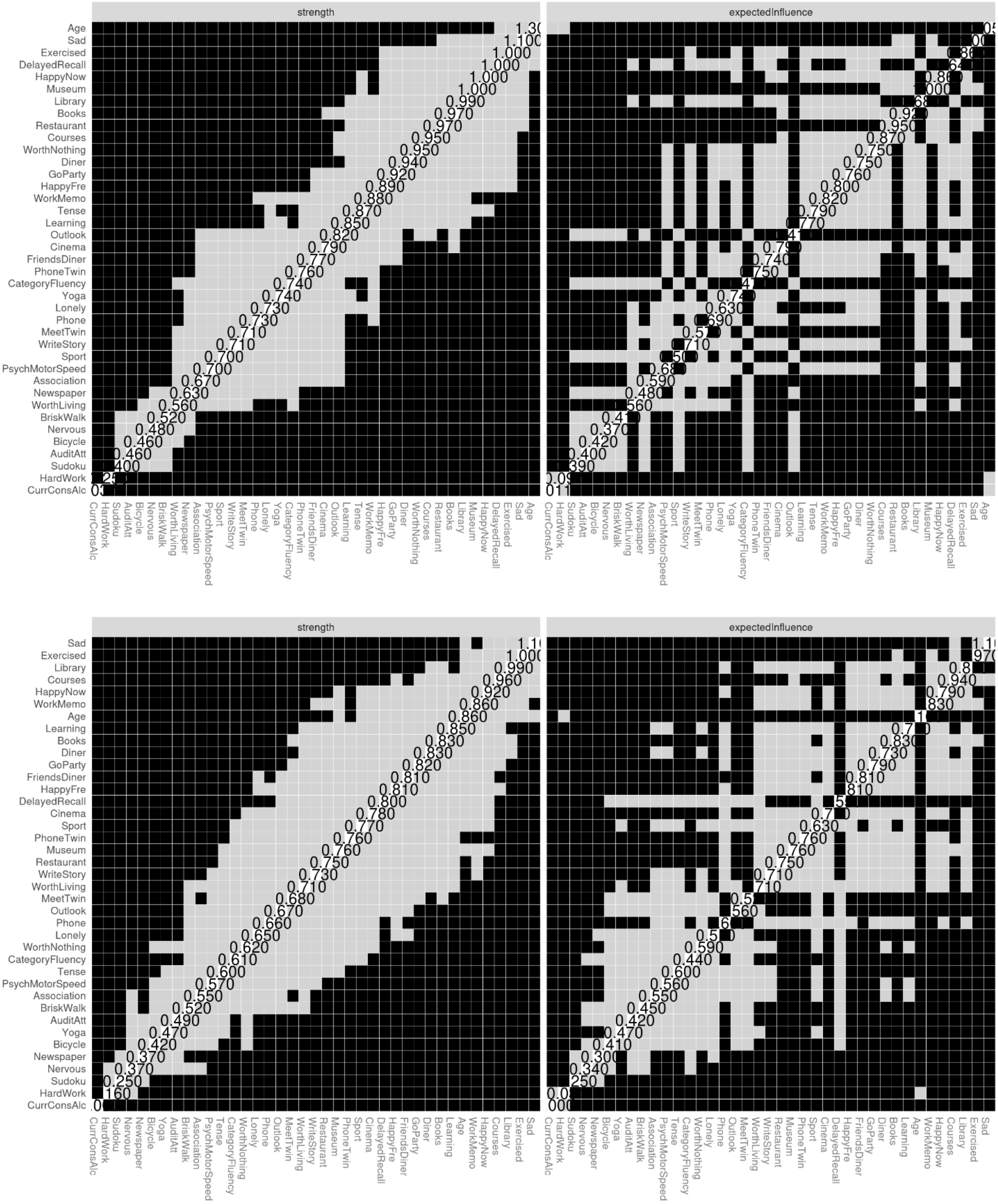

Figure S42 Differences in edges. The top represents women and the bottom represents men.

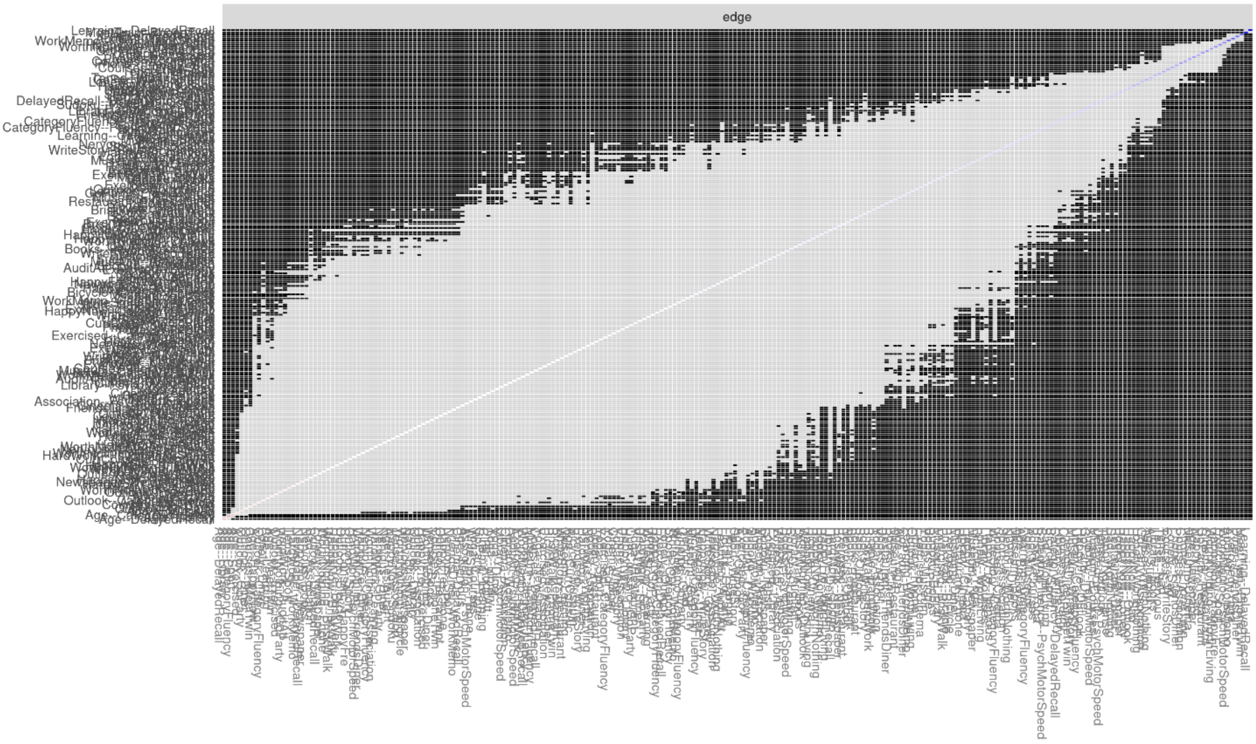

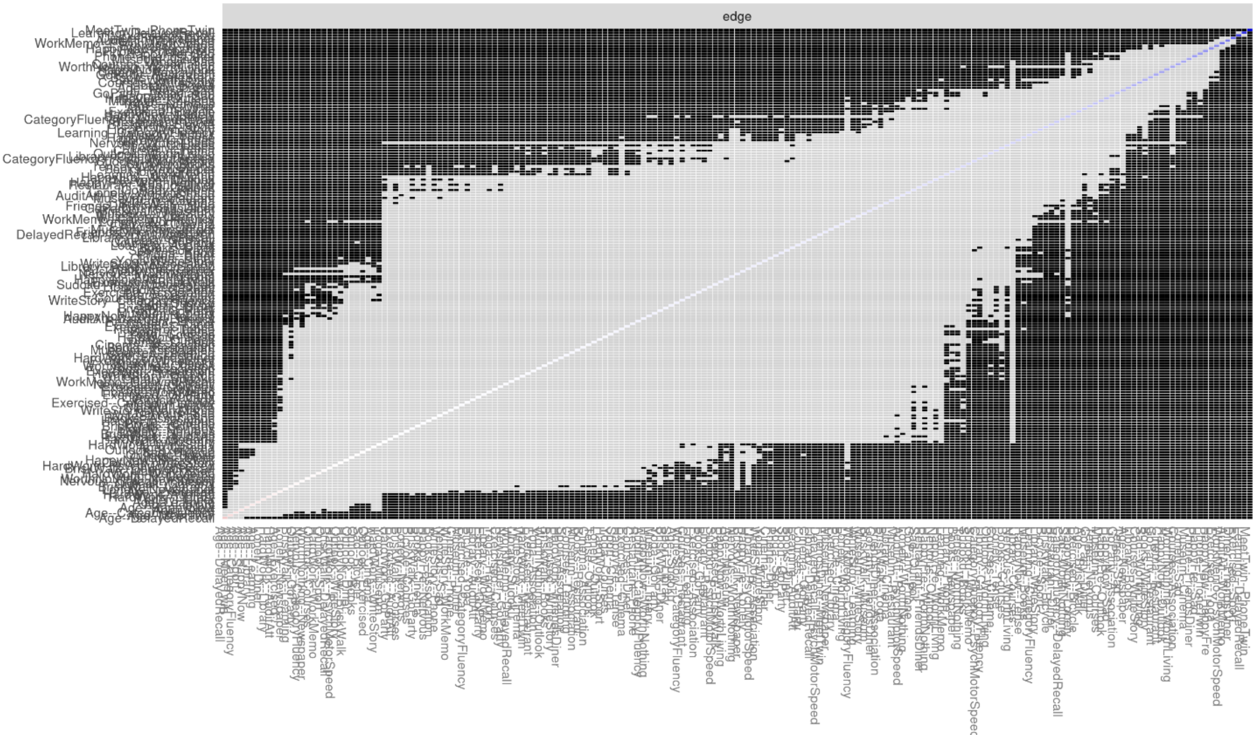

Figure S43 Network comparison in centrality of women and men.

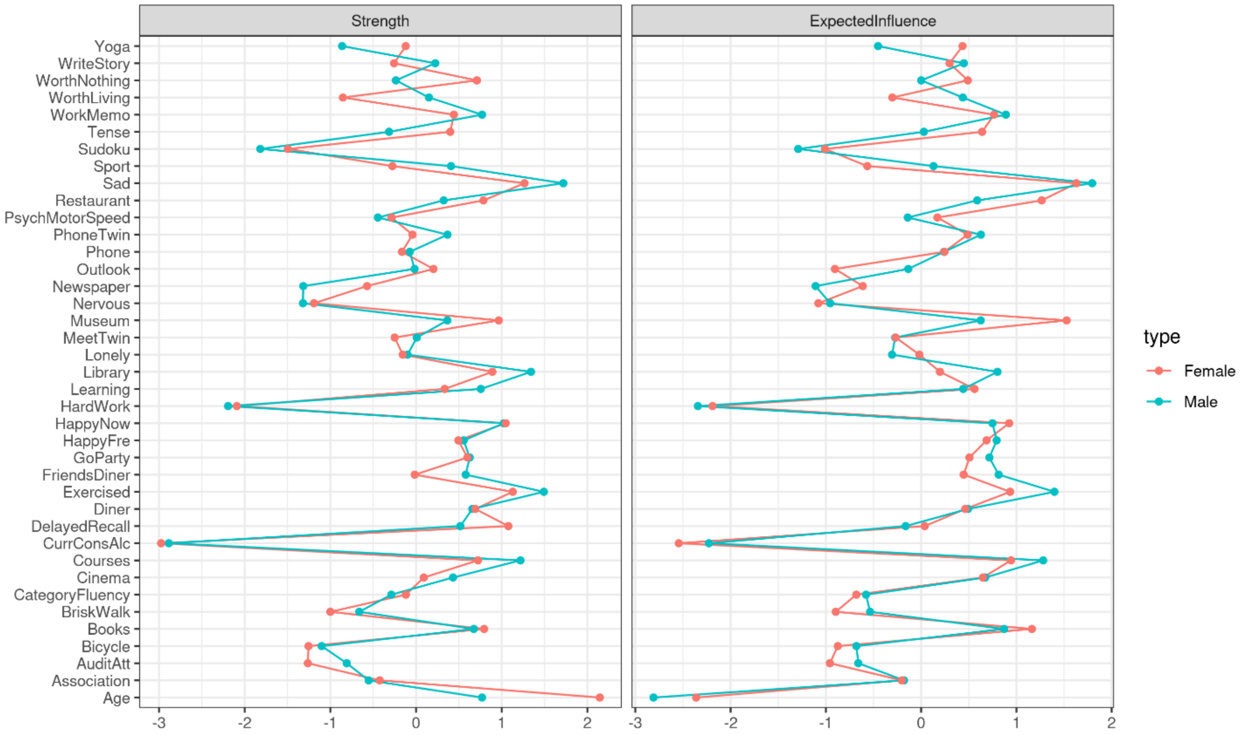

2. Comparison details

Table S6 Correlation stability coefficient in women and men.

Gender	CS[a] (Strength)	CS[a] (ExpectedInfluence)
women	0.75	0.75
men	0.75	0.75

Note. [a] CS means coefficient of correlation stability.

3. Details of centrality and edge across gender

Table S7 Centrality of women network.

Node	Category	Strength	ExpectedInfluence
Age	Covariate	1.3005636	0.0552751
Sad	Depressive symptom	1.0834789	1.0344788
Exercised	Physical activity	1.0498145	0.8638467
DelayedRecall	Cognitive function	1.0370145	0.6439243
HappyNow	Depressive symptom	1.0294125	0.8612641
Museum	Intellectual activity	1.0094376	1.0094376
Library	Intellectual activity	0.9909335	0.6830196
Books	Intellectual activity	0.9672439	0.9198262
Restaurant	Social activity	0.9653388	0.9451544
Courses	Intellectual activity	0.9495987	0.8661140
WorthNothing	Depressive symptom	0.9465153	0.7548240
Diner	Social activity	0.9417497	0.7483498
GoParty	Social activity	0.9191440	0.7590497
HappyFre	Depressive symptom	0.8929984	0.8030356
WorkMemo	Cognitive function	0.8800644	0.8223651
Tense	Depressive symptom	0.8696782	0.7916703
Learning	Cognitive function	0.8535611	0.7721825
Outlook	Depressive symptom	0.8212918	0.4124927
Cinema	Intellectual activity	0.7935769	0.7935769
FriendsDiner	Social activity	0.7672266	0.7441162
PhoneTwin	Social activity	0.7609440	0.7543587
CategoryFluency	Cognitive function	0.7417552	0.4676910
Yoga	Physical activity	0.7413204	0.7413204
Lonely	Depressive symptom	0.7328374	0.6300310
Phone	Social activity	0.7305804	0.6945942
MeetTwin	Social activity	0.7095616	0.5691656
WriteStory	Intellectual activity	0.7078085	0.7078085
Sport	Physical activity	0.7029758	0.4959566
PsychMotorSpeed	Cognitive function	0.7006073	0.6764564
Association	Social activity	0.6663514	0.5850350
Newspaper	Intellectual activity	0.6299685	0.4843314
WorthLiving	Depressive symptom	0.5604678	0.5604678
BriskWalk	Physical activity	0.5242985	0.4148122
Nervous	Depressive symptom	0.4768859	0.3699245
Bicycle	Physical activity	0.4613552	0.4200398
AuditAtt	Cognitive function	0.4593692	0.3997256
Sudoku	Intellectual activity	0.4018404	0.3870376
HardWork	Physical activity	0.2549804	0.0974739
CurrConsAlc	Covariate	0.0366767	0.0105082

Table S8 Centrality of men network.

Node	Category	Strength	ExpectedInfluence
Sad	Depressive symptom	1.0778336	1.0730118
Exercised	Physical activity	1.0245786	0.9672215
Library	Intellectual activity	0.9893013	0.8077941
Courses	Intellectual activity	0.9606708	0.9356842
HappyNow	Depressive symptom	0.9152982	0.7938064
WorkMemo	Cognitive function	0.8557812	0.8313095
Age	Covariate	0.8557607	-0.1550784
Learning	Cognitive function	0.8522741	0.7118176
Books	Intellectual activity	0.8332585	0.8264797
Diner	Social activity	0.8292540	0.7251608
GoParty	Social activity	0.8219355	0.7851315
FriendsDiner	Social activity	0.8111174	0.8111174
HappyFre	Depressive symptom	0.8056215	0.8056215
DelayedRecall	Cognitive function	0.7964581	0.5506544
Cinema	Intellectual activity	0.7764979	0.7733454
Sport	Physical activity	0.7709908	0.6288628
PhoneTwin	Social activity	0.7614246	0.7614246
Museum	Intellectual activity	0.7608950	0.7608950
Restaurant	Social activity	0.7509232	0.7509232
WriteStory	Intellectual activity	0.7278846	0.7136331
WorthLiving	Depressive symptom	0.7107515	0.7107515
MeetTwin	Social activity	0.6775293	0.5215079
Outlook	Depressive symptom	0.6713895	0.5580998
Phone	Social activity	0.6583896	0.6583896
Lonely	Depressive symptom	0.6521841	0.5126845
WorthNothing	Depressive symptom	0.6203046	0.5943315
CategoryFluency	Cognitive function	0.6079616	0.4397796
Tense	Depressive symptom	0.6016601	0.6016601
PsychMotorSpeed	Cognitive function	0.5712120	0.5565517
Association	Social activity	0.5460091	0.5460091
BriskWalk	Physical activity	0.5203289	0.4511222
AuditAtt	Cognitive function	0.4860942	0.4182080
Yoga	Physical activity	0.4734702	0.4734702
Bicycle	Physical activity	0.4177034	0.4128816
Newspaper	Intellectual activity	0.3673111	0.2986795
Nervous	Depressive symptom	0.3664718	0.3394518
Sudoku	Intellectual activity	0.2500291	0.2500291
HardWork	Physical activity	0.1620496	-0.0307864
CurrConsAlc	Covariate	0.0000000	0.0000000

Table S9 Edges of women network.

Var1	Category	Var2	Category	Partial correlation	Absolute value
DelayedRecall	Cognitive function	Learning	Cognitive function	0.5492005	0.5492005
MeetTwin	Social activity	PhoneTwin	Social activity	0.5331261	0.5331261
Diner	Social activity	FriendsDiner	Social activity	0.3333594	0.3333594
Exercised	Physical activity	Yoga	Physical activity	0.3332167	0.3332167
AuditAtt	Cognitive function	WorkMemo	Cognitive function	0.3282662	0.3282662
PsychMotorSpeed	Cognitive function	WorkMemo	Cognitive function	0.3147525	0.3147525
Exercised	Physical activity	Sport	Physical activity	0.2722426	0.2722426
HappyFre	Depressive symptom	HappyNow	Depressive symptom	0.2602717	0.2602717
WorthLiving	Depressive symptom	WorthNothing	Depressive symptom	0.2408191	0.2408191
Cinema	Intellectual activity	Restaurant	Social activity	0.2341735	0.2341735
GoParty	Social activity	Restaurant	Social activity	0.2297475	0.2297475
HappyFre	Depressive symptom	Sad	Depressive symptom	0.2285703	0.2285703
Age	Covariate	Newspaper	Intellectual activity	0.2177449	0.2177449
Cinema	Intellectual activity	Museum	Intellectual activity	0.2175749	0.2175749
Phone	Social activity	PhoneTwin	Social activity	0.2170309	0.2170309
Association	Social activity	Courses	Intellectual activity	0.2124907	0.2124907
FriendsDiner	Social activity	Phone	Social activity	0.2059854	0.2059854
Lonely	Depressive symptom	Sad	Depressive symptom	0.2010249	0.2010249
Courses	Intellectual activity	WriteStory	Intellectual activity	0.1894913	0.1894913
Sad	Depressive symptom	Tense	Depressive symptom	0.1882857	0.1882857
Nervous	Depressive symptom	Tense	Depressive symptom	0.1854190	0.1854190
Books	Intellectual activity	Library	Intellectual activity	0.1591765	0.1591765
Age	Covariate	Sudoku	Intellectual activity	0.1545446	0.1545446
Tense	Depressive symptom	WorthNothing	Depressive symptom	0.1521629	0.1521629
Association	Social activity	GoParty	Social activity	0.1519892	0.1519892
Age	Covariate	DelayedRecall	Cognitive function	-0.1515223	0.1515223
Lonely	Depressive symptom	WorthNothing	Depressive symptom	0.1506603	0.1506603
HappyNow	Depressive symptom	Sad	Depressive symptom	0.1489162	0.1489162
Age	Covariate	Library	Intellectual activity	-0.1485235	0.1485235
HappyNow	Depressive symptom	Outlook	Depressive symptom	0.1465437	0.1465437
HappyNow	Depressive symptom	Lonely	Depressive symptom	0.1356467	0.1356467
Books	Intellectual activity	Newspaper	Intellectual activity	0.1279610	0.1279610
Diner	Social activity	Restaurant	Social activity	0.1271181	0.1271181
Sad	Depressive symptom	WorthNothing	Depressive symptom	0.1222306	0.1222306
HappyNow	Depressive symptom	Tense	Depressive symptom	0.1191277	0.1191277
Outlook	Depressive symptom	Tense	Depressive symptom	0.1180386	0.1180386
DelayedRecall	Cognitive function	PsychMotorSpeed	Cognitive function	0.1169037	0.1169037
Courses	Intellectual activity	Museum	Intellectual activity	0.1138506	0.1138506
PsychMotorSpeed	Cognitive function	Sudoku	Intellectual activity	0.1136225	0.1136225
Diner	Social activity	GoParty	Social activity	0.1125531	0.1125531
HappyFre	Depressive symptom	Outlook	Depressive symptom	0.1116239	0.1116239

Var1	Category	Var2	Category	Partial correlation	Absolute value
CategoryFluency	Cognitive function	Library	Intellectual activity	0.1083398	0.1083398
Museum	Intellectual activity	WriteStory	Intellectual activity	0.1080136	0.1080136
FriendsDiner	Social activity	MeetTwin	Social activity	0.1062375	0.1062375
Courses	Intellectual activity	GoParty	Social activity	0.1029675	0.1029675
Library	Intellectual activity	WriteStory	Intellectual activity	0.1008212	0.1008212
CategoryFluency	Cognitive function	DelayedRecall	Cognitive function	0.1007054	0.1007054
Bicycle	Physical activity	Sport	Physical activity	0.0994553	0.0994553
Sad	Depressive symptom	WorthLiving	Depressive symptom	0.0980428	0.0980428
CategoryFluency	Cognitive function	PsychMotorSpeed	Cognitive function	0.0964212	0.0964212
BriskWalk	Physical activity	Yoga	Physical activity	0.0957809	0.0957809
Age	Covariate	CategoryFluency	Cognitive function	-0.0929002	0.0929002
Books	Intellectual activity	Courses	Intellectual activity	0.0925181	0.0925181
Age	Covariate	Museum	Intellectual activity	0.0918008	0.0918008
CategoryFluency	Cognitive function	Learning	Cognitive function	0.0900463	0.0900463
Age	Covariate	BriskWalk	Physical activity	0.0887775	0.0887775
Books	Intellectual activity	Museum	Intellectual activity	0.0857900	0.0857900
Diner	Social activity	Phone	Social activity	0.0808923	0.0808923
Nervous	Depressive symptom	WorthNothing	Depressive symptom	0.0807839	0.0807839
Association	Social activity	Sport	Physical activity	0.0777661	0.0777661
Cinema	Intellectual activity	Courses	Intellectual activity	0.0777496	0.0777496
CategoryFluency	Cognitive function	WriteStory	Intellectual activity	0.0775131	0.0775131
HappyFre	Depressive symptom	Nervous	Depressive symptom	0.0759786	0.0759786
Courses	Intellectual activity	Library	Intellectual activity	0.0744383	0.0744383
Lonely	Depressive symptom	WorthLiving	Depressive symptom	0.0736197	0.0736197
Bicycle	Physical activity	Yoga	Physical activity	0.0722688	0.0722688
Museum	Intellectual activity	Newspaper	Intellectual activity	0.0692128	0.0692128
HappyFre	Depressive symptom	Lonely	Depressive symptom	0.0691291	0.0691291
Cinema	Intellectual activity	Library	Intellectual activity	0.0683141	0.0683141
Phone	Social activity	Restaurant	Social activity	0.0681913	0.0681913
Diner	Social activity	Museum	Intellectual activity	0.0669383	0.0669383
Bicycle	Physical activity	Exercised	Physical activity	0.0660307	0.0660307
Bicycle	Physical activity	BriskWalk	Physical activity	0.0653048	0.0653048
BriskWalk	Physical activity	Exercised	Physical activity	0.0647127	0.0647127
Library	Intellectual activity	Museum	Intellectual activity	0.0631136	0.0631136
Books	Intellectual activity	Sudoku	Intellectual activity	0.0629280	0.0629280
Age	Covariate	Sport	Physical activity	-0.0622591	0.0622591
Books	Intellectual activity	Phone	Social activity	0.0616868	0.0616868
Museum	Intellectual activity	Yoga	Physical activity	0.0604169	0.0604169
Cinema	Intellectual activity	Exercised	Physical activity	0.0596635	0.0596635
Library	Intellectual activity	Phone	Social activity	0.0595123	0.0595123
Outlook	Depressive symptom	WorthLiving	Depressive symptom	0.0581176	0.0581176
Age	Covariate	Exercised	Physical activity	-0.0573941	0.0573941
Learning	Cognitive function	WorkMemo	Cognitive function	0.0565404	0.0565404

Var1	Category	Var2	Category	Partial correlation	Absolute value
FriendsDiner	Social activity	GoParty	Social activity	0.0559024	0.0559024
Age	Covariate	Yoga	Physical activity	0.0557662	0.0557662
Museum	Intellectual activity	Restaurant	Social activity	0.0553987	0.0553987
Books	Intellectual activity	Cinema	Intellectual activity	0.0543113	0.0543113
FriendsDiner	Social activity	Restaurant	Social activity	0.0541867	0.0541867
Books	Intellectual activity	Diner	Social activity	0.0536504	0.0536504
Association	Social activity	Yoga	Physical activity	0.0536495	0.0536495
Bicycle	Physical activity	HardWork	Physical activity	0.0518637	0.0518637
BriskWalk	Physical activity	HardWork	Physical activity	0.0507198	0.0507198
Books	Intellectual activity	WriteStory	Intellectual activity	0.0504792	0.0504792
Outlook	Depressive symptom	Sad	Depressive symptom	0.0503403	0.0503403
Books	Intellectual activity	Restaurant	Social activity	0.0500226	0.0500226
Restaurant	Social activity	Sport	Physical activity	0.0489955	0.0489955
Nervous	Depressive symptom	Outlook	Depressive symptom	0.0480847	0.0480847
Exercised	Physical activity	Restaurant	Social activity	0.0467173	0.0467173
BriskWalk	Physical activity	Newspaper	Intellectual activity	0.0452454	0.0452454
HardWork	Physical activity	Sport	Physical activity	0.0434314	0.0434314
DelayedRecall	Cognitive function	Library	Intellectual activity	0.0433237	0.0433237
HappyFre	Depressive symptom	WorthLiving	Depressive symptom	0.0414719	0.0414719
GoParty	Social activity	Outlook	Depressive symptom	-0.0414034	0.0414034
Diner	Social activity	Outlook	Depressive symptom	-0.0407012	0.0407012
Books	Intellectual activity	WorkMemo	Cognitive function	0.0406517	0.0406517
HappyNow	Depressive symptom	WorthNothing	Depressive symptom	0.0398707	0.0398707
Courses	Intellectual activity	MeetTwin	Social activity	-0.0384497	0.0384497
Bicycle	Physical activity	Cinema	Intellectual activity	0.0383981	0.0383981
HappyNow	Depressive symptom	WorthLiving	Depressive symptom	0.0383249	0.0383249
Age	Covariate	HappyNow	Depressive symptom	-0.0382097	0.0382097
Outlook	Depressive symptom	WorthNothing	Depressive symptom	0.0381926	0.0381926
Books	Intellectual activity	BriskWalk	Physical activity	0.0367426	0.0367426
AuditAtt	Cognitive function	Library	Intellectual activity	0.0363321	0.0363321
Courses	Intellectual activity	Learning	Cognitive function	0.0358895	0.0358895
Books	Intellectual activity	PsychMotorSpeed	Cognitive function	0.0356697	0.0356697
HappyFre	Depressive symptom	Tense	Depressive symptom	0.0350220	0.0350220
Association	Social activity	WriteStory	Intellectual activity	0.0344750	0.0344750
CategoryFluency	Cognitive function	Outlook	Depressive symptom	-0.0336071	0.0336071
Diner	Social activity	Lonely	Depressive symptom	-0.0334351	0.0334351
Exercised	Physical activity	Library	Intellectual activity	0.0331234	0.0331234
Lonely	Depressive symptom	Tense	Depressive symptom	0.0326183	0.0326183
Newspaper	Intellectual activity	Sudoku	Intellectual activity	0.0324675	0.0324675
Age	Covariate	Learning	Cognitive function	-0.0323251	0.0323251
Association	Social activity	Museum	Intellectual activity	0.0321958	0.0321958
Age	Covariate	MeetTwin	Social activity	-0.0317484	0.0317484
Age	Covariate	WorkMemo	Cognitive function	0.0314150	0.0314150

Var1	Category	Var2	Category	Partial correlation	Absolute value
Exercised	Physical activity	Outlook	Depressive symptom	-0.0313255	0.0313255
Sudoku	Intellectual activity	WorkMemo	Cognitive function	0.0308764	0.0308764
GoParty	Social activity	WorthNothing	Depressive symptom	-0.0304119	0.0304119
AuditAtt	Cognitive function	CategoryFluency	Cognitive function	0.0298481	0.0298481
AuditAtt	Cognitive function	HardWork	Physical activity	-0.0298218	0.0298218
Exercised	Physical activity	GoParty	Social activity	0.0287842	0.0287842
Sport	Physical activity	Tense	Depressive symptom	-0.0285701	0.0285701
HardWork	Physical activity	WorkMemo	Cognitive function	-0.0284918	0.0284918
Age	Covariate	Outlook	Depressive symptom	0.0272157	0.0272157
Sport	Physical activity	WriteStory	Intellectual activity	0.0269987	0.0269987
DelayedRecall	Cognitive function	Newspaper	Intellectual activity	-0.0266447	0.0266447
Learning	Cognitive function	Library	Intellectual activity	0.0264423	0.0264423
GoParty	Social activity	Library	Intellectual activity	0.0263125	0.0263125
AuditAtt	Cognitive function	Learning	Cognitive function	0.0262327	0.0262327
HappyFre	Depressive symptom	WorthNothing	Depressive symptom	0.0259494	0.0259494
Association	Social activity	Newspaper	Intellectual activity	0.0259158	0.0259158
GoParty	Social activity	WriteStory	Intellectual activity	0.0255973	0.0255973
HappyNow	Depressive symptom	WriteStory	Intellectual activity	0.0251031	0.0251031
Diner	Social activity	Newspaper	Intellectual activity	0.0245699	0.0245699
Bicycle	Physical activity	CategoryFluency	Cognitive function	0.0232221	0.0232221
HappyNow	Depressive symptom	Newspaper	Intellectual activity	-0.0221540	0.0221540
GoParty	Social activity	Museum	Intellectual activity	0.0218825	0.0218825
Nervous	Depressive symptom	Sad	Depressive symptom	0.0215679	0.0215679
Cinema	Intellectual activity	GoParty	Social activity	0.0210123	0.0210123
Books	Intellectual activity	Nervous	Depressive symptom	-0.0201640	0.0201640
CategoryFluency	Cognitive function	WorkMemo	Cognitive function	0.0196174	0.0196174
WorkMemo	Cognitive function	WriteStory	Intellectual activity	0.0188797	0.0188797
CategoryFluency	Cognitive function	Sport	Physical activity	0.0187534	0.0187534
Lonely	Depressive symptom	Outlook	Depressive symptom	0.0187352	0.0187352
Association	Social activity	Sad	Depressive symptom	-0.0185778	0.0185778
DelayedRecall	Cognitive function	Outlook	Depressive symptom	-0.0183782	0.0183782
DelayedRecall	Cognitive function	Exercised	Physical activity	0.0181269	0.0181269
Lonely	Depressive symptom	Newspaper	Intellectual activity	-0.0179681	0.0179681
CategoryFluency	Cognitive function	HappyNow	Depressive symptom	0.0172267	0.0172267
Phone	Social activity	WorthNothing	Depressive symptom	-0.0170763	0.0170763
BriskWalk	Physical activity	WorthNothing	Depressive symptom	-0.0166496	0.0166496
Books	Intellectual activity	GoParty	Social activity	0.0164940	0.0164940
Learning	Cognitive function	WriteStory	Intellectual activity	0.0163664	0.0163664
BriskWalk	Physical activity	HappyNow	Depressive symptom	-0.0163091	0.0163091
BriskWalk	Physical activity	HappyFre	Depressive symptom	-0.0158621	0.0158621
GoParty	Social activity	HardWork	Physical activity	0.0149430	0.0149430
BriskWalk	Physical activity	Diner	Social activity	0.0147772	0.0147772
Restaurant	Social activity	Yoga	Physical activity	0.0144453	0.0144453

Var1	Category	Var2	Category	Partial correlation	Absolute value
CurrConsAlc	Covariate	GoParty	Social activity	0.0144370	0.0144370
HappyNow	Depressive symptom	Library	Intellectual activity	0.0143070	0.0143070
Bicycle	Physical activity	Outlook	Depressive symptom	-0.0139321	0.0139321
Association	Social activity	Phone	Social activity	0.0137996	0.0137996
WriteStory	Intellectual activity	Yoga	Physical activity	0.0136507	0.0136507
Cinema	Intellectual activity	Diner	Social activity	0.0132796	0.0132796
GoParty	Social activity	Yoga	Physical activity	0.0131098	0.0131098
CurrConsAlc	Covariate	HappyFre	Depressive symptom	-0.0130843	0.0130843
Outlook	Depressive symptom	Sport	Physical activity	-0.0121946	0.0121946
CategoryFluency	Cognitive function	Exercised	Physical activity	0.0121791	0.0121791
HardWork	Physical activity	PsychMotorSpeed	Cognitive function	-0.0120754	0.0120754
Sport	Physical activity	Yoga	Physical activity	0.0118233	0.0118233
FriendsDiner	Social activity	WorthNothing	Depressive symptom	-0.0111974	0.0111974
Diner	Social activity	HappyFre	Depressive symptom	-0.0108540	0.0108540
CategoryFluency	Cognitive function	Nervous	Depressive symptom	-0.0105247	0.0105247
Library	Intellectual activity	Restaurant	Social activity	0.0102063	0.0102063
Association	Social activity	Books	Intellectual activity	0.0101214	0.0101214
Association	Social activity	WorthNothing	Depressive symptom	-0.0100976	0.0100976
Nervous	Depressive symptom	WorthLiving	Depressive symptom	0.0100718	0.0100718
Diner	Social activity	HardWork	Physical activity	0.0099819	0.0099819
Exercised	Physical activity	WriteStory	Intellectual activity	0.0098886	0.0098886
Bicycle	Physical activity	Museum	Intellectual activity	0.0091939	0.0091939
Age	Covariate	CurrConsAlc	Covariate	0.0091554	0.0091554
Learning	Cognitive function	Museum	Intellectual activity	0.0085361	0.0085361
Restaurant	Social activity	WriteStory	Intellectual activity	0.0084063	0.0084063
HardWork	Physical activity	Learning	Cognitive function	-0.0083642	0.0083642
GoParty	Social activity	Nervous	Depressive symptom	-0.0082319	0.0082319
Diner	Social activity	Nervous	Depressive symptom	-0.0080224	0.0080224
Age	Covariate	Restaurant	Social activity	-0.0077620	0.0077620
Bicycle	Physical activity	Restaurant	Social activity	0.0076374	0.0076374
BriskWalk	Physical activity	PhoneTwin	Social activity	0.0074944	0.0074944
HappyNow	Depressive symptom	Sudoku	Intellectual activity	-0.0074014	0.0074014
Bicycle	Physical activity	Newspaper	Intellectual activity	0.0073228	0.0073228
Association	Social activity	Tense	Depressive symptom	-0.0068890	0.0068890
Association	Social activity	Exercised	Physical activity	0.0068574	0.0068574
Bicycle	Physical activity	WorthNothing	Depressive symptom	-0.0067256	0.0067256
Newspaper	Intellectual activity	Yoga	Physical activity	0.0067099	0.0067099
Courses	Intellectual activity	DelayedRecall	Cognitive function	0.0062271	0.0062271
Nervous	Depressive symptom	Newspaper	Intellectual activity	-0.0060518	0.0060518
BriskWalk	Physical activity	Sad	Depressive symptom	-0.0059223	0.0059223
CategoryFluency	Cognitive function	Museum	Intellectual activity	0.0055191	0.0055191
Library	Intellectual activity	Outlook	Depressive symptom	-0.0054334	0.0054334
Books	Intellectual activity	CategoryFluency	Cognitive function	0.0053317	0.0053317

Var1	Category	Var2	Category	Partial correlation	Absolute value
DelayedRecall	Cognitive function	WorkMemo	Cognitive function	0.0052875	0.0052875
Exercised	Physical activity	HardWork	Physical activity	0.0052874	0.0052874
AuditAtt	Cognitive function	PsychMotorSpeed	Cognitive function	0.0052097	0.0052097
Association	Social activity	Outlook	Depressive symptom	-0.0050938	0.0050938
Library	Intellectual activity	WorkMemo	Cognitive function	0.0049280	0.0049280
Association	Social activity	Cinema	Intellectual activity	0.0047877	0.0047877
Library	Intellectual activity	PsychMotorSpeed	Cognitive function	0.0043075	0.0043075
Diner	Social activity	Yoga	Physical activity	0.0042707	0.0042707
Exercised	Physical activity	HappyFre	Depressive symptom	-0.0042643	0.0042643
Library	Intellectual activity	Yoga	Physical activity	0.0039781	0.0039781
Diner	Social activity	WorthNothing	Depressive symptom	-0.0036872	0.0036872
AuditAtt	Cognitive function	Diner	Social activity	0.0036587	0.0036587
Cinema	Intellectual activity	Learning	Cognitive function	0.0036177	0.0036177
Books	Intellectual activity	Tense	Depressive symptom	-0.0035449	0.0035449
GoParty	Social activity	Phone	Social activity	0.0033645	0.0033645
Courses	Intellectual activity	PhoneTwin	Social activity	-0.0032927	0.0032927
Outlook	Depressive symptom	Restaurant	Social activity	-0.0023302	0.0023302
Courses	Intellectual activity	Yoga	Physical activity	0.0022335	0.0022335
Phone	Social activity	WriteStory	Intellectual activity	0.0021242	0.0021242
Association	Social activity	PsychMotorSpeed	Cognitive function	0.0016451	0.0016451
Age	Covariate	Nervous	Depressive symptom	0.0014992	0.0014992
HappyFre	Depressive symptom	Phone	Social activity	-0.0009168	0.0009168
Cinema	Intellectual activity	DelayedRecall	Cognitive function	0.0006945	0.0006945
Nervous	Depressive symptom	Sport	Physical activity	-0.0004859	0.0004859
FriendsDiner	Social activity	WorkMemo	Cognitive function	-0.0003578	0.0003578

Table S10 Edges of men network

Var1	Category	Var2	Category	Partial correlation	Absolute value
MeetTwin	Social activity	PhoneTwin	Social activity	0.5389336	0.5389336
DelayedRecall	Cognitive function	Learning	Cognitive function	0.4544895	0.4544895
Exercised	Physical activity	Sport	Physical activity	0.4152484	0.4152484
Diner	Social activity	FriendsDiner	Social activity	0.3520399	0.3520399
AuditAtt	Cognitive function	WorkMemo	Cognitive function	0.3049077	0.3049077
PsychMotorSpeed	Cognitive function	WorkMemo	Cognitive function	0.2952140	0.2952140
Exercised	Physical activity	Yoga	Physical activity	0.2496209	0.2496209
HappyFre	Depressive symptom	HappyNow	Depressive symptom	0.2387922	0.2387922
Phone	Social activity	PhoneTwin	Social activity	0.2224910	0.2224910
HappyFre	Depressive symptom	Sad	Depressive symptom	0.2153245	0.2153245
FriendsDiner	Social activity	Phone	Social activity	0.2103826	0.2103826
Cinema	Intellectual activity	Museum	Intellectual activity	0.2077205	0.2077205
Lonely	Depressive symptom	Sad	Depressive symptom	0.1948678	0.1948678
Association	Social activity	Courses	Intellectual activity	0.1941183	0.1941183
WorthLiving	Depressive symptom	WorthNothing	Depressive symptom	0.1916910	0.1916910
HappyNow	Depressive symptom	Outlook	Depressive symptom	0.1886418	0.1886418
Cinema	Intellectual activity	Restaurant	Social activity	0.1884488	0.1884488
GoParty	Social activity	Restaurant	Social activity	0.1874405	0.1874405
Books	Intellectual activity	WriteStory	Intellectual activity	0.1778584	0.1778584
Sad	Depressive symptom	WorthLiving	Depressive symptom	0.1748494	0.1748494
Courses	Intellectual activity	WriteStory	Intellectual activity	0.1725627	0.1725627
Books	Intellectual activity	Library	Intellectual activity	0.1697058	0.1697058
Age	Covariate	Newspaper	Intellectual activity	0.1579807	0.1579807
Sad	Depressive symptom	Tense	Depressive symptom	0.1525389	0.1525389
Association	Social activity	GoParty	Social activity	0.1455698	0.1455698
HappyNow	Depressive symptom	Sad	Depressive symptom	0.1443165	0.1443165
HappyFre	Depressive symptom	Outlook	Depressive symptom	0.1393351	0.1393351
Courses	Intellectual activity	Museum	Intellectual activity	0.1369537	0.1369537
Nervous	Depressive symptom	Tense	Depressive symptom	0.1309905	0.1309905
Diner	Social activity	GoParty	Social activity	0.1278706	0.1278706
Age	Covariate	BriskWalk	Physical activity	0.1257619	0.1257619
Bicycle	Physical activity	Exercised	Physical activity	0.1243041	0.1243041
HappyNow	Depressive symptom	Lonely	Depressive symptom	0.1190062	0.1190062
Sad	Depressive symptom	WorthNothing	Depressive symptom	0.1189077	0.1189077
CategoryFluency	Cognitive function	DelayedRecall	Cognitive function	0.1184965	0.1184965
Diner	Social activity	Restaurant	Social activity	0.1150520	0.1150520
Age	Covariate	DelayedRecall	Cognitive function	-0.1142206	0.1142206
Bicycle	Physical activity	BriskWalk	Physical activity	0.1137922	0.1137922
Bicycle	Physical activity	Sport	Physical activity	0.1129656	0.1129656
Library	Intellectual activity	WriteStory	Intellectual activity	0.1128539	0.1128539
CategoryFluency	Cognitive function	Learning	Cognitive function	0.1084022	0.1084022

Var1	Category	Var2	Category	Partial correlation	Absolute value
Books	Intellectual activity	Museum	Intellectual activity	0.1014911	0.1014911
HappyNow	Depressive symptom	Tense	Depressive symptom	0.0993630	0.0993630
Courses	Intellectual activity	Library	Intellectual activity	0.0980714	0.0980714
Nervous	Depressive symptom	WorthLiving	Depressive symptom	0.0937898	0.0937898
Books	Intellectual activity	Courses	Intellectual activity	0.0933394	0.0933394
Lonely	Depressive symptom	Tense	Depressive symptom	0.0925902	0.0925902
Cinema	Intellectual activity	Courses	Intellectual activity	0.0925634	0.0925634
Outlook	Depressive symptom	WorthLiving	Depressive symptom	0.0905715	0.0905715
CategoryFluency	Cognitive function	Library	Intellectual activity	0.0887369	0.0887369
CategoryFluency	Cognitive function	PsychMotorSpeed	Cognitive function	0.0882372	0.0882372
Sudoku	Intellectual activity	WorkMemo	Cognitive function	0.0866925	0.0866925
Books	Intellectual activity	Sudoku	Intellectual activity	0.0843004	0.0843004
Tense	Depressive symptom	WorthNothing	Depressive symptom	0.0822655	0.0822655
Books	Intellectual activity	Newspaper	Intellectual activity	0.0795141	0.0795141
Age	Covariate	MeetTwin	Social activity	-0.0780107	0.0780107
Library	Intellectual activity	Phone	Social activity	0.0773439	0.0773439
Learning	Cognitive function	WorkMemo	Cognitive function	0.0753635	0.0753635
HappyFre	Depressive symptom	WorthLiving	Depressive symptom	0.0750537	0.0750537
Nervous	Depressive symptom	Outlook	Depressive symptom	0.0738955	0.0738955
Age	Covariate	CategoryFluency	Cognitive function	-0.0705810	0.0705810
HappyFre	Depressive symptom	WorthNothing	Depressive symptom	0.0690690	0.0690690
FriendsDiner	Social activity	Restaurant	Social activity	0.0687810	0.0687810
Outlook	Depressive symptom	Sad	Depressive symptom	0.0674408	0.0674408
GoParty	Social activity	Phone	Social activity	0.0667915	0.0667915
Lonely	Depressive symptom	WorthNothing	Depressive symptom	0.0648693	0.0648693
AuditAtt	Cognitive function	PsychMotorSpeed	Cognitive function	0.0641965	0.0641965
Museum	Intellectual activity	Restaurant	Social activity	0.0635384	0.0635384
Cinema	Intellectual activity	Library	Intellectual activity	0.0631306	0.0631306
Age	Covariate	Sport	Physical activity	-0.0602126	0.0602126
BriskWalk	Physical activity	Yoga	Physical activity	0.0591962	0.0591962
Association	Social activity	FriendsDiner	Social activity	0.0578891	0.0578891
FriendsDiner	Social activity	GoParty	Social activity	0.0577801	0.0577801
BriskWalk	Physical activity	Museum	Intellectual activity	0.0571132	0.0571132
Age	Covariate	HappyNow	Depressive symptom	-0.0560205	0.0560205
Museum	Intellectual activity	WriteStory	Intellectual activity	0.0537907	0.0537907
Learning	Cognitive function	WriteStory	Intellectual activity	0.0511152	0.0511152
CategoryFluency	Cognitive function	WorkMemo	Cognitive function	0.0507306	0.0507306
Lonely	Depressive symptom	WorthLiving	Depressive symptom	0.0493780	0.0493780
Age	Covariate	Library	Intellectual activity	-0.0492115	0.0492115
Age	Covariate	Learning	Cognitive function	-0.0490316	0.0490316
Bicycle	Physical activity	Cinema	Intellectual activity	0.0485072	0.0485072
Diner	Social activity	Lonely	Depressive symptom	-0.0484206	0.0484206
Exercised	Physical activity	Library	Intellectual activity	0.0478403	0.0478403

Var1	Category	Var2	Category	Partial correlation	Absolute value
Museum	Intellectual activity	Newspaper	Intellectual activity	0.0474398	0.0474398
FriendsDiner	Social activity	MeetTwin	Social activity	0.0462225	0.0462225
DelayedRecall	Cognitive function	PsychMotorSpeed	Cognitive function	0.0446206	0.0446206
DelayedRecall	Cognitive function	Library	Intellectual activity	0.0439099	0.0439099
Cinema	Intellectual activity	GoParty	Social activity	0.0424862	0.0424862
Courses	Intellectual activity	GoParty	Social activity	0.0420960	0.0420960
HardWork	Physical activity	Library	Intellectual activity	-0.0415421	0.0415421
AuditAtt	Cognitive function	Learning	Cognitive function	0.0415414	0.0415414
Books	Intellectual activity	Diner	Social activity	0.0414521	0.0414521
GoParty	Social activity	Sport	Physical activity	0.0413358	0.0413358
Sport	Physical activity	Yoga	Physical activity	0.0412473	0.0412473
Diner	Social activity	Phone	Social activity	0.0385051	0.0385051
Cinema	Intellectual activity	Diner	Social activity	0.0381640	0.0381640
WriteStory	Intellectual activity	Yoga	Physical activity	0.0381186	0.0381186
Association	Social activity	WriteStory	Intellectual activity	0.0373311	0.0373311
Library	Intellectual activity	PsychMotorSpeed	Cognitive function	0.0372551	0.0372551
HappyFre	Depressive symptom	Lonely	Depressive symptom	0.0370956	0.0370956
BriskWalk	Physical activity	Newspaper	Intellectual activity	0.0368153	0.0368153
Nervous	Depressive symptom	WorthNothing	Depressive symptom	0.0357115	0.0357115
Age	Covariate	Museum	Intellectual activity	0.0355168	0.0355168
HappyNow	Depressive symptom	WorthLiving	Depressive symptom	0.0354181	0.0354181
BriskWalk	Physical activity	Exercised	Physical activity	0.0354171	0.0354171
PsychMotorSpeed	Cognitive function	Sudoku	Intellectual activity	0.0343584	0.0343584
Library	Intellectual activity	Restaurant	Social activity	0.0341041	0.0341041
AuditAtt	Cognitive function	HardWork	Physical activity	-0.0339431	0.0339431
Books	Intellectual activity	GoParty	Social activity	0.0329778	0.0329778
Association	Social activity	Exercised	Physical activity	0.0326450	0.0326450
Learning	Cognitive function	Library	Intellectual activity	0.0322598	0.0322598
Courses	Intellectual activity	Restaurant	Social activity	0.0322589	0.0322589
CategoryFluency	Cognitive function	WriteStory	Intellectual activity	0.0312462	0.0312462
Age	Covariate	Sudoku	Intellectual activity	0.0310817	0.0310817
Library	Intellectual activity	Sport	Physical activity	0.0304403	0.0304403
BriskWalk	Physical activity	Diner	Social activity	0.0294567	0.0294567
GoParty	Social activity	Yoga	Physical activity	0.0294539	0.0294539
Library	Intellectual activity	Museum	Intellectual activity	0.0290203	0.0290203
HappyNow	Depressive symptom	WorthNothing	Depressive symptom	0.0290145	0.0290145
AuditAtt	Cognitive function	CategoryFluency	Cognitive function	0.0287132	0.0287132
Phone	Social activity	Restaurant	Social activity	0.0271268	0.0271268
Age	Covariate	Exercised	Physical activity	-0.0265549	0.0265549
Courses	Intellectual activity	Diner	Social activity	0.0253972	0.0253972
Cinema	Intellectual activity	Exercised	Physical activity	0.0248980	0.0248980
HappyFre	Depressive symptom	Tense	Depressive symptom	0.0245114	0.0245114
Lonely	Depressive symptom	Newspaper	Intellectual activity	-0.0213292	0.0213292

Var1	Category	Var2	Category	Partial correlation	Absolute value
BriskWalk	Physical activity	Learning	Cognitive function	-0.0211967	0.0211967
Cinema	Intellectual activity	Sport	Physical activity	0.0204731	0.0204731
Courses	Intellectual activity	Yoga	Physical activity	0.0199139	0.0199139
HardWork	Physical activity	Sport	Physical activity	0.0198277	0.0198277
Lonely	Depressive symptom	Outlook	Depressive symptom	0.0196700	0.0196700
Outlook	Depressive symptom	Tense	Depressive symptom	0.0194005	0.0194005
Association	Social activity	Cinema	Intellectual activity	0.0193462	0.0193462
Books	Intellectual activity	Restaurant	Social activity	0.0190952	0.0190952
Association	Social activity	Museum	Intellectual activity	0.0189778	0.0189778
Courses	Intellectual activity	Learning	Cognitive function	0.0188743	0.0188743
GoParty	Social activity	Outlook	Depressive symptom	-0.0184020	0.0184020
Association	Social activity	Sport	Physical activity	0.0183887	0.0183887
FriendsDiner	Social activity	HardWork	Physical activity	0.0180223	0.0180223
Exercised	Physical activity	WriteStory	Intellectual activity	0.0174048	0.0174048
Books	Intellectual activity	BriskWalk	Physical activity	0.0172143	0.0172143
Outlook	Depressive symptom	WorthNothing	Depressive symptom	0.0157894	0.0157894
Association	Social activity	Yoga	Physical activity	0.0151495	0.0151495
Exercised	Physical activity	Restaurant	Social activity	0.0150775	0.0150775
HardWork	Physical activity	MeetTwin	Social activity	0.0143626	0.0143626
Cinema	Intellectual activity	WriteStory	Intellectual activity	0.0137453	0.0137453
Library	Intellectual activity	Sudoku	Intellectual activity	0.0135961	0.0135961
CategoryFluency	Cognitive function	Nervous	Depressive symptom	-0.0135100	0.0135100
Newspaper	Intellectual activity	WorthNothing	Depressive symptom	-0.0129866	0.0129866
Courses	Intellectual activity	Outlook	Depressive symptom	-0.0124933	0.0124933
HardWork	Physical activity	WorkMemo	Cognitive function	-0.0122358	0.0122358
DelayedRecall	Cognitive function	WorkMemo	Cognitive function	0.0120396	0.0120396
GoParty	Social activity	Newspaper	Intellectual activity	0.0112455	0.0112455
Courses	Intellectual activity	Exercised	Physical activity	0.0111356	0.0111356
HardWork	Physical activity	Yoga	Physical activity	0.0109478	0.0109478
Outlook	Depressive symptom	Sport	Physical activity	-0.0108514	0.0108514
Library	Intellectual activity	WorkMemo	Cognitive function	0.0105026	0.0105026
Exercised	Physical activity	GoParty	Social activity	0.0099848	0.0099848
AuditAtt	Cognitive function	Library	Intellectual activity	0.0097767	0.0097767
Bicycle	Physical activity	Yoga	Physical activity	0.0097595	0.0097595
CategoryFluency	Cognitive function	Exercised	Physical activity	0.0093078	0.0093078
Diner	Social activity	Museum	Intellectual activity	0.0092698	0.0092698
Cinema	Intellectual activity	Phone	Social activity	0.0091114	0.0091114
BriskWalk	Physical activity	DelayedRecall	Cognitive function	-0.0086812	0.0086812
WorkMemo	Cognitive function	WriteStory	Intellectual activity	0.0080948	0.0080948
HardWork	Physical activity	PsychMotorSpeed	Cognitive function	-0.0073302	0.0073302
Nervous	Depressive symptom	Sad	Depressive symptom	0.0071772	0.0071772
Phone	Social activity	WriteStory	Intellectual activity	0.0066373	0.0066373
Association	Social activity	Books	Intellectual activity	0.0065936	0.0065936

Var1	Category	Var2	Category	Partial correlation	Absolute value
HappyFre	Depressive symptom	Nervous	Depressive symptom	0.0064400	0.0064400
Books	Intellectual activity	Cinema	Intellectual activity	0.0063270	0.0063270
BriskWalk	Physical activity	GoParty	Social activity	0.0060299	0.0060299
Bicycle	Physical activity	Courses	Intellectual activity	0.0059639	0.0059639
Outlook	Depressive symptom	WriteStory	Intellectual activity	-0.0057590	0.0057590
Lonely	Depressive symptom	Nervous	Depressive symptom	0.0049573	0.0049573
BriskWalk	Physical activity	Courses	Intellectual activity	0.0049289	0.0049289
BriskWalk	Physical activity	HappyNow	Depressive symptom	-0.0047255	0.0047255
Diner	Social activity	Outlook	Depressive symptom	-0.0036260	0.0036260
Books	Intellectual activity	Outlook	Depressive symptom	-0.0033894	0.0033894
AuditAtt	Cognitive function	Exercised	Physical activity	0.0030156	0.0030156
GoParty	Social activity	HardWork	Physical activity	0.0024712	0.0024712
Bicycle	Physical activity	Sad	Depressive symptom	-0.0024109	0.0024109
Exercised	Physical activity	Outlook	Depressive symptom	-0.0021237	0.0021237
Age	Covariate	Cinema	Intellectual activity	-0.0015762	0.0015762
HardWork	Physical activity	WriteStory	Intellectual activity	-0.0013668	0.0013668
Museum	Intellectual activity	Yoga	Physical activity	0.0000628	0.0000628

4. Details of edges between depressive symptoms and other variables

Table S11 Non-significant edges connecting the depressive symptoms and other variables in women network

Edge	Other node category	Partial correlation	Absolute value
Outlook-GoParty	Social activity	-0.0414034	0.0414034
Outlook-Diner	Social activity	-0.0407012	0.0407012
Lonely-Diner	Social activity	-0.0334351	0.0334351
Outlook-Exercised	Physical activity	-0.0313255	0.0313255
WorthNothing-GoParty	Social activity	-0.0304119	0.0304119
Nervous-Books	Intellectual activity	-0.0201640	0.0201640
Lonely-Newspaper	Intellectual activity	-0.0179681	0.0179681
HappyNow-CategoryFluency	Cognitive function	0.0172267	0.0172267
WorthNothing-BriskWalk	Physical activity	-0.0166496	0.0166496
HappyNow-BriskWalk	Physical activity	-0.0163091	0.0163091
HappyFre-BriskWalk	Physical activity	-0.0158621	0.0158621
HappyNow-Library	Intellectual activity	0.0143070	0.0143070
Outlook-Sport	Physical activity	-0.0121946	0.0121946
HappyFre-Diner	Social activity	-0.0108540	0.0108540
Nervous-CategoryFluency	Cognitive function	-0.0105247	0.0105247
WorthNothing-Association	Social activity	-0.0100976	0.0100976
Nervous-GoParty	Social activity	-0.0082319	0.0082319
Nervous-Diner	Social activity	-0.0080224	0.0080224
Tense-Association	Social activity	-0.0068890	0.0068890
WorthNothing-Bicycle	Physical activity	-0.0067256	0.0067256
Nervous-Newspaper	Intellectual activity	-0.0060518	0.0060518
Sad-BriskWalk	Physical activity	-0.0059223	0.0059223
Outlook-Library	Intellectual activity	-0.0054334	0.0054334
Outlook-Association	Social activity	-0.0050938	0.0050938
HappyFre-Exercised	Physical activity	-0.0042643	0.0042643
WorthNothing-Diner	Social activity	-0.0036872	0.0036872
Tense-Books	Intellectual activity	-0.0035449	0.0035449
Outlook-Restaurant	Social activity	-0.0023302	0.0023302
Nervous-Sport	Physical activity	-0.0004859	0.0004859

Table S12 Non-significant edges connecting the depressive symptoms and other variables in men network.

Edge	Other node category	Partial correlation	Absolute value
Lonely-Diner	Social activity	-0.0484206	0.0484206
Lonely-Newspaper	Intellectual activity	-0.0213292	0.0213292
Outlook-GoParty	Social activity	-0.0184020	0.0184020
Nervous-CategoryFluency	Cognitive function	-0.0135100	0.0135100
WorthNothing-Newspaper	Intellectual activity	-0.0129866	0.0129866
Outlook-Sport	Physical activity	-0.0108514	0.0108514
Outlook-WriteStory	Intellectual activity	-0.0057590	0.0057590
HappyNow-BriskWalk	Physical activity	-0.0047255	0.0047255
Outlook-Diner	Social activity	-0.0036260	0.0036260
Outlook-Books	Intellectual activity	-0.0033894	0.0033894
Sad-Bicycle	Physical activity	-0.0024109	0.0024109
Outlook-Exercised	Physical activity	-0.0021237	0.0021237
